# Scoring Algorithms to Optimize Viewing Time Measures of Sexual Interest in Children

**DOI:** 10.1007/s10508-026-03490-6

**Published:** 2026-08-08

**Authors:** Lilli Meißner, Alexander F. Schmidt, Rainer Banse

**Affiliations:** 1 Social and Legal Psychology, Institute of Psychology, Rhenish Friedrich Wilhelm University of Bonn, Kaiser-Karl-Ring 9, D-53111 Bonn, Germany; 2 Social and Legal Psychology, Institute of Psychology, Johannes Gutenberg-University Mainz, Mainz, Germany

**Keywords:** Pedohebephilia, Sexual interest, Viewing time, Pedophilia, Penile plethsymography, DSM-5

## Abstract

Viewing times (VTs) are established indirect measures of sexual interests. They are based on the effect that stimuli of sexually attractive target groups are scrutinized longer than stimuli of non-attractive groups in sexual attractiveness rating tasks. When assessing sexual interest in children and adults, decision latencies for target sexual maturity categories are typically compared by subtracting average latencies for children from average latencies of adults. In this study, several ways of improving the scoring algorithm were explored (e.g., using standardized differences, excluding the longest VTs of each category from the scoring, or conducting a profile analysis based on peaks in certain target categories). Scoring algorithms were compared in terms of reliability, convergent validity, and the influence of confounding variables in a secondary analysis of a German male community sample (*N* = 8554). Additionally, the influence of the scoring on the number of participants who would be classified as exhibiting (increased levels of) sexual interest in children was investigated. The VT task’s validity proved to be quite robust to changes in scoring, but the reliability and classification proportions differed substantially between scores. The best performing score was the mean difference of adult and child target groups with the exclusion of the longest VT in each target category. The results imply that the scoring approach should be chosen based on the research or diagnostic goal of using the VT task (e.g., whether one prioritizes sensitivity or specificity). The study underlines the influence of measurement and scoring approach on prevalence estimates of (increased) sexual interest in children.

**Supplementary Information:**

The online version contains supplementary material available at https://doi.org/10.1007/s10508-026-03490-6.

## Introduction

Pedohebephilia, a persistent sexual interest in prepubescent and pubescent children, is one of the major risk factors for sexual recidivism among people who have sexually offended against children (Seto et al., 2023). Thus, its clinical assessment is of prime interest in the evaluation and treatment of persons with sexual offense histories as well as in secondary prevention attempts. At the same time, pedohebephilia is among the most strongly stigmatized psychological disorders due to its conflation with sexual offending against children (e.g., Lehmann et al., 2021) although empirically not every individual with pedohebephilic interest will necessarily sexually abuse children (e.g., Dombert et al., 2016) and not every person who has sexually abused children exhibits sexual preferences for children over adults (e.g., Schmidt et al., 2013). It is due to the important forensic implications and the social stigmatization of pedohebephilic sexual inclinations that their assessment is crucial but fraught with difficulties.

Since self-report statements can be easily biased by impression management and social desirability, several other instruments for a more objective measurement of sexual interest have been introduced in the last decades (Schmidt et al., 2015). Penile plethysmography (PPG) is often regarded as the preferable option in terms of its psychometric properties (McPhail et al., 2019). However, typical drawbacks of PPG besides its high intrusiveness include technical challenges, need of expensive equipment, and lacking standardization (Seto, 2018). Another simple to apply measurement approach is computer based and relies on assessing response latencies (Schmidt & Banse, 2022). The most prominent examples are the Implicit Association Test and viewing time (VT) measures (Pedneault et al., 2021).

### Viewing Time Measures of Sexual Interest

Using the amount of time an individual spends to look at (sexual) pictures to indirectly assess their sexual interest was first described by Rosenzweig (1942). Based on his findings, Zamansky (1956) used VT measures to assess a person’s sexual interest in different target groups (i.e., sexual orientation). He showed that gay men looked longer at stimuli showing men than women, whereas for straight men it was the other way around. Only much later, it was experimentally shown that the underlying effect is a cognitive epiphenomenon of the necessary sexual attractiveness rating task as one needs to take more features into account (and therefore produce longer response latencies) to reach a decision in case of a more sexually attractive target as opposed to a less sexually attractive target (Imhoff et al., 2010, 2012; Schmidt et al., 2021).

Using VT measures to assess men’s sexual interest in children was firstly validated by Abel et al. (1994). Since then, its validity has been further supported by comparing VT results with other measures as well as using VT to discriminate between individuals convicted for child sexual offenses and individuals with other convictions, subtypes of persons who have sexually offended against children, and community males with and without pedohebephilia (Abel et al., 2004; Banse et al., 2010; Harris et al., 1996; Jahnke et al., 2022; Laws & Gress, 2004; Letourneau, 2002; Schmidt et al., 2014; Stinson & Becker, 2008; for meta-analytic overviews, see Pedneault et al., 2021; Schmidt et al., 2017). Its criterion and convergent/construct validity are comparable to PPG (Schmidt & Banse, 2022) while its retest reliability has been shown to be even superior (Welsch et al., 2021). A first study has also shown promising results regarding the predictive link of VT to sexual reoffending (Gray et al., 2015). However, more research is needed to determine if it measures up to the predictive validity of PPG shown in a variety of samples (McPhail et al., 2019). Overall, VT has important advantages like requiring only a computer, being suitable for online surveys, drastically reducing examination times, and being less invasive due to producing valid results even without sexually explicit stimuli (e.g., Banse et al., 2010). The ease of VT applications combined with their favorable psychometric properties has resulted in its mentioning in the DSM-5 as a valid indirect assessment of pedophilic interest (American Psychiatric Association, 2013, p. 699).

### Current Scoring Algorithms

In research settings, one commonly used scoring method is based on absolute scores, i.e., mean VTs of each target category. Absolute scores do not control for the general VT (i.e., the average response speed), but this information may itself hold diagnostical value. Harris et al. (1996) demonstrated that persons without past offenses took almost twice as long to rate and thereby view the stimuli than people with sexual offense histories.

**Fig. 1:**
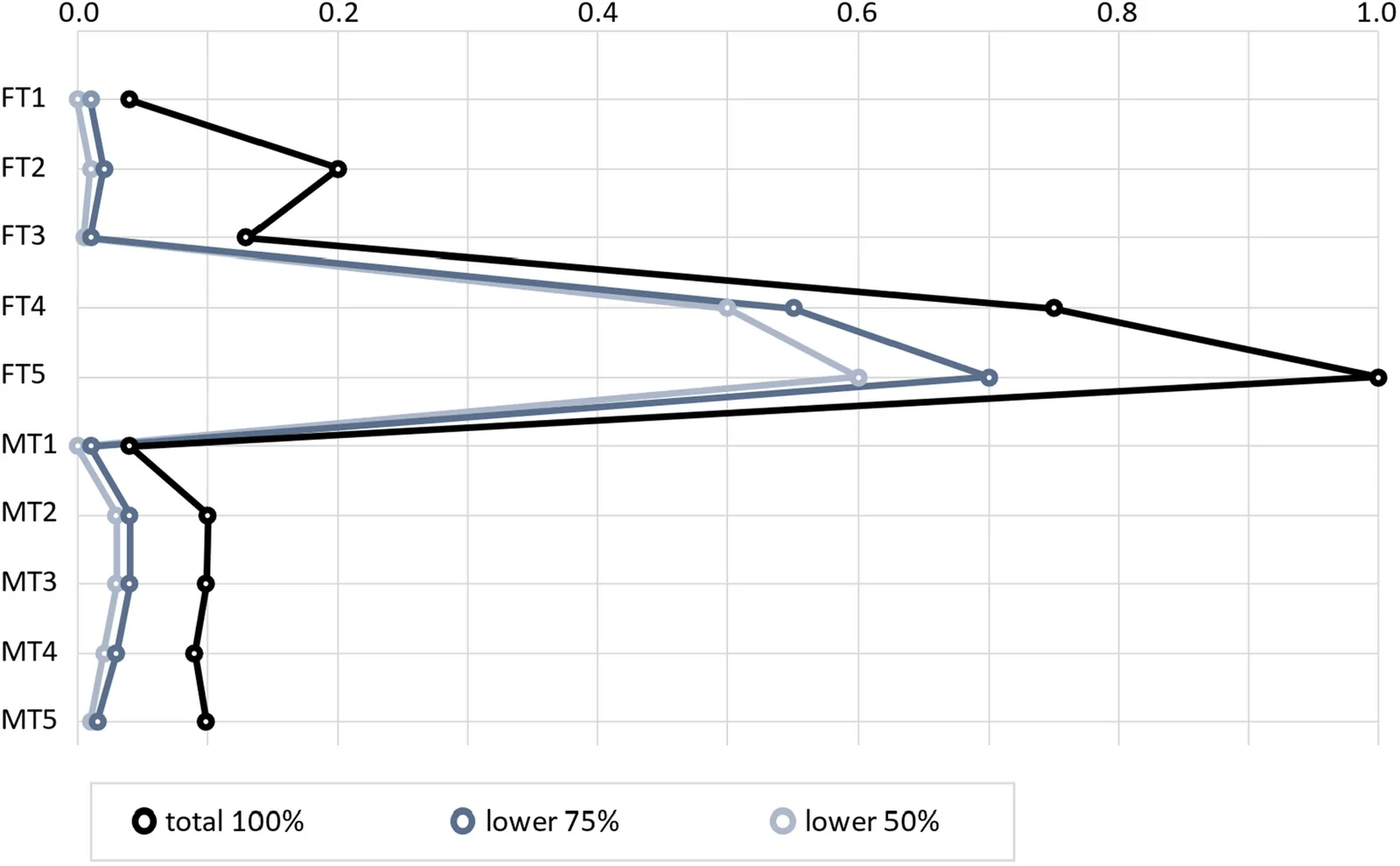
An example of an artificially rescaled EISIP viewing time Profile (0 = Lowest VT, 1 = Highest VT) used for single-case assessment. The y-axis refers to the age and sex categories of the stimuli: F = female, M = male, Tx = Tanner class x (e.g., FT1 = female Tanner 1)

When VT measures are applied in single-case assessment, in the case of the Explicit and Implicit Sexual Interest Profile (EISIP; Banse et al., 2010), the absolute VTs are aggregated in a graphic profile (Fig. 1) which can be analyzed regarding its peaks and lows across sexual target categories. The profile presents the average VTs for each category calculated with (1) all recorded VTs, (2) the lower 75% of VTs per category (i.e., excluding the highest latencies), and (3) the lower 50% of VTs per category. This practice of calculating three profiles with different subsets of VTs was only recently introduced to control for inconsistencies within categories (cf. Schmidt & Perkins, 2021 for the former practice without the exclusion of extreme values). Consequently, only unambiguous, distinct peaks that remain stable across all three profile lines are interpreted as indicative of sexual interest in the corresponding category. In the example provided in Fig. 1, the peak associated with young girls appears inconsistent, whereas the peak for adult women remains stable across all three profiles, suggesting a general sexual interest in adult women but not in young girls.

The most commonly applied (and most valid) score in research settings, however, is a difference index (Schmidt et al., 2017), i.e., subtracting the mean VT for child categories from the mean VT for adult categories. This relative score automatically controls for the general reaction time (mean VT of all stimuli) of the participant (Schmidt et al., 2014). Typically, the difference score also controls for the preferred gender in each age group if only the child and adult category with the highest mean VT irrespective of target sex is considered. One advantage all of the described scoring methods share is the aggregation of VTs for single stimuli into categories (Schmidt et al., 2017). This increases reliability as the results do not rely on secondary factors such as single outliers.

### Prevalence Estimates of Sexual Interest in Children

The true prevalence of pedophilia is (and likely remains) unknown, and estimates heavily depend on the sample, definition, and assessment criteria used. In the DSM-5, an upper limit of 3–5% in the male population is suggested; however, this refers to the diagnosis of a pedophilic disorder which requires having acted on the corresponding sexual fantasies or being severely distressed by them (American Psychiatric Association, 2013). Taking all men reporting some kind of sexual fantasy involving a child into account, in a recent systemic review Savoie et al. (2021) concluded that the mean prevalence estimations for sexual interest in children range from 2.1 to 24.0% as a function of different definitions of children (i.e., prepubescent/pubescent/under 18 years old), fantasies (i.e., finding anything about children sexually attractive/actively fantasizing about sexual activities with children/masturbating to these fantasies), and sexual interest (any interest vs. preference) as well as sample compositions.

After excluding studies with a high bias risk, Savoie et al. (2021) reported mean prevalence estimations of 2.1% for sexual interest (including a variety of sexual interest indicators such as sexual fantasies, arousal, contact with children, or diagnoses) in prepubescent children and 2.6% for sexual interest in pubescent children. When self-reported sexual fantasies involving children and adults were compared, only 0.1% of men indicated to have a sexual preference for children (Dombert et al., 2016; *N* = 8718 German community men). This fits with most men with pedophilic tendencies being non-exclusive (i.e., attracted to children and adults) and frequently having sex and relationships with adults (e.g., 65% married, separated, or divorced in Cohen & Galynker, 2002). Most community studies reporting frequencies of sexual interest in children to date relied on self-report measures, with the exception of Hall et al. (1995). Using PPG, over 25% of their community sample showed higher or similar physiological sexual arousal toward child stimuli compared to adult stimuli. However, their sample consisted of only 80 men.

When it comes to acting on their sexual fantasies, depending on sample 1.5–7.0% expressed an interest in having sexual contact with a child under twelve and 3.0–9.0% in using child sexual abuse material (CSAM) if it was guaranteed no one would find out (Briere & Runtz, 1989; Lehmann et al., 2025; Wurtele et al., 2014). Regarding actual child sexual abuse, a mean rate of 2.0% reported sexual contact with prepubescent or pubescent children (Savoie et al., 2021). Having watched CSAM reached higher estimates ranging between 2.4 and 21.0% (Dombert et al., 2016; Ray et al., 2014; Seto et al., 2015).

Considering these prevalence estimates, one has to keep in mind that none of the reported studies fulfill the requirements to assess the true prevalence of sexual interest in children in the population. To do so, the sample would have to be a “microcosm of the target population” (Groves et al., 2009, p. 62). While some of the discussed studies tried to collect large and representative samples, they do not actually cover the whole population when certain groups of people (1) do not have the possibility to participate due to the survey design (e.g., people without internet access or with low literacy skills), (2) do not have the capacity to participate (e.g., have limited time due to work and childcare responsibilities), or (3) choose not to participate because of the study’s contents or methods (e.g., because reading about sexuality does not agree with their religious beliefs). In ethical research, none of these groups can be forced to participate and thus are never represented in the actual samples. Hence, all reported frequencies of sexual interest in children should be interpreted as estimates and not “true” prevalences.

### Current Study

Research on pedophilia involving large community samples is rare, since most results are based on studies with individuals who have offense histories or convenience samples of, for example, university students. Because self-report is not the most reliable source when it comes to such a sensitive topic, the first aim of this study was to improve VT measures as a more objective behavioral assessment of sexual interest in children. Even though the use of VT measures is widespread, their scoring has never been systematically investigated or standardized. We suppose that the chosen scoring approach and its implication for the conceptualization of sexual interest in children can heavily influence the interpretation of the VT task. To this end, different ways of scoring the response latencies were explored, including computerizing the visual profile analysis of VTs in single-case assessment. The different scoring algorithms were compared in terms of their (1) convergent validity, (2) reliability, and (3) dependence on confounding variables. Furthermore, the resulting insights into scoring VT measures were expanded by exploring how many people in the sample would be classified as exhibiting increased sexual interest in children based on the self-report and different scoring approaches of the VT task. The review by Savoie et al. (2021) already shows how dependent prevalence estimations are on sample and measurement method. This study wanted to further investigate the influence of the scoring algorithm of the chosen measure—in this case the VT task—on the subsequent interpretation and implications of the results.

## Method

This study is a secondary analysis of data which were collected as part of the MiKADO-project (Osterheider et al., 2011). For an extensive overview of the procedure and measures, see Dombert et al. (2016) who describe the proportion of self-reported sexual interest in children in the sample. In related work using the same dataset, researchers have more deeply examined the scope of sex drive and hypersexuality (Klein et al., 2015), commercial sexual exploitation of children (Koops et al., 2017) as well as risk factors specifically for the group of men working with children (Turner et al., 2016). McPhail and Schmidt (2023) employed the same viewing time data as this study to evaluate the latent structure of pedohebephilia yielding a trichotomous taxonic structure.

Because this study was completely exploratory without any formulated hypotheses, no preregistration for the study and analysis plan were conducted. Consequently, the results should be interpreted as preliminary until they have been replicated.

### Participants

Participants were collected by a market research panel with the goal of the overall sample to be representative of the German population including all levels of education and age groups over 18. This aim was partially achieved with all groups being represented, although especially the age range from 30 to 49 years and higher education levels were slightly overrepresented (Dombert et al., 2016). Notably, however, in the original study neither age nor education was related to self-reported sexual interests in children.

Anonymity was an important aspect of this study since the participants were asked about past criminal acts as well as highly stigmatized sexual interests. Thus, survey answers were stored on a different server than the personal data needed for financial compensation. The participants received 20€ (approximately US$23.50) for needing median 19 min to finish the study. To assure voluntariness, the 17,917 invited men received an e-mail beforehand informing them about the topics covered in the questions as well as the possibility to stop the participation at any time by closing the browser tab. 10,538 of them started the survey; however, 493 participants decided in the end that they did not want their data to be analyzed. Of the remaining 10,045 valid cases, 1491 were excluded from the present analysis because their data were incomplete. The effective sample of 8554 male participants ranged from 18 to 89 years of age (*M* = 43.51, *SD* = 13.69) and included all employment and education levels. Questions about race or ethnicity are highly unusual in German-speaking countries and without existing compelling hypotheses had therefore not been included in the survey.

### Measures and Procedure

After consenting to the participation, the participants filled in demographic questions regarding their age, education level, and employment status. Subsequently, more self-report questions, the VT task, and another latency-based measure of sexual interest followed. In the following, the variables relevant to this study are explained.

#### Self-Report Measures

The self-report questions covered the following topics: use of CSAM, intention to engage in commercial sexual exploitation of children, and the self-assessed likelihood that one would ever sexually abuse a child. Additionally, an abbreviated version (24 instead of 40 items) of the Explicit Sexual Interest Questionnaire (ESIQ; Banse et al., 2010) was presented. The ESIQ can be split into two subscales, one about past sexual behavior since the age of 18 years (e.g., “I have sexually caressed a …”) and the other about sexual fantasies (e.g., “I get excited when I imagine that…stimulates me sexually”). Both scales include three items referring to the four target groups of men, women, girls, or boys (with children being defined as below 13 years of age). The participants could answer these questions with yes or no.

#### Viewing Time Measures

The VT task was taken from the EISIP and slightly modified (40 instead of 80 pictures). The participants were asked to rate the presented stimuli in terms of their subjectively perceived sexual attractiveness using a Likert-scale ranging from 1 (sexually unexciting) to 5 (sexually exciting). There was no time constraint on the rating, and the VT of each stimulus was recorded without the participants’ knowledge. The stimuli were taken from the Not-Real-People picture set (Pacific Psychological Assessment Cooperation, 2004) and showed the head and body of White Caucasian people in plain bathing clothes. The pictures were categorized by their sexual maturation according to Tanner (1990) with the Tanner categories 1 to 3 representing (pre)pubescent children, Tanner 4 postpubescent adolescents, and Tanner 5 postpubescent adults. In the present study, four pictures of each Tanner category (Tanner 1 to 5) target sex (male and female) were presented. In the following formulas and tables, the categories will be abbreviated with M indexing male, F female, and Tx number of the Tanner stage (e.g., male Tanner stage 3 = MT3).

### Scoring Algorithms

#### Absolute Scoring

All scores were calculated using SPSS; the syntax can be found in the online supplemental material. The first step of scoring the VTs was based on aggregating them into categories to increase their reliability. A mean VT for each Tanner stage × sex category was calculated, forming the *absolute scores* (Eq. 1).

1 $$ \overline{VT}_{x} $$

#### Difference Indices

Comparisons of the absolute VTs of different groups are commonly based on subtracting the absolute scores of (pre)pubescent child and postpubescent adult categories from each other. This *difference index (D)*, see Eq. 2, displays the sexual preference of a person by showing which group they are sexually preferring over the other assessed target groups. When the mean VT for adults is subtracted from the one for children, a score above 0 reflects a preference for children, a score of 0 no preference for either age group, and a score below 0 a preference for adults.

2 $$ D = \overline{VT}_{children} - \overline{VT}_{adults} $$

Several variations in this simple difference index were developed based on observations the authors made in single-case usage of the EISIP. Contrary to former analyses (Schmidt et al., 2014), *D* was correlated with the general processing speed (i.e., the mean of all VTs) in our sample. Thus, to reduce the influence of the general reaction time on the score a *standardized difference index (D*_*x̅*_*)* was produced by dividing *D* by the mean of all VTs (Eq. 3). An overview of all proposed scoring algorithms is presented in Table 1.

3 $$ D_{{\overline{X} }} = \frac{{\overline{VT}_{children} - \overline{VT}_{adults} }}{{\overline{VT}_{all} }} $$

**Table 1:** Viewing time difference and peak scores and the conceptual background of their adjustment

Score	Equation	Adjustment
*VT difference scores* The mean VT of the postpubescent adult categories is subtracted from the mean VT of the (pre)pubescent child categories. The table displays the different standardizations of the difference score		
D	$D = \overline{VT}_{children} - \overline{VT}_{adults}$	Simple difference index
D_x̅_	$D_{{\overline{X} }} = \frac{{\overline{VT}_{children} - \overline{VT}_{adults} }}{{\overline{VT}_{all} }}$	Difference index standardized by the mean VT of all categories to reduce the influence of the general reaction time
D_SD_	$D_{SD} = \frac{{\overline{VT}_{children} - \overline{VT}_{adults} }}{{SD_{all} }}$	Difference index standardized by the VT’s *SD* of all categories to reduce the influence of outliers
maxD	${\mathrm{maxD}} = {\mathrm{max}}\overline{VT}_{children} - \max \overline{VT}_{adults}$	Maximum difference index only using the child and adult categories with the highest mean VT to control for the preferred gender and developmental stage
D_lower 75%_	$D = \overline{VT}_{children} - \overline{VT}_{adults}$	Simple difference index only using the lower 75% of the VTs (i.e., excluding the highest VT) to reduce the influence of outliers
D_lower 50%_	$D = \overline{VT}_{children} - \overline{VT}_{adults}$	Simple difference index only using the lower 50% of the VTs (i.e., excluding the two highest VTs) to reduce the influence of outliers
D_middle 50%_	$D = \overline{VT}_{children} - \overline{VT}_{adults}$	Simple difference index only using the middle 50% of the VTs (i.e., excluding the lowest and highest VT) to reduce the influence of outliers

Score	*Total 100%*	*Lower 75%*	*Lower 50%*	*Middle 50%*	Adjustment
*Peak scores* The peaks of the VT profile in the child and adult categories are entered into a score. A peak in the listed category leads to a change of the score in the way presented on the right (e.g., for *P*_*1*_ a peak in the girl category in the *total 100%* cluster leads to an increase in the score by 2 points)					
*P*_1_	Girls + 2 Boys + 2 Adults – 1		Girls + 1 Boys + 1 Adults – 0.5		
*P*_2_	Girls + 2 Boys + 3 Adults – 1		Girls + 1 Boys + 1.5 Adults – 0.5		Interest in boys as a possibly superior predictor for pedophilia
*P*_3_	Girls + 1 Boys + 1 Adults – 0.5	Girls + 1 Boys + 1 Adults – 0.5	Girls + 1 Boys + 1 Adults – 0.5		Adding the *lower 75%* for more information on consistency
*P*_4_	Girls + 1 Boys + 1 Adults – 0.5	Girls + 1 Boys + 1 Adults – 0.5	Girls + 1 Boys + 1 Adults – 0.5	Girls + 1 Boys + 1 Adults – 0.5	Adding the *middle 50%* for more information on consistency
*P*_5_	Girls + 1 Boys + 1 Adults – 0.5	Girls + 2 Boys + 2 Adults – 1	Girls + 2 Boys + 2 Adults – 1	Girls + 1 Boys + 1 Adults – 0.5	Higher weighting of the *lower 75%* and *50%* because they are less vulnerable to outliers
RCP		Child + 1	Child + 1		Absolute score without weighting of adult peaks

The adjustment previously proposed by Schmidt et al. (2017) of only using the child and adult categories with the highest mean VT resulted in a *maximum difference index (maxD)*, see Eq. 4. This scoring method controls for the preferred gender as well as preferences for a specific developmental stage (e.g., Tanner 2). As a result, these preferences are not biased by lower VTs for non-preferred gender or sexual maturity stages.

4 $$ {{maxD}} = {{max}}\;\overline{VT}_{children} - \overline{VT}_{adults} $$

Previous use of the EISIP in single-case assessment has shown that certain stimuli produce higher VTs than the other stimuli of their category. For example, the first picture of a child presented may momentarily startle the participants. These single stimuli can increase the mean VT of a category substantially and misleadingly indicate increased sexual interests where none exist. The influence of this inconsistency within categories was investigated by integrating a measure of variance, the *standard deviation* (*SD*), in Eq. 5. Since a high *SD* represents less consistency, a mean VT with a high *SD* relies more likely on outlier stimuli in comparison to a mean VT with a low *SD*.

5 $$ D_{SD} = \frac{{\overline{VT}_{children} - \overline{VT}_{adults} }}{{SD_{all} }} $$

#### Viewing Time Clusters

To control for this inconsistency within categories, an updated version of the EISIP single-case analysis tool also calculates the absolute VTs without the two highest VTs in each category (i.e., only with the lower 75%) and without the four highest VTs (i.e., the lower 50%). An example of this profile is displayed in Fig. 1. This practice of observing different VT clusters was replicated here to evaluate the experiences with single cases in a bigger sample. Hence, the difference index *D* was also calculated for the VT clusters *lower 75%* (i.e., the three lower VTs per category), *lower 50%* (i.e., omitting the two highest VTs per category) and *middle 50%* (i.e., excluding the highest and lowest VT per category). The VT cluster with all four pictures per category was called the *total 100%*.

#### Absolute Profile Analysis

In single-case assessment, the results of the EISIP are usually analyzed by producing a graphic profile and visually interpreting the peaks and lows (Fig. 1). To mimic this, a peak analysis was conducted which mechanisms were inspired by the analyses of time series data (Ochi, 2019). To this end, a VT category was classified as a peak if its value was higher than the value of the preceding and following Tanner stage in its target sex category (e.g., mean VT of the male Tanner 2 stage was higher than the mean VT of male Tanner 1 and 3). Moreover—to explore how distinct the peak must be in order to be diagnostic—in a more *conservative* approach (in following formulas, tables, and graphs shortened to *cons.*), it had to differ from the mean of all VTs but its own by more than one *SD* (Eq. 6).

6 $$ peak_{x} = 1\;if\;\overline{VT}_{x} > \overline{VT}_{x - 1} \;and\;\overline{VT}_{x} > \overline{VT}_{x + 1} \, and \, \overline{VT}_{x} > (\overline{VT}_{all\;except\;x} + SD_{all\;except\;x} ) $$

In a second, more *progressive* classification (in following formulas, tables, and graphs shortened to *prog.)*, the VT category only had to differ from the mean of all other VTs by half a *SD* to be classified as a peak (Eq. 7).

7 $$ peak_{x} = 1\;if\;\overline{VT}_{x} > \overline{VT}_{x - 1} \;and\;\overline{VT}_{x} > \overline{VT}_{x + 1} \;and\;\overline{VT}_{x} > (\overline{VT}_{all\;except\;x} + \frac{1}{2}SD_{all\;except\;x} ) $$

These peak classifications were carried out in all four VT clusters.

#### Peak Scores

Different ways of transferring these absolute numbers of peaks into scores were explored. All of them were calculated based on different VT clusters. The idea of this concept was that a peak is more robust and less likely to rely on outliers if it remains a peak continuously across several VT clusters. The scoring algorithms were adjusted to explore the influence of different aspects. An overview of these adjustments is presented in Table 1.

First, only the *total 100%* and *lower 50%* were included with peaks in child categories raising the score and peaks in the adult category lowering it. Peaks in the adult category decreased the score only by half a unit, so a peak in a child category could not be fully compensated with an adult peak. This was done to explore a score focusing on an absolute interest in children rather than a preference. While in the first score *P*_*1*_ peaks in the girl and boy category weight the same, in a first adjustment the boy peaks were weight higher resulting in *P*_*2*_. This was explored due to the findings that men who have pedophilia and sexually offended against children have more male victims than other men who have been convicted for such offenses (Freund & Watson, 1991).

The next alteration of the scoring was the inclusion of the *lower 75%* (*P*_*3*_) and *middle 50%* (*P*_*4*_) cluster. In *P*_*5*_, the *lower 75%* and *50%* were weighed higher than the *total 100%* and the *middle 50%* because the former should be less vulnerable to high outliers than the latter. Lastly, to make the score even more absolute (i.e., only reflecting the sexual interest in children independently from the sexual interest in adults), the *robust child peak* (*RCP*) was calculated based on the information if there was at least one child peak in the *lower 75%* and *lower 50%* VT cluster. This score represents the basic assessment question whether there is any peak in a child category and if it is consistent.

More scores than presented here were explored during the analyses, e.g., different standardizations of the difference score and adjusted weightings of the VT clusters. These scores are not described in further detail here because they performed similarly or worse in the validity analyses and did not yield additional insights into the mechanisms of the VT method. For a more detailed description of the excluded scores, see Table S1 in the online supplementary material. Additionally, all scores were calculated using the *z*-score normalized VTs. However, this type of standardization did not result in higher validity as well.

#### Validity Criteria and Statistical Analyses

Before conducting any analyses, VTs above 20,000 ms were set as missing values (0.37% of trials) and latencies between 10,000 and 20,000 ms were truncated to 10,000 ms (1.38% of trials) in order to reduce the unduly influence of extreme outliers which are a common source of nuisance in online studies due to the lack of control over the testing setting. No suspiciously short VTs (below 300 ms) were observed.

Different validity criteria were calculated based on self-report. The sexual attractiveness rating in the VT task was an especially interesting criterion because it was assessed concurrently with the corresponding VTs, so controlled (i.e., self-reported) and automatic (i.e., response latency) reaction to the same stimuli could be compared. To validate the absolute VT scores, they were correlated with the mean rating of each of the categories and the mean answers to the four ESIQ target categories.

For the validation of the relative scores, maximum difference indices of the rating (*maxD*_*rating*_) and the ESIQ (*maxD*_*ESIQ*_) were formed. Furthermore, several dichotomous indices were used (1 = criterion has been fulfilled vs. 0 = criterion has not been fulfilled) indicating *positive ratings* (at least one picture of a child was rated 2 or more), *any behavior* (any sexually abusive behavior involving children has been indicated), *contact behavior* (contact child sexual abusive behavior has been indicated), *CSAM use* (CSAM use has been indicated), *fantasy* (any kind of sexual fantasy involving children has been indicated), and *any interest* (any behavior or fantasy have been indicated). All validity criteria reached acceptable up to excellent odd–even internal consistencies (Table 2). Furthermore, Table 2 presents the correlations among the validity criteria, which were uniformly positive, suggesting the presence of an underlying latent factor. Only a few people of the large sample admitted sexual interest in children in the self-report questions which led to low base rates of the validity criteria. Thus, the validity of the relative scoring methods was gauged using receiver operating characteristic (ROC) analyses which are more robust against influences of low base rates (Babchishin & Helmus, 2016).^1^

**Table 2:** Correlations and reliability (Cronbach’s α) of the validity criteria

		1	2	3	4	5	6	7	8	α
1	MaxD_rating_	–								0.90
2	MaxD_ESIQ_	0.30^**^	–							0.87
3	Positive rating	0.11^**^	0.15^**^	–						0.84
4	Any behavior	0.14^**^	0.31^**^	0.23^**^	–					0.54
5	Contact behavior	0.12^**^	0.33^**^	0.15^**^	0.68^**^	–				0.75
6	CSAM use	0.12^**^	0.24^**^	0.20^**^	0.85^**^	0.34^**^	–			n/a
7	Any interest	0.15^**^	0.35^**^	0.32^**^	0.75^**^	0.51^**^	0.64^**^	–		0.74
8	Fantasy	0.16^**^	0.38^**^	0.31^**^	0.46^**^	0.37^**^	0.41^**^	0.85^**^	–	0.79

** *p* < 0.01. n/a, not applicable; Validity criteria: *maxD*_*rating*_, maximum difference index of the stimulus rating in the VT task; *maxD* maximum difference index of the self-report measure ESIQ; *positive rating*, at least one picture of a child was rated 2 or more; *any behavior*, any sexually abusive behavior involving children has been indicated; *contact behavior*, contact child sexual abusive behavior has been indicated; *CSAM use*, CSAM use has been indicated; *fantasy*, any kind of sexual fantasy involving children has been indicated; *any interest*, any behavior or fantasy have been indicated

Hierarchical logistic regressions were carried out to test for incremental validity of the new scoring algorithms. Moreover, the correlation of the scorings with other factors (general processing speed, test sequence, age, occupation, education) that could possibly influence the VTs was conducted. To assess their reliability, relative and peak scores were calculated separately for the odd and even halves of the stimuli in each category. Internal consistencies (Cronbach’s α) were then computed across all stimuli or items for absolute scores and across the odd and even scores for relative scores. Lastly, the number of men who would be categorized as sexually interested in children based on the validity criteria as well as the scoring algorithms with the best psychometric properties was calculated and compared. To gain an estimation of the accuracy of the results, most of the correlations and frequency analyses were bootstrapped with *B* = 1500 samples to test on an α-level of 0.01 (Davidson & MacKinnon, 2000).

## Results

All following comparisons of the VT scores regarding effect sizes are descriptive. Instead of testing hypotheses, the psychometric properties of the scoring algorithms were descriptively explored. The bootstrapping showed that the calculated associations were mostly accurate with the standard error falling between 0.01 and 0.04 and the 95% confidence interval ranging between 0.01 and 0.09. The magnitude of correlations is interpreted following the classification of J. Cohen (2013), the ROC analyses according to Babchishin and Helmus (2016).

### Absolute Scoring

All correlations and internal consistencies are available in the online supplementary material (Tables S2 and S3).

#### Validity

The absolute scoring of the VTs reached negative up to moderate positive correlations with the corresponding attractiveness rating categories (− 0.03 ≤ *r* ≤ 0.36; Table S2). However, a clear pattern could be observed with the male adult categories having consistently higher correlations (*r*s > 0.22) than the male child categories (*r*s >− 0.03) and the female categories falling in-between (girls *r*s > 0.09, women *r*s > 0.18). This was more pronounced when correlating the absolute VT scoring with the ESIQ categories (women *r*s > 0.29, men *r*s > 0.27, girls *r*s > 0.07, boys *r* > 0.01). On average, the absolute scores calculated with the clusters *total 100%* and *middle 50%* performed the best (Table S2).

The absolute number of peaks in each of the ten Tanner stage target sex categories reached overall higher correlations with the respective rating categories than the absolute scores (Table S2). The same pattern was observed of male adult groups yielding the highest correlations (*r*s > 0.30), closely followed by most female Tanner stages (*r*s > 0.14) and the male child categories again revealing the lowest correlations (*r*s > 0.10). Female Tanner 5 surprisingly only reached negative or non-significant correlations. The correlations with the ESIQ resulted in a similar pattern as the absolute VT scores, with the mean EISIP score for men showing the highest correlations (*r*s > 0.30), followed by women (*r*s > 0.14), and children (*r*s > 0.05). Overall, the *lower 75%* and the conservative peak analyses performed better than the progressive approach and the other VT clusters (Table S2).

#### Reliability

Regarding the reliability, the absolute scoring of all ten categories had acceptable internal consistencies (.73 ≤ α ≤ 0.80; Table S3) in the *total 100%* cluster but performed good up to excellent in all other VT clusters (0.87 ≤ α ≤ 0.95). Contrary to the correlations with the validity indices, the adult categories did not exhibit higher values than the child categories. The reliability of the absolute number of peaks in the *total 100%* cluster was substantially worse (0.04 ≤ α ≤ 0.30) than in the other clusters (0.22 ≤ α ≤ 0.80). The lower 75% cluster performed by far the best (0.54 ≤ α ≤ 0.80). There was no clear advantage of the conservative or progressive approach (Table S3).

### Relative Scoring

#### Validity

The results from the ROC analysis are presented in Table 3. The typically used difference index *D* and the maximum difference index *maxD* which only uses the child and adult categories with the highest mean category VTs both produced large areas under the curve (AUCs) between 0.74 and 0.93. The standardization of *D* with the mean of all VTs *D*_*x̅*_ led to a marginal enhancement of the AUCs (0.76 ≤ AUC ≤ 0.93). Using the *SD* instead of the mean descriptively produced even slightly higher effect sizes with *D*_*SD*_ being the best predictor of almost all validity indices (0.78 ≤ AUC ≤ 0.94). The difference scores in the three VT clusters *lower 75%*, *lower 50%* and *middle 50%* did not produce substantially higher effect sizes compared to *D* conducted in the *total 100%* cluster (0.70 ≤ AUC ≤ 0.92).

**Table 3:**
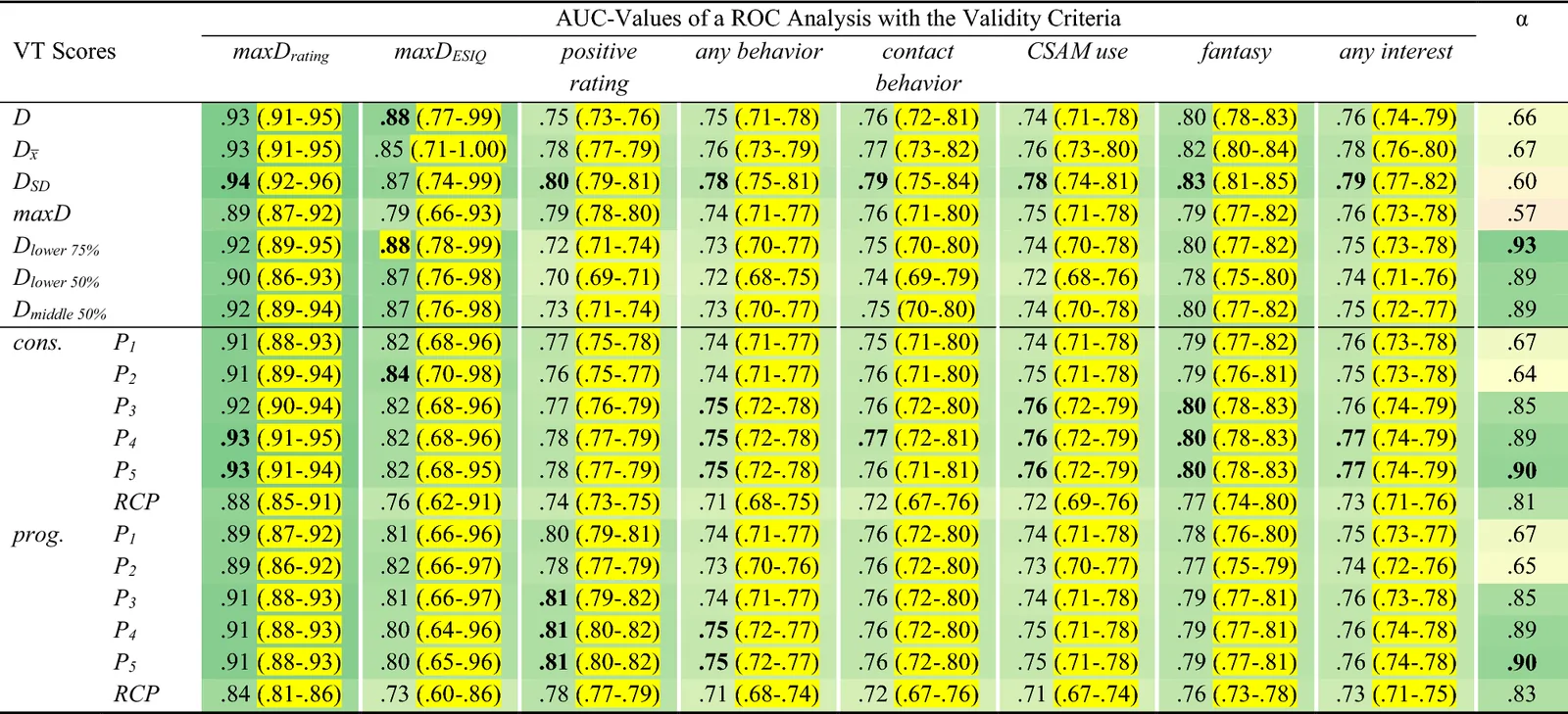
Validity and reliability of the VT difference and peak scores: ROC analysis with the validity criteria; internal consistency (Cronbach’s α)

*CI*_*95%*_ of AUC values in brackets. Best predictor of each validity criterium across all relative scores or across all peak scores is formatted bold. VT, viewing time; ROC, receiver operating characteristic analyses; AUC, area under the curve. Validity criteria: *maxD*_*rating*_ = maximum difference index of the stimulus rating in the VT task; *maxD*_*ESIQ*_, maximum difference index of the self-report measure ESIQ (both difference indices were dichotomized for the ROC analysis with scores > 0 being coded as 1 and scores ≤ 0 being coded as 0); *positive rating*, at least one picture of a child was rated 2 or more; *any behavior*, any sexually abusive behavior involving children has been indicated; *contact behavior*,  contact child sexual abusive behavior has been indicated; *CSAM us, * CSAM use has been indicated; *fantasy*, any kind of sexual fantasy involving children has been indicated; *any interest* , any behavior, or fantasy has been indicated. VT difference scores: *D*, simple difference index subtracting the mean VT for adult stimuli from the mean VT for child stimuli; *D*_*x̅*_, *D* divided by the mean of all VTs; *D*_*SD*_, *D* divided by the SD of all VTs; *maxD*, difference index using the maximum child and adult categories; *D*_*lower 75%*_, *D* calculated with the mean of the three lowest VTs per category; *D*_*lower 50%*_, *D* calculated with the mean of the two lowest VTs per category; *D*_*middle 50%*_, *D* calculated with the mean excluding the highest and lowest VT per category. VT peak scores: cons., conservative approach in which the peak had to differ from the mean of all other VTs by more than one SD; prog., progressive approach in which the peak had to differ from the mean of all other VTs by more than half a SD; *P*_*1*_, positive points were given for peaks in child categories in the *total 100%* or *lower 50%* cluster and negative points for peak in adult categories; *P*_*2*_, peaks in boy categories were weight higher than peaks in girl categories; *P*_*3*_, used the *total 100%*, *lower 75%* and *lower 50%* cluster; *P*_*4*_, used the *total 100%*, *lower 75%, lower 50%* and *middle 50%* cluster; *P*_*5*_, peaks in the *lower 75%* and *lower 50%* cluster were weight higher than peaks in the other clusters; RCP, absolute score only taking the child categories into account

Overall, the peak scores did not perform better than the difference scores regarding validity. *P*_*1*_ and *P*_*2*_ produced similar AUCs, showing that the higher weighting of the boy categories did not make a difference (0.73 ≤ AUC ≤ 0.91). Across almost all validity criteria, *P*_*3*_ and *P*_*4*_ showed slightly higher AUCs than *P*_*1*_ and *P*_*2*_ (0.78 ≤ AUC ≤ 0.94). This suggests that the addition of information from more VT clusters offered only a minor improvement. Increasing the weighting of the VT clusters *lower 75%* and *lower 50%* in the score *P*_*5*_ did not influence the validity effect sizes (0.71 ≤ AUC ≤ 0.93). The score *RCP* (taking only the peaks in child categories into account) performed the worst of all peak scores (0.71 ≤ AUC ≤ 0.88). The conservative approach had a slight advantage compared to the progressive approach.

#### Incremental Validity

Results of the logistic regressions are shown in Table 4. The score *maxD* was added into the regression in a first step because it is the current standard. In a second step, each of the most promising alternative scoring algorithms—*D*_*SD*_,* D*_*lower 75*%_, and *P*_*5*_ (both *conservative* and *progressive*)—was added in distinct analyses. The odds ratio of the *maxD* score did not differ significantly from 1, indicating that it did not significantly contribute to the prediction of the two criterion variables *any behavior* and *fantasy*. However, when added to the model in the second step, the three algorithms *D*_*SD*_, *P*_*5*_ (*conservative*) and *P*_*5*_ (*progressive*) demonstrated incremental validity, with odds ratios significantly differing from 1. The *D*_*lower 75*%_ score did not add substantial incremental variance to the model.

**Table 4:** Overview of logistic regressions for the VT scoring algorithms predicting sexual behavior and fantasies involving children and the incremental validity of the conceptually more sophisticated scoring algorithms

Predictor	Any behavior				Fantasy			
	∆*R*^*2*^	*B*	*SE*	*OR [CI*_*95%]*_	*∆R*^*2*^	*B*	*SE*	*OR [CI*_*95%*_*]*
Step 1	0.099				0.156			
* constant*		− 2.819	0.065	0.060		− 2.501	0.058	0.082
* maxD*		0.001	< 0.001	1.001 [1.001, 1.001]		0.001	< 0 .001	1.001 [1.001, 1.001]
Step 2	0.041				0.050			
Model A								
* constant*		− 2.287	0.078	0.102		− 1.927	0.070	0.146
* maxD*		< 0.001	<0.001	1.000 [1.000, 1.000]		< 0.001	< 0.001	1.000 [1.000, 1.000]
* D*_*SD*_		2.464	0.264	11.748 [6.996, 19.726]		2.719	0.245	15.169 [9.380, 24.531]
Model B	0.007				0.016			
* constant*		− 2.58	0.08	0.08		− 2.162	0.075	0.115
* maxD*		< 0.001	< 0.001	1.000 [1.000, 1.001]		< 0.001	< 0.001	1.000 [1.000, 1.001]
* D*_*lower 75%*_		0.001	< 0.001	1.001 [1.00, 1.001]		0.001	< 0.001	1.001 [1.001, 1.002]
Model C	0.021				0.029			
* constant*		-2.972	0.073	0.051		− 2.688	0.066	0.068
* maxD*		< 0.001	< 0.001	1.000 [1.000, 1.000]		< 0.001	< 0.001	1.000 [1.000, 1.001]
* P*_*5*_* (cons.)*		0.204	0.030	1.226 [1.157, 1.299]		0.234	0.027	1.264 [1.199, 1.332]
Model D	0.023				0.029			
* constant*		− 3.139	0.085	0.043		− 2.860	0.077	0.057
* maxD*		< 0.001	< 0.001	1.000 [1.000, 1.000]		< 0.001	< 0.001	1.000 [1.000, 1.001]
* P*_*5*_* (prog.)*		0.202	0.028	1.224 [1.159, 1.292]		0.222	0.025	1.248 [1.188, 1.311]

*∆R*^*2*^ is Nagelkerke’s R2. Step 1 is the same for every tested model; step 2 reports different tests of incremental validity. VT, viewing time. Validity criteria: any behavior, any sexually abusive behavior involving children has been indicated; fantasy,  any kind of sexual fantasy involving children has been indicated. VT scores: maxD,  difference index using the maximum child and adult categories; DSD, the simple difference index D divided by the SD of all VTs; Dlower 75%, D calculated with the mean of the three lowest VTs per category; P5, peak score with positive points given for peaks in child categories and negative points for peaks in adult categories, peaks in the lower 75% and lower 50% cluster were weight higher than peaks in the other clusters; cons., conservative approach in which the peak had to differ from the mean of all other VTs by more than one SD; prog., progressive approach in which the peak had to differ from the mean of all other VTs by more than half a SD

#### Reliability

The VT difference scores in the cluster *total 100%* showed poor or questionable odd–even internal consistencies (0.57 ≤ α ≤ 0.67) with *D*_*x̅*_ performing the best out of the four. In contrast, the three scores *D*_*lower 75%*_, *D*_*lower 50%*_ and *D*_*middle 50%*_ produced good up to excellent internal consistencies (0.89 ≤ α ≤ 0.93). *D*_*lower 75%*_ had the highest reliability of all scores (Table 3).

When comparing the peak scores, integrating more VT clusters into the score showed a larger positive effect on reliability than on validity. The scores *P*_*1*_ and *P*_*2*_ (which include information from the *total 100%* and *lower 50%* cluster) only produced questionable reliabilities between α = 0.64 and α = 0.67. Contrary, all other peak scores (*P*_*3*_*, P*_*4*_*, P*_*5*_*,* and *RCP*) had good internal consistencies (αs > 0.81). The score *P*_*5*_, which builds on all four VT clusters and weights the *lower 75%* and *lower 50%* higher than the other two, reached an excellent α = 0.90. There was no clear advantage of the progressive or conservative peak analysis in terms of reliability (Table 3).

#### Influence of Confounding Variables

All correlations with confounding variables can be accessed in the online supplementary material (Table S4). Since the direction of the correlation (negative or positive) was not of importance regarding the influence of other factors, the absolute values were compared. All difference scores except for $D_{\bar{x}}$ correlated significantly with the general reaction time (mean VT across all categories). The normal difference index *D* performed the worst in all three VT clusters with significant correlations above |*r*|= 0.25. As to be expected, the standardization of the difference score with the mean of the VTs (*D*_*x̅*_) or the SD (*D*_*SD*_) led to lower (|*r*s|≤ 0.04) or even non-significant correlations with the mean VT. The peak scores exhibited low correlations in a similar range (|*r*s|≤ 0.10).

A test-immanent factor was the presentation order of the different tests. Test order correlations ranged from non-significant to close to zero (|*r*s|≤ 0.03), so that a clinically meaningful influence on the VT task can be ruled out. Similar results were observed regarding demographic participant variables with age having no significant correlation with any VT score and the others (occupation and education) only displaying non-substantial correlations (|*r*s|≤ 0.06).

#### Estimates of Sexual Interest in Children

**Fig. 2:**
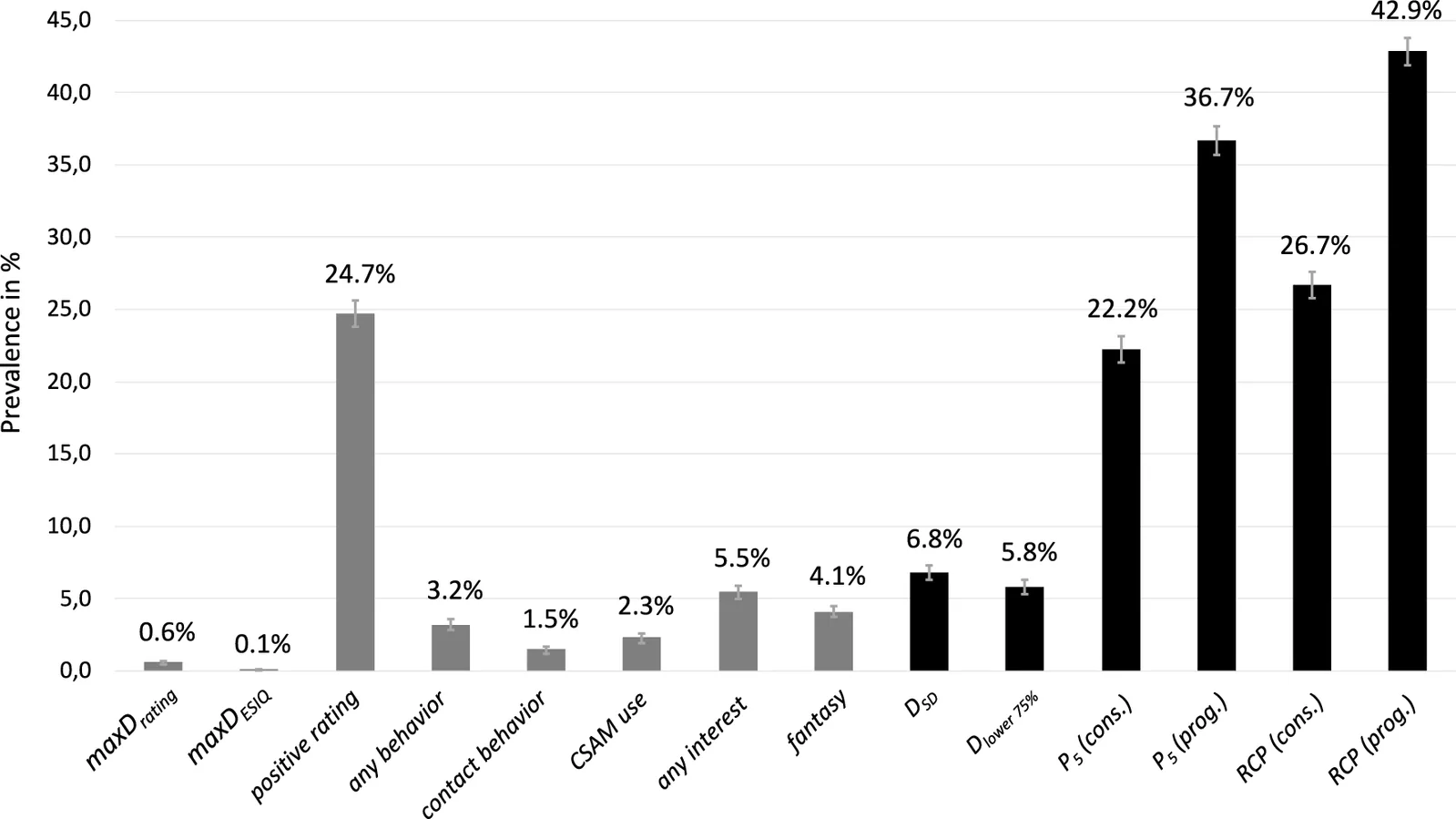
Prevalence of participants (Percentage ± 95% CI) being classified as sexually interested in children according to the index. Note. maxDrating = maximum difference index of the stimulus rating in the VT task; maxDESIQ = maximum difference index of the self-report measure ESIQ (both difference indices were dichotomized for the ROC-analysis with scores > 0 being coded as 1 and scores ≤ 0 being coded as 0); positive rating = at least one picture of a child was rated 2 or more; any behavior = any sexually abusive behavior involving children has been indicated; contact behavior = contact child sexual abusive behavior has been indicated; CSAM use = CSAM use has been indicated; fantasy = any kind of sexual fantasy involving children has been indicated; any interest = any behavior or fantasy has been indicated; DSD = D divided by the SD of all VTs; Dlower 75% = D calculated with the mean of the three lowest VTs per category; P5 = peak score with positive points given for peaks in child categories and negative points for peaks in adult categories, peaks in the lower 75% and lower 50% cluster were weight higher than peaks in the other clusters; RCP = absolute score only taking the child categories into account; cons. = conservative approach in which the peak had to differ from the mean of all other VTs by more than one SD; prog. = progressive approach in which the peak had to differ from the mean of all other VTs by more than half a SD

The percentage of men in the sample who would be categorized as exhibiting increased sexual interest in children was calculated for all self-report-based criteria as well as the selected VT scoring algorithms (Fig. 2). The rating index *maxD*_*rating*_ classified 0.6% (CI_95%_[0.5%, 0.8%]) of the sample as sexually preferring children while the ESIQ index *maxD*_*ESIQ*_ produced a much lower rate of 0.1% (CI_95%_[0.1%, 0.2%]). Of all participants, 3.2% (CI_95%_[2.8%, 3.6%]) indicated sexual behavior involving children, with 1.5% (CI_95%_[1.3%, 1.8%]) reporting direct sexual contact and 2.3% (CI_95%_[2.0%, 2.7%]) CSAM use. The combination of the latter two was indicated by 0.7% (CI_95%_[0.5%, 0.8%]). A sexual fantasy involving children was reported by 4.1% (CI_95%_[3.7%, 4.5%]) of the sample and the combined sexual interest in children as operationalized by fantasies or behavior involved 5.5% (CI_95%_[5.1%, 6.0%]).

Notably, the highest self-report-based estimate in this study with 24.7% (CI_95%_[23.8%, 25.6%]) was the number of people that rated at least one picture of a child as somewhat sexually attractive (i.e., 2 or more). A similar range of 22.2% (CI_95%_[21.3%, 23.1%]) was classified as having an absolute sexual interest in children according to the conservative peak score *P*_*5*_ of the VTs. The progressive approach even led to 36.7% (CI_95%_[35.7%, 37.7%]) being categorized. Due to the non-overlapping confidence intervals, these differences were statistically significant. A similar pattern could be observed regarding the peak score *RCP* with the progressive approach categorizing 42.9% (CI_95%_[41.8%, 43.9%]) as having an increased sexual interest in children while the conservative only classified 26.7% (CI_95%_[25.8%, 27.6%]). Again, confidence intervals did not overlap. The difference indexes *D*_*lower 75%*_ and *D*_*SD*_ suggested 5.8% (CI_95%_[5.3%, 6.3%]) and 6.8% (CI_95%_[6.3%, 7.3%]) sexually preferring children over adults, respectively.

## Discussion

Sexual interest in children is a highly relevant risk factor for sexual (re)offending against children (Seto et al., 2023). Because self-report is often not reliable in clinical and forensic contexts, other ways to measure sexual interest in children have been researched in the past decades (Schmidt & Banse, 2022). A prominent example are VT measures, which reflect the time a person spends deciding about the subjective sexual attractiveness of pictures of different target groups (e.g., adults and children; Imhoff et al., 2010). In this study, several new scoring algorithms of the VT measure with better psychometric properties than the standard difference index have been identified. Their validity, reliability, and dependence on confounding variables have been explored in an extensive male German community sample (taken from Dombert et al., 2016). Additionally, the number of men in the sample classified as having increased sexual interest in children based on the different VT scores and self-report was determined.

### Reliability and Validity of the Scoring Algorithms

Overall, the convergent validity (Pedneault et al., 2021; Schmidt et al., 2017) and reliability (Schmidt et al., 2014; Welsch et al., 2021) of the use of the VT effect for the assessment of sexual interest in children and adults have been replicated among community men, adding more compelling evidence that the VT measure even works with only four stimuli per category (Jahnke et al., 2022 for more preliminary evidence). The reliability of the absolute VT scores of the ten Tanner stage × target sex categories was slightly lower than the ones reported for the EISIP before (Banse et al., 2010; Schmidt et al., 2014); however, it was still acceptable. The decline in reliability is concomitant with the reduction in the number of presented stimuli.

All VT difference scores produced similarly high effect sizes in the ROC analyses when linking them to self-reported indicators of sexual interest in children. This shows that, overall, the VT sexual interest measure’s validity is quite robust and not greatly affected by changes to its scoring. When going into detail, the optimal scoring algorithm regarding validity was *D*_*SD*_ which standardizes the classic difference score of child and adult VTs by the standard deviation. The peak scores also performed well; however, they did not reach the effect sizes of *D*_*SD*_. Both the *D*_*SD*_ and the peak score *P*_*5*_ had incremental validity above the current standard difference score *maxD*. The poor performance of the difference scores *maxD* and *D*_*lower 75%*_ in the logistic regressions does not indicate insufficient validity because it can be explained by the low base rates of relevant behavior and fantasies in this community sample. Base rates have a larger impact on effect sizes of correlations (Babchishin & Helmus, 2016) and logistic regressions (Sharma et al., 2011) than on ROC analyses; therefore, the bivariate AUCs ought to be seen as the main indicator of validity in this study.

The effect of using different scoring algorithms was much more pronounced for the reliability than for the validity. While the difference scores conducted with all four VTs of each category exhibited questionable internal consistencies at best, the scores conducted in the other clusters *lower 75%*, *lower 50%*, and *middle 50%* which exclude the more extreme VTs all performed well. The most reliable score was *D*_*lower 75%*_. In parallel, the main reliability difference in the adjustments of the peak scores was made by incorporating the information of more VT clusters into the score. This supports the recently introduced practice in EISIP single-case assessments of only counting a peak as relevant if it appears across all VT clusters (i.e., peak robustness check).

Contrary to the findings of Schmidt et al. (2014), the general processing speed was substantially associated with the unstandardized difference index in this sample (see Table S4). Independence from the processing speed ensures a lower association of the final scores with participant characteristics indicative of cognitive abilities such as intelligence, age, and familiarization with computers. Notably, Schmidt et al. (2014) correlated the VT score with the general processing speed in *unrelated cognitive classification tasks* (i.e., subtests in Implicit Association Tests) while this study used the general processing speed in the VT task. Thus, the higher correlations in this study are probably (at least in part) due to shared method variance of the VT scores and the general VT processing speed (which is controlled for in case of *D*_*x̅*_*)*. The non-substantial correlations with other potentially confounding variables such as age and education support the conclusion that all explored scores are relatively independent from demographic and/or cognitive characteristics.

Regarding the adjustments of the peak scores, some interesting observations were made: While the incorporation of more VT clusters made substantial differences in validity and reliability, other information such as—notably—whether the child peak is in a male or female child target category did not add incremental value. The differences between the progressive and conservative peak analysis were also small. The *RCP* which did not take adult peaks into account produced slightly lower validity and reliability rates. This is probably (at least in part) due to the integration of fewer diagnostically valid information and reflected in recent findings that sexual interests in adults are crucial in differentiating between men with and without pedophilic tendencies who have sexually abused children (Schippers et al., 2023).

Overall, almost all scores performed at least satisfactory or better, supporting the VT measure’s validity and reliability independently from its scoring. Since no clear overall “winner” could be found, users should base the decision for a scoring algorithm on one’s research or diagnostic priorities. *D*_*lower 75%*_ can be chosen as the best difference index when one prioritizes reliability over validity, and *D*_*SD*_ when one gives priority to high validity and independence of confounding variables. When using a peak score, the progressive analysis is the better pick for a more sensitive result (i.e., tolerating more false positives) and the conservative approach when prioritizing specificity (i.e., tolerating more false negatives). Out of all peak scores, *P*_*5*_ can be highlighted as the best peak score we explored. However, the advantage of *P*_*5*_ over the scores *P*_*3*_ and *P*_*4*_ is only small. If one gives priority to efficiency, *P*_*3*_ might be the better choice since it performs almost as well and relies on less VT clusters.

### The Dependence of Prevalence Estimations on the Measurement and Scoring Approach

The data in the present research were based on Dombert et al. (2016). Despite a slightly different effective sample than used in the original study and adding a bootstrapping approach, the original proportion of participants with an absolute interest in children based on the self-report measures were replicated exactly with 4.1% of the sample reporting sexual fantasies involving children. Out of these, less than half (1.8% of the whole sample) admitted CSAM use or some form of sexual abuse or harassment of children and 1.4% of all participants reported a behavior without having corresponding fantasies. This further supports prior concordance findings that while not all men with pedophilic interests act according to their sexual interests and not all men who sexually abused children exhibit pedophilic interests (Joyal & Carpentier, 2022; Seto et al., 2021), there is still a substantial association of sexual fantasies with child abusive behavior.

Regarding the proportion of sexual interest in children suggested by the different measures used in this study, one needs to differentiate between a preference for children and an absolute interest. The self-reported sexual preference for children is reflected in the difference indices of the ESIQ and attractiveness ratings suggesting a percentage of 0.2 and 0.8%, respectively. The difference between these two estimates can be explained by the ESIQ asking explicitly about past (lifetime as an adult) sexual fantasies and behavior as opposed to mere sexual attraction of the target stimuli in the attractiveness ratings. The former should bear much more moral/criminal implications and thus are likely to be much more amenable to social desirability.

The sample proportion of a sexual preference for children suggested by the VT difference indices was higher, lying between 5.8 and 6.8%. This supports the idea that the VT effect may help to identify people that sexually prefer children but do not admit it to themselves or others (or it may simply be more sensitive in a psychometric sense). And while these numbers differ from the ones based on self-report, they still fall into the range of former reported prevalence estimations between 2.1 and 24.0% (Savoie et al., 2021).

The high number of 25% who rated at least one picture of a child as somewhat sexually attractive is notable as it was almost five times higher than the percentage suggested by the index of self-reported fantasies or behavior. Between 22 and 27% of the participants were also classified as having an absolute sexual interest in children according to the conservative peak scores. These numbers fit the 25% of participants that experienced physiological sexual arousal toward children in the only other study to date using measures other than self-report (Hall et al., 1995). The progressive peak scores offer even higher estimates between 36.7 and 42.9%. Since these numbers are substantially surpassing most other reported prevalences, the condition of only differing from the mean VT by half a standard deviation is likely too sensitive. This also fits with the conservative scores performing slightly better in the validity and reliability analyses.

Overall, the differing proportions of the sample classified as sexually interested in children further support the conceptual background of the scoring algorithms: The more progressive approach leads to a higher prevalence than the conservative one, and the peak analysis suggests a higher prevalence of absolute sexual interest in children than the preference suggested by the difference score. This further underlines the notion that the best-fitting scoring algorithm should be chosen based on the goals of the intended analysis: whether one researches absolute interest or preference, whether one prioritizes validity or reliability, and whether one aims for specificity or sensitivity of a classifier.

Furthermore, the great disparity in the number of participants who are categorized as sexually interested in children in our sample demonstrates how dependent prevalence estimations are on (1) the conceptualization of sexual interest in children as an absolute interest versus a relative preference, (2) the measurement method, and (3) the scoring algorithm of the chosen measure. As we could find corresponding prevalence estimations in the literature for almost each percentage range suggested by different measures and scores in our sample, the results showcase that prevalence estimates of sexual interest in children should be handled with care and must always be considered in the context of sample composition, definition of sexual interest in children, and measurement method. Because prevalence estimates are not just deciding arguments in scientific but also political and public discourses, this should also be taken into account when communicating such research findings to clinicians, politicians, or the public.

### Strength and Weaknesses

The scoring of the VT task is currently not standardized or even extensively discussed as a relevant factor. This study represents an initial step in exploring the effects of different scoring approaches on validity, reliability, and the interpretation of the results (highlighted in the exemplary prevalence estimates). To our knowledge, this study is the first one to reveal the diverging proportion of participants who are categorized as exhibiting increased sexual interest in children when using self-report versus indirect measures. Implementing norms for the VT measure would be a tremendous help for clinicians and could be set as a final goal of this line of research. However, as representative samples and a gold standard criterion measure are usually necessary requirements for norm establishment, the question of feasibility needs to be discussed. Moreover, at present there is no systematic research on the potential impact of assessment context (i.e., research or forensic settings) on the norms generated from it.

Importantly, one should keep in mind that the overarching complication in sexual interest research is the lack of a perfectly valid empirically observable indicator of pedo(hebe)philia. Without such a “perfect” criterion, assessing convergent validity through self-report measures in an anonymous online sample offers one of the most precise approximations currently available. To further substantiate these findings, however, the psychometric properties of the proposed scoring algorithms should be replicated across diverse settings such as samples of, preferably, self-identified individuals with sexual interests in children (e.g., Jahnke et al., 2022) or persons who have been convicted for child sexual abusive behavior and assessed with PPG.

All ages, education, and occupation groups among German male adults were sufficiently represented in the sample and correlations of these attributes with the main variables were low or nonsignificant. However, the generalizability of our results to other (particularly non-WEIRD; Henrich et al., 2010) ethnic and cultural contexts is limited because race and ethnicity were not recorded in the original data (cf. Thompson & Hanson, 2025). Obviously, these results are not telling at all about female individuals for whom, however, this kind of research is empirically particularly challenging due the supposed low prevalence of female pedo(hebe)philia (Bártová et al., 2021).

### Implications

Currently, the scoring of the VT measure is not standardized across different research groups and fields of application. This exploration of different scoring approaches on one hand portrays the robustness of the VT task’s validity to small changes such as using a smaller number of stimuli or the standardization of the score. On the other hand, the results highlight that—as long as there is no standard scoring approach—the choice of scoring is crucial. Before conducting research or diagnostics using VT, one should reflect the goal of the measurement and decide on the scoring accordingly. Is reliability or validity the priority? Is sensitivity or specificity more important for decisions based on these results? Is it relevant for the diagnostics to include information on sexual interest in adults? How does the scoring fit the supposed underlying latent structure (i.e., categorical or dimensional) of sexual interests in question? The effects of the scoring become particularly striking in the exemplary estimations of prevalence in this study. Thus, the implications of our investigations should especially be taken into account when choosing the measurement and scoring approaches and interpreting the results in future prevalence estimation studies.

### Future Research Directions

As this work was an exploration of different scoring approaches of the VT task, it constitutes the first step in systematically investigating the scoring and interpretation of this indirect measure of sexual interest in children. In the future, the validity and reliability of the proposed scoring algorithms need to be confirmed in non-community samples such as people who have sexually offended against children or who self-identify as pedophilic. At the same time, one should consider whether assessments have been collected in anonymous research or in applied forensic settings where its outcomes may have severe ramifications for the assessed individuals. Additionally, other measures of sexual interest in children such as PPG should be included as additional validity indicators for triangulation purposes due to the lack of an objective gold standard for pedo(hebe)philic sexual interests.

## Supplementary Information

Below is the link to the electronic supplementary material.

Supplementary file1 (DOCX 163 KB)

## Data Availability

The data cannot be made available because participants were promised that their answers would not be published to guarantee their anonymity and minimize self-selection effects. The self-report questions and measures relevant for this study are described in the method section, for an at-length description of the EISIP see Banse et al. (2010). Please see www.eisip.org if you are interested in using the EISIP for your own work or research. The statistical syntax is available in the online supplemental material.

## Footnotes

1 Correlations between the scores and validity criteria were also calculated. They showed similar variation to the AUC values but smaller overall effect sizes which can be attributed to the low base rate.
