# Supplementary material for: Scoring Algorithms to Optimize Viewing Time Measures of Sexual Interest in Children
Source: Arch Sex Behav. 2026 Aug 8;55(6):2465–81. doi: 10.1007/s10508-026-03490-6 (PMC13597560; doi:10.1007/s10508-026-03490-6)
Supplement: Supplementary file 1 [file 10508_2026_3490_MOESM1_ESM.docx]

Supplemental Material of […] (submitted). Scoring Algorithms to Optimize Viewing Time Measures of Sexual Interest in Children.

**Table S1**

*Additionally Explored VT Scoring Algorithms*

| Scoring Algorithm | Adjustment |
| --- | --- |
| Difference Scores |  |
| ${maxD}_{SD}=\frac{{max\overline{VT}}_{children}-{max\overline{VT}}_{adults}}{{SD}_{{max\bar{VT}}_{children}, {max\bar{VT}}_{adults}}}$ | This score integrates two types of standardization (using only the maximum child and adult category as well as dividing by the SD). |
| $D_{SD2}=\frac{\overline{VT}_{children}}{{zSD}_{children}}-\frac{\overline{VT}_{adults}}{{zSD}_{adults}}$ | This score is a variation of the standardized difference score. Instead of dividing the difference score by the SD of all VTs, the VTs of each age category were divided separately by the SD of the z-score normalized VTs. |
| $D_{SD3}=\left( \overline{VT}_{children}*\left( 1-{zSD}_{children} \right) \right)- \left( \overline{VT}_{adults}*\left( 1-{zSD}_{adults} \right) \right)$ | This score tested whether a multiplicative instead of a divisional incorporation of the variance would be a better model. To keep the concept of a high score showing a reliable sexual interest in children vs. a low score showing no sexual interest in children, the SDs were subtracted of 1 before being multiplicated with the VTs. So, a high SD showing probable influence of outliers led to a low multiplicator leading to a lower score. |
| $D_{peaks}={peaks}_{children}-{peaks}_{adults}$ | Following the scoring of the absolute viewing times, a difference index for the number of peaks in the child and adult categories was calculated. |

| Peak Scores | | | | |  |
| --- | --- | --- | --- | --- | --- |
|  | Total 100% | Lower 75% | Lower 50% | Middle 50% |  |
| *P_A_* | girls + 2  boys + 2  adults – 1 |  | if the same peak is present in the total 100%:  girls + 1  boys + 1  if the other sex’s peak is present in the total 100%:  girls + 0.5  boys + 0.5  adults – 0.5 |  | With this score the consistency of the peaks in both viewing time clusters was looked at more specifically with a peak existing in both clusters leading to a higher score than peaks that only existed in one. |
| *P_B_* | girls + 2  boys + 3  adults – 1 |  | if the same peak is present in the total 100%:  girls + 1  boys + 1.5  if the other sex’s peak is present in the total 100%:  girls + 0.5  boys + 0.5  adults – 0.5 |  | This score combines the idea of *P_1_* with the higher valuation of boy peaks as a possibly superior predictor for pedophilia. |
| *P_C_* | girls + 2  boys + 2  adults – 1 |  | if the same peak is present in the total 100%:  girls + 2  boys + 2  if the other sex’s peak is present in the total 100%:  girls + 1  boys + 1  adults – 1 |  | This score is a variation of *P_1_* in which the weighting of the two viewing time clusters *total 100%* and *lower 50%* was adjusted to have the same impact on the score. |
| *P_D_* | girls + 2  boys + 2  adults – 1 | girls + 1  boys + 1  adults – 0.5 | girls + 1  boys + 1  adults – 0.5 |  | This score includes the *lower 75%* but weight the *total 100%* higher than the other two viewing time clusters. |
| *P_E_* | + number of child peaks  – 0.5 * number of adult peaks | + number of child peaks  – 0.5 * number of adult peaks | + number of child peaks  – 0.5 * number of adult peaks |  | This score considers the number of peaks in each category instead of only whether any peak exists or not. |
| *P_F_* (cons.) | + number of child peaks  – 0.7 * number of adult peaks | + number of child peaks  – 0.6 * number of adult peaks | + 0.9* number of child peaks  – 0.6 * number of adult peaks | + number of child peaks  – 0.6 * number of adult peaks | For these two scores, the weighting of the peaks was based on their correlation with the validity criterium fantasy. |
| *P_F_* (prog.) | + number of child peaks  – 0.5 * number of adult peaks | + 0.9* number of child peaks  – 0.4 * number of adult peaks | + number of child peaks  – 0.5 * number of adult peaks | + number of child peaks  – 0.5 * number of adult peaks |  |

*Note.* These VT scoring algorithms were also explored but not included in the main text because they performed similarly or worse than the presented scores and did not yield additional insights in to the mechanisms of the VT methods.

**Table S2**

*Correlations of the absolute viewing time scores and number of peaks with the absolute rating and ESIQ scores.*

|  |  | | rating | | | | | | | | | |  | ESIQ | | | | |
| --- | --- | --- | --- | --- | --- | --- | --- | --- | --- | --- | --- | --- | --- | --- | --- | --- | --- | --- |
|  |  | | MT1 | MT2 | MT3 | MT4 | MT5 | FT1 | FT2 | FT3 | FT4 | FT5 |  | boys | men | girls | women | |
| absolute scores | *total 100%* | MT1 | .02^*^ | .08^**^ | .09^**^ | .11^**^ | .08^**^ | .05^**^ | .06^**^ | .06^**^ | .03^*^ | -.02^*^ |  | .04^**^ | .09^**^ | .04^**^ | | -.02^*^ |
|  |  | MT2 | .02 | .09^**^ | .12^**^ | .17^**^ | .13^**^ | .04^**^ | .05^**^ | .04^**^ | .00 | -.05^**^ |  | .05^**^ | .14^**^ | .02 | | -.06^**^ |
|  |  | MT3 | .01 | .09^**^ | .14^**^ | .23^**^ | .21^**^ | .02^*^ | .03^**^ | .03^**^ | -.04^**^ | -.12^**^ |  | .05^**^ | .24^**^ | .01 | | -.15^**^ |
|  |  | MT4 | .01 | .09^**^ | .14^**^ | .29^**^ | .31^**^ | .02 | .02^*^ | .03^*^ | .05^**^ | -.14^**^ |  | .05^**^ | .37^**^ | .01 | | -.21^**^ |
|  |  | MT5 | -.01 | .05^**^ | .09^**^ | .19^**^ | .36^**^ | .00 | .00 | -.01 | -.10^**^ | -.15^**^ |  | .01 | .38^**^ | -.01 | | -.25^**^ |
|  |  | FT1 | -.01 | .02^*^ | .02^*^ | .02 | -.02 | .16^**^ | .19^**^ | .25^**^ | .20^**^ | .06^**^ |  | .00 | -.02^*^ | .12^**^ | | .07^**^ |
|  |  | FT2 | -.03^**^ | .00 | .00 | .01 | -.04^**^ | .10^**^ | .17^**^ | .25^**^ | .27^**^ | .12^**^ |  | -.01 | -.05^**^ | .11^**^ | | .10^**^ |
|  |  | FT3 | -.05^**^ | -.03^**^ | -.04^**^ | -.05^**^ | -.09^**^ | .06^**^ | .12^**^ | .25^**^ | .32^**^ | .17^**^ |  | -.02^*^ | -.09^**^ | .09^**^ | | .15^**^ |
|  |  | FT4 | -.14^**^ | -.16^**^ | -.17^**^ | -.22^**^ | -.27^**^ | -.11^**^ | -.11^**^ | -.07^**^ | .22^**^ | .31^**^ |  | -.11^**^ | -.27^**^ | -.06^**^ | | .34^**^ |
|  |  | FT5 | -.14^**^ | -.16^**^ | -.18^**^ | -.23^**^ | -.27^**^ | -.12^**^ | -.13^**^ | -.12^**^ | -.02 | .18^**^ |  | -.10^**^ | -.27^**^ | -.07^**^ | | .32^**^ |
|  | *lower 75%* | MT1 | -.01 | .06^**^ | .06^**^ | .09^**^ | .08^**^ | .03^*^ | .04^**^ | .04^**^ | .02 | -.02 |  | .03^*^ | .09^**^ | .02 | | -.01 |
|  |  | MT2 | -.01 | .06^**^ | .09^**^ | .14^**^ | .11^**^ | .02 | .03^**^ | .03^**^ | .00 | -.04^**^ |  | .03^**^ | .14^**^ | .01 | | .05^**^ |
|  |  | MT3 | -.01 | -.07^**^ | .11^**^ | .20^**^ | .18^**^ | .01 | .02^*^ | .03^*^ | .02^*^ | -.09^*^ |  | .04^**^ | .21^**^ | .01 | | -.11^**^ |
|  |  | MT4 | -.01 | .08^**^ | .13^**^ | .27^**^ | .27^**^ | .01 | .02 | .03^*^ | -.03^**^ | -.12^**^ |  | .04^**^ | .33^**^ | .00 | | -.18^**^ |
|  |  | MT5 | -.03^*^ | .03^**^ | .07^**^ | .18^**^ | .35^**^ | -.01 | -.01 | -.02 | -.09^**^ | -.14^**^ |  | .00 | .36^**^ | -.02^*^ | | -.23^**^ |
|  |  | FT1 | -.03^**^ | .00 | .00 | -.01 | -.03^**^ | .12^**^ | .16^**^ | .21^**^ | .18^**^ | .06^**^ |  | -.02 | -.03^**^ | .11^**^ | | .09^**^ |
|  |  | FT2 | -.05^**^ | -.02^*^ | -.02^*^ | -.03^*^ | -.04^**^ | .08^**^ | .15^**^ | .22^**^ | .24^**^ | .11^**^ |  | -.02^*^ | -.05^**^ | .09^**^ | | .10^**^ |
|  |  | FT3 | -.06^**^ | -.04^**^ | -.04^**^ | -.05^**^ | -.08^**^ | .06^**^ | .12^**^ | .23^**^ | .30^**^ | .16^**^ |  | -.03^*^ | -.08^**^ | .08^**^ | | .15^**^ |
|  |  | FT4 | -.14^**^ | -.15^**^ | -.17^**^ | -.21^**^ | -.24^**^ | -.10^**^ | -.09^**^ | -.06^**^ | .24^**^ | .30^**^ |  | -.10^**^ | -.25^**^ | -.05^**^ | | .31^**^ |
|  |  | FT5 | -.15^**^ | -.16^**^ | -.18^**^ | -.23^**^ | -.26^**^ | -.13^**^ | -.13^**^ | -.12^**^ | -.01 | .18^**^ |  | -.10^**^ | -.26^**^ | -.07^**^ | | .31^**^ |

|  |  | | | rating | | | | | | | | | |  | ESIQ | | | | |
| --- | --- | --- | --- | --- | --- | --- | --- | --- | --- | --- | --- | --- | --- | --- | --- | --- | --- | --- | --- |
|  |  | | | MT1 | MT2 | MT3 | MT4 | MT5 | FT1 | FT2 | FT3 | FT4 | FT5 |  | boys | men | girls | women | |
| absolute scores | *lower 50%* | | MT1 | -.03^**^ | .03^**^ | .04^**^ | .07^**^ | .07^**^ | .00 | .01 | .02^*^ | .01 | -.02 |  | .01 | .09^**^ | .00 | | .00 |
|  |  |  | MT2 | -.03^**^ | .04^**^ | .06^**^ | .11^**^ | .09^**^ | .00 | .02 | .02 | .00 | -.03^**^ |  | .02 | .12^**^ | .00 | | -.03^*^ |
|  |  |  | MT3 | -.03^*^ | .05^**^ | .08^**^ | .15^**^ | .14^**^ | .00 | .01 | .02 | -.02 | -.07^**^ |  | .02^*^ | .17^**^ | .00 | | -.07^**^ |
|  |  |  | MT4 | -.02^*^ | .06^**^ | .11^**^ | .22^**^ | .21^**^ | .00 | .02 | .02^*^ | -.02 | -.09^**^ |  | .03^**^ | .27^**^ | .00 | | -.13^**^ |
|  |  |  | MT5 | -.04^**^ | .02 | .05^**^ | .16^**^ | .32^**^ | -.02^*^ | -.02 | -.02^*^ | -.08^**^ | -.12^**^ |  | -.01 | .33^**^ | -.03^*^ | | -.20^**^ |
|  |  |  | FT1 | -.04^**^ | -.01 | -.02 | -.02 | -.04^**^ | .09^**^ | .13^**^ | .17^**^ | .16^**^ | .06^**^ |  | -.02 | -.03^**^ | .09^**^ | | .09^**^ |
|  |  |  | FT2 | -.06^**^ | -.03^**^ | -.03^**^ | -.03^**^ | -.04^**^ | .06^**^ | .12^**^ | .19^**^ | .21^**^ | .10^**^ |  | -.03^**^ | -.04^**^ | .08^**^ | | .10^**^ |
|  |  |  | FT3 | -.07^**^ | -.04^**^ | -.05^**^ | -.06^**^ | -.08^**^ | .04^**^ | .10^**^ | .20^**^ | .27^**^ | .15^**^ |  | -.04^**^ | -.08^**^ | .07^**^ | | .14^**^ |
|  |  |  | FT4 | -.13^**^ | -.14^**^ | -.15^**^ | -.19^**^ | -.22^**^ | -.09^**^ | -.08^**^ | -.04^**^ | .26^**^ | .29^**^ |  | -.09^**^ | -.22^**^ | -.04^**^ | | .29^**^ |
|  |  |  | FT5 | -.14^**^ | -.16^**^ | -.18^**^ | -.22^**^ | -.25^**^ | -.12^**^ | -.13^**^ | -.12^**^ | .00 | .19^**^ |  | -.10^**^ | -.25^**^ | -.07^**^ | | .30^**^ |
|  | *middle 50%* | | MT1 | .01 | .07^**^ | .08^**^ | .10^**^ | .08^**^ | .04^**^ | .05^**^ | .05^**^ | .02^*^ | -.02 |  | .03^**^ | .09^**^ | .02^*^ | | -.02 |
|  |  |  | MT2 | .00 | .08^**^ | .11^**^ | .16^**^ | .13^**^ | .03^**^ | .04^**^ | .04^**^ | .00 | -.05^**^ |  | .04^**^ | .15^**^ | .01 | | -.06^**^ |
|  |  |  | MT3 | .01 | .08^**^ | .13^**^ | .22^**^ | .20^**^ | .02 | .03^**^ | .03^**^ | -.03^**^ | -.11^**^ |  | .05^**^ | .23^**^ | .01 | | -.13^**^ |
|  |  |  | MT4 | .00 | .09^**^ | .14^**^ | .30^**^ | .30^**^ | .01 | .02^*^ | .03^*^ | -.04^**^ | -.13^**^ |  | .04^**^ | .36^**^ | .01 | | -.21^**^ |
|  |  |  | MT5 | -.02 | .04^**^ | .08^**^ | .18^**^ | .36^**^ | -.01 | -.01 | -.01 | -.10^**^ | -.15^**^ |  | .01 | .38^**^ | -.02 | | .24^**^ |
|  |  |  | FT1 | -.03^*^ | .00 | .01 | .00 | -.03^*^ | .14^**^ | .18^**^ | .23^**^ | .19^**^ | .06^**^ |  | -.02 | -.03^*^ | .12^**^ | | .08^**^ |
|  |  |  | FT2 | -.04^**^ | -.02 | -.01 | -.02 | -.04^**^ | .09^**^ | .16^**^ | .24^**^ | .26^**^ | .12^**^ |  | -.02 | -.05^**^ | .10^**^ | | .10^**^ |
|  |  |  | FT3 | -.05^**^ | -.03^**^ | -.04^**^ | -.05^**^ | -.08^**^ | .06^**^ | .12^**^ | .24^**^ | .31^**^ | .17^**^ |  | -.02^*^ | -.08^**^ | .09^**^ | | .14^**^ |
|  |  |  | FT4 | -.14^**^ | -.15^**^ | -.17^**^ | -.21^**^ | -.25^**^ | -.10^**^ | -.10^**^ | -.07^**^ | .22^**^ | .30^**^ |  | -.10^**^ | -.25^**^ | -.05^**^ | | .31^**^ |
|  |  |  | FT5 | -.14^**^ | -.15^**^ | -.18^**^ | -.22^**^ | -.26^**^ | -.12^**^ | -.13^**^ | -.12^**^ | -.01 | .18^**^ |  | -.10^**^ | -.26^**^ | -.07^**^ | | .31^**^ |
| number of peaks | *total 100%* | cons. | MT1 | .10^**^ | .11^**^ | .10^**^ | .08^**^ | .05^**^ | .08^**^ | .07^**^ | .05^**^ | -.04^**^ | -.07^**^ |  | .11^**^ | .04^**^ | .08^**^ | | -.05^**^ |
|  |  |  | MT2 | .11^**^ | .13^**^ | .13^**^ | .14^**^ | .10^**^ | .04^**^ | .04^**^ | .02 | -.03^**^ | -.07^**^ |  | .10^**^ | .08^**^ | .02 | | -.08^**^ |
|  |  |  | MT3 | .06^**^ | .10^**^ | .15^**^ | .19^**^ | .15^**^ | .03^**^ | .03^**^ | .01 | -.08^**^ | -.15^**^ |  | .06^**^ | .17^**^ | .02^*^ | | -.20^**^ |
|  |  |  | MT4 | .06^**^ | .11^**^ | .15^**^ | .30^**^ | .27^**^ | .01 | .01 | .00 | -.09^**^ | -.18^**^ |  | .07^**^ | .35^**^ | .00 | | -.27^**^ |
|  |  |  | MT5 | .04^**^ | .06^**^ | .09^**^ | .16^**^ | .40^**^ | .01 | .00 | -.03^**^ | -.18^**^ | -.25^**^ |  | .03^*^ | .42^**^ | -.01 | | -.38^**^ |
|  |  |  | FT1 | .08^**^ | .08^**^ | .08^**^ | .06^**^ | .03^*^ | .21^**^ | .23^**^ | .24^**^ | .12^**^ | .00 |  | .02^*^ | -.01^**^ | .14^**^ | | -.02^*^ |
|  |  |  | FT2 | .05^**^ | .05^**^ | .05^**^ | .04^**^ | .02^*^ | .11^**^ | .17^**^ | .17^**^ | .13^**^ | .03^**^ |  | .04^**^ | .00 | .09^**^ | | -.02 |
|  |  |  | FT3 | .04^**^ | .04^**^ | .04^**^ | .02^*^ | .00 | .12^**^ | .16^**^ | .27^**^ | .21^**^ | .06^**^ |  | .02 | -.01 | .13^**^ | | .01 |
|  |  |  | FT4 | -.06^**^ | -.07^**^ | -.09^**^ | -.13^**^ | -.14^**^ | -.06^**^ | -.06^**^ | -.07^**^ | .23^**^ | .21^**^ |  | -.05^**^ | -.16^**^ | -.04^**^ | | .18^**^ |
|  |  |  | FT5 | -.07^**^ | -.09^**^ | -.11^**^ | -.15^**^ | -.17^**^ | -.07^**^ | -.09^**^ | -.11^**^ | -.21^**^ | -.02 |  | -.06^**^ | -.19^**^ | -.07^**^ | | .16^**^ |

|  | | |  | rating | | | | | | | | | |  | ESIQ | | | | |
| --- | --- | --- | --- | --- | --- | --- | --- | --- | --- | --- | --- | --- | --- | --- | --- | --- | --- | --- | --- |
|  |  |  |  | MT1 | MT2 | MT3 | MT4 | MT5 | FT1 | FT2 | FT3 | FT4 | FT5 |  | boys | men | girls | women | |
| number of peaks | *total 100%* | prog. | MT1 | .10^**^ | .10^**^ | .09^**^ | .08^**^ | .06^**^ | .09^**^ | .07^**^ | .06^**^ | -.03^*^ | -.07^**^ |  | .09^**^ | .05^**^ | .08^**^ | | -.06^**^ |
|  |  |  | MT2 | .11^**^ | .13^**^ | .13 | .14^**^ | .08^**^ | .04^**^ | .04^**^ | .02^*^ | -.03^**^ | -.07^**^ |  | .09^**^ | .08^**^ | .02 | | -.07^**^ |
|  |  |  | MT3 | .06^**^ | .09^**^ | .13^**^ | .16^**^ | .14^**^ | .03^*^ | .02^*^ | .01 | -.08^**^ | -.15^**^ |  | .05^**^ | .16^**^ | .01 | | -.19^**^ |
|  |  |  | MT4 | .08^**^ | .12^**^ | .16^**^ | .30^**^ | .26^**^ | .04^**^ | .03^**^ | .02 | -.07^**^ | -.15^**^ |  | .08^**^ | .31^**^ | .01 | | -.25^**^ |
|  |  |  | MT5 | .07^**^ | .09^**^ | .12^**^ | .16^**^ | .38^**^ | .03^**^ | .01 | -.02 | -.16^**^ | -.21^**^ |  | .04^**^ | .38^**^ | -.01 | | -.33^**^ |
|  |  |  | FT1 | .08^**^ | .08^**^ | .07^**^ | .05^**^ | .02 | .21^**^ | .23^**^ | .24^**^ | .12^**^ | -.02^*^ |  | .04^**^ | .00 | .14^**^ | | -.04^**^ |
|  |  |  | FT2 | .05^**^ | .05^**^ | .05^**^ | .03^**^ | .01 | .10^**^ | .15^**^ | .16^**^ | .13^**^ | .02 |  | .03^**^ | -.01 | .07^**^ | | -.02 |
|  |  |  | FT3 | .04^**^ | .04^**^ | .04^**^ | .02^*^ | .00 | .12^**^ | .15^**^ | .26^**^ | .18^**^ | .02^*^ |  | .02^*^ | .00 | .13^**^ | | -.01 |
|  |  |  | FT4 | -.04^**^ | -.06^**^ | -.07^**^ | -.10^**^ | -.12^**^ | -.05^**^ | -.05^**^ | -.06^**^ | .23^**^ | .20^**^ |  | -.04^**^ | -.14^**^ | -.04^**^ | | .16^**^ |
|  |  |  | FT5 | -.05^**^ | -.07^**^ | -.09^**^ | -.14^**^ | -.15^**^ | -.06^**^ | -.07^**^ | -.08^**^ | -.20^**^ | -.03^*^ |  | -.05^**^ | -.17^**^ | -.05^**^ | | .14^**^ |
|  | *lower 75%* | cons. | MT1 | .11^**^ | .12^**^ | .11^**^ | .08^**^ | .04^**^ | .09^**^ | .07^**^ | .05^**^ | -.03^*^ | -.07^**^ |  | .14^**^ | .05^**^ | .09^**^ | | -.07^**^ |
|  |  |  | MT2 | .09^**^ | .13^**^ | .13^**^ | .14^**^ | .11^**^ | .05^**^ | .04^**^ | .02^*^ | -.05^**^ | -.09^**^ |  | .11^**^ | .10^**^ | .02 | | -.11^**^ |
|  |  |  | MT3 | .09^**^ | .13^**^ | .17^**^ | .20^**^ | .17^**^ | .05^**^ | .05^**^ | .03^**^ | -.08^**^ | .14^**^ |  | .07^**^ | .18^**^ | .03^*^ | | -.20^**^ |
|  |  |  | MT4 | .07^**^ | .13^**^ | .19^**^ | .35^**^ | .29^**^ | .04^**^ | .03^**^ | .02 | -.09^**^ | -.19^**^ |  | .07^**^ | .40^**^ | .01 | | -.29^**^ |
|  |  |  | MT5 | .08^**^ | .09^**^ | .13^**^ | .21^**^ | .48^**^ | .03^**^ | .02 | -.02 | -.19^**^ | -.27^**^ |  | .05^**^ | .48^**^ | .00 | | -.44^**^ |
|  |  |  | FT1 | .09^**^ | .09^**^ | .08^**^ | .06^**^ | .03^*^ | .22^**^ | .23^**^ | .24^**^ | .11^**^ | -.03^**^ |  | .03^**^ | -.01 | .16^**^ | | -.05^**^ |
|  |  |  | FT2 | .05^**^ | .05^**^ | .05^**^ | .04^**^ | .01 | .10^**^ | .17^**^ | .19^**^ | .14^**^ | .03^**^ |  | .04^**^ | .00 | .10^**^ | | -.03^*^ |
|  |  |  | FT3 | .06^**^ | .06^**^ | .05^**^ | .03^**^ | .01 | .13^**^ | .18^**^ | .28^**^ | .20^**^ | .05^**^ |  | .04^**^ | -.01 | .13^**^ | | -.01 |
|  |  |  | FT4 | -.05^**^ | -.07^**^ | -.08^**^ | -.12^**^ | -.13^**^ | -.04^**^ | -.04^**^ | -.04^**^ | .26^**^ | .22^**^ |  | -.05^**^ | -.15^**^ | -.02 | | .18^**^ |
|  |  |  | FT5 | -.08^**^ | -.10^**^ | -.13^**^ | -.17^**^ | -.20^**^ | -.10^**^ | -.11^**^ | -.13^**^ | -.21^**^ | -.01 |  | -.07^**^ | -.21^**^ | -.08^**^ | | .18^**^ |
|  |  | prog. | MT1 | .16^**^ | .16^**^ | .15^**^ | .12^**^ | .08^**^ | .12^**^ | .10^**^ | .08^**^ | -.03^*^ | -.08^**^ |  | .17^**^ | .06^**^ | .11^**^ | | -.10^**^ |
|  |  |  | MT2 | .10^**^ | .14^**^ | .14^**^ | .15^**^ | .12^**^ | .07^**^ | .07^**^ | .04^**^ | -.05^**^ | -.11^**^ |  | .11^**^ | .10^**^ | .04^**^ | | -.12^**^ |
|  |  |  | MT3 | .08^**^ | .12^**^ | .15^**^ | .18^**^ | .16^**^ | .06^**^ | .05^**^ | .03^**^ | -.09^**^ | -.17^**^ |  | .07^**^ | .18^**^ | .03^*^ | | -.21^**^ |
|  |  |  | MT4 | .10^**^ | .15^**^ | .20^**^ | .35^**^ | .28^**^ | .06^**^ | .05^**^ | .03^**^ | -.09^**^ | -.18^**^ |  | .10^**^ | .37^**^ | .02 | | -.27^**^ |
|  |  |  | MT5 | .08^**^ | .11^**^ | .14^**^ | .20^**^ | .46^**^ | .05^**^ | .03^**^ | .00 | -.17^**^ | -.24^**^ |  | .06^**^ | .42^**^ | .01 | | -.38^**^ |
|  |  |  | FT1 | .12^**^ | .11^**^ | .10^**^ | .06^**^ | .03^*^ | .24^**^ | .25^**^ | .26^**^ | .10^**^ | -.05^**^ |  | .04^**^ | -.01 | .15^**^ | | -.05^**^ |
|  |  |  | FT2 | .04^**^ | .04^**^ | .04^**^ | .03^**^ | .01 | .09^**^ | .15^**^ | .17^**^ | .13^**^ | .02^*^ |  | .03^**^ | .00 | .08^**^ | | -.04^**^ |
|  |  |  | FT3 | .08^**^ | .09^**^ | .08^**^ | .06^**^ | .02^*^ | .14^**^ | .18^**^ | .28^**^ | .17^**^ | .02^*^ |  | .05^**^ | .00 | .13^**^ | | -.02^*^ |
|  |  |  | FT4 | -.04^**^ | -.07^**^ | -.08^**^ | -.11^**^ | -.11^**^ | -.03^**^ | -.04^**^ | -.03^**^ | .25^**^ | .21^**^ |  | -.05^**^ | -.14^**^ | -.02 | | .16^**^ |
|  |  |  | FT5 | -.07^**^ | -.09^**^ | -.11^**^ | -.15^**^ | -.18^**^ | -.08^**^ | -.09^**^ | -.11^**^ | -.21^**^ | -.02 |  | -.06^**^ | -.20^**^ | -.07^**^ | | .17^**^ |

|  |  | | | rating | | | | | | | | | | |  | ESIQ | | | | |
| --- | --- | --- | --- | --- | --- | --- | --- | --- | --- | --- | --- | --- | --- | --- | --- | --- | --- | --- | --- | --- |
|  |  | | | MT1 | MT2 | MT3 | | MT4 | MT5 | FT1 | FT2 | FT3 | FT4 | FT5 |  | boys | men | girls | women | |
| number of peaks | *lower 50%* | cons. | MT1 | .11^**^ | .14^**^ | .13^**^ | | .10^**^ | .07^**^ | .08^**^ | .07^**^ | .06^**^ | -.03^**^ | -.07^**^ |  | .13^**^ | .05^**^ | .07^**^ | | -.09^**^ |
|  |  |  | MT2 | .07^**^ | .10^**^ | .12^**^ | | .13^**^ | .12^**^ | .05^**^ | .05^**^ | .02 | -.05^**^ | -.10^**^ |  | .12^**^ | .11^**^ | .03^*^ | | -.12^**^ |
|  |  |  | MT3 | .10^**^ | .13^**^ | .17^**^ | | .22^**^ | .16^**^ | .06^**^ | .06^**^ | .05^**^ | -.07^**^ | -.14^**^ |  | .06^**^ | .19^**^ | .03^**^ | | -.19^**^ |
|  |  |  | MT4 | .07^**^ | .13^**^ | .19^**^ | | .32^**^ | .23^**^ | .04^**^ | .03^**^ | .02 | -.08^**^ | -.17^**^ |  | .09^**^ | .33^**^ | .02^*^ | | -.24^**^ |
|  |  |  | MT5 | .08^**^ | .09^**^ | .13^**^ | | .24^**^ | .51^**^ | .03^**^ | .01 | -.02 | -.20^**^ | -.29^**^ |  | .06^**^ | .53^**^ | .01 | | -.47^**^ |
|  |  |  | FT1 | .09^**^ | .09^**^ | .08^**^ | | .05^**^ | .01 | .20^**^ | .20^**^ | .21^**^ | .08^**^ | -.04^**^ |  | .04^**^ | -.01 | .13^**^ | | -.04^**^ |
|  |  |  | FT2 | .03^**^ | .04^**^ | .05^**^ | | .03^**^ | .01 | .08^**^ | .15^**^ | .17^**^ | .10^**^ | .00 |  | .04^**^ | -.01 | .10^**^ | | -.04^**^ |
|  |  |  | FT3 | .04^**^ | .05^**^ | .04^**^ | | .02 | .00 | .11^**^ | .16^**^ | .25^**^ | .19^**^ | .05^**^ |  | .01 | -.02 | .10^**^ | | .00 |
|  |  |  | FT4 | -.03^**^ | -.05^**^ | -.07^**^ | | -.11^**^ | -.11^**^ | -.04^**^ | -.04^**^ | -.03^**^ | .25^**^ | .21^**^ |  | -.05^**^ | -.15^**^ | -.03^**^ | | .16^**^ |
|  |  |  | FT5 | -.08^**^ | -.11^***^ | | -.13^**^ | -.18^**^ | -.21^**^ | -.10^**^ | -.11^**^ | -.13^**^ | -.20^**^ | .02 |  | -.06^**^ | -.22^**^ | -.08^**^ | | .20^**^ |
|  |  | prog. | MT1 | .13^**^ | .15^**^ | .15^**^ | | .13^**^ | .09^**^ | .10^**^ | .09^**^ | .06^**^ | -.05^**^ | -.10^**^ |  | .14^**^ | .09^**^ | .07^**^ | | -.13^**^ |
|  |  |  | MT2 | .07^**^ | .10^**^ | .12^**^ | | .14^**^ | .13^**^ | .05^**^ | .05^**^ | .02^*^ | -.07^**^ | -.12^**^ |  | .10^**^ | .13^**^ | .02^**^ | | -.15^**^ |
|  |  |  | MT3 | .12^**^ | .15^**^ | .18^**^ | | .21^**^ | .17^**^ | .08^**^ | .07^**^ | .05^**^ | -.08^**^ | -.15^**^ |  | .09^**^ | .18^**^ | .04^**^ | | -.19^**^ |
|  |  |  | MT4 | .10^**^ | .16^**^ | .21^**^ | | .32^**^ | .21^**^ | .08^**^ | .07^**^ | .05^**^ | -.07^**^ | -.16^**^ |  | .12^**^ | .30^**^ | .05^**^ | | -.21^**^ |
|  |  |  | MT5 | .09^**^ | .11^**^ | .14^**^ | | .24^**^ | .50^**^ | .04^**^ | .03^*^ | -.01 | -.19^**^ | -.27^**^ |  | .06^**^ | .48^**^ | .01 | | -.43^**^ |
|  |  |  | FT1 | .11^**^ | .10^**^ | .09^**^ | | .06^**^ | .03^*^ | .22^**^ | .24^**^ | .24^**^ | .10^**^ | -.05^**^ |  | .06^**^ | -.01 | .15^**^ | | -.05^**^ |
|  |  |  | FT2 | .03^**^ | .04^**^ | .04^**^ | | .03^*^ | .01 | .08^**^ | .14^**^ | .16^**^ | .10^**^ | .00 |  | .03^**^ | -.01 | .08^**^ | | -.05^**^ |
|  |  |  | FT3 | .07^**^ | .07^**^ | .06^**^ | | .05^**^ | .02 | .12^**^ | .16^**^ | .24^**^ | .15^**^ | .01 |  | .02^*^ | -.01 | .10^**^ | | -.02 |
|  |  |  | FT4 | -.03^*^ | -.05^**^ | -.06^**^ | | -.10^**^ | -.10^**^ | -.02^*^ | -.03^*^ | -.02 | .25^**^ | .19^**^ |  | -.05^**^ | -.13^**^ | -.02 | | .14^**^ |
|  |  |  | FT5 | -.07^**^ | -.10^**^ | -.12^**^ | | -.16^**^ | -.19^**^ | -.08^**^ | -.09^**^ | -.10^**^ | -.19^**^ | .01 |  | -.06^**^ | -.21^**^ | -.06^**^ | | .19^**^ |
|  | *middle 50%* | cons. | MT1 | .13^**^ | .13^**^ | .12^**^ | | .09^**^ | .06^**^ | .11^**^ | .09^**^ | .07^**^ | -.02 | -.06^**^ |  | .11^**^ | .04^**^ | .09^**^ | | -.08^**^ |
|  |  |  | MT2 | .09^**^ | .14^**^ | .14^**^ | | .14^**^ | .11^**^ | .04^**^ | .05^**^ | .03^**^ | -.04^**^ | -.08^**^ |  | .10^**^ | .09^**^ | .01 | | -.09^**^ |
|  |  |  | MT3 | .09^**^ | .13^**^ | .17^**^ | | .21^**^ | .17^**^ | .05^**^ | .05^**^ | .03^**^ | -.07^**^ | -.14^**^ |  | .09^**^ | .19^**^ | .04^**^ | | -.21^**^ |
|  |  |  | MT4 | .09^**^ | .14^**^ | .19^**^ | | .36^**^ | .31^**^ | .05^**^ | .04^**^ | .02^*^ | -.10^**^ | -.19^**^ |  | .07^**^ | .39^**^ | .01 | | -.30^**^ |
|  |  |  | MT5 | .05^**^ | .08^**^ | .11^**^ | | .17^**^ | .44^**^ | .02 | .01 | -.02 | -.18^**^ | -.26^**^ |  | .06^**^ | .45^**^ | .00 | | -.41^**^ |
|  |  |  | FT1 | .08^**^ | .08^**^ | .07^**^ | | .05^**^ | .02 | .22^**^ | .23^**^ | .23^**^ | .11^**^ | -.03^**^ |  | .02^*^ | -.01 | .15^**^ | | -.05^**^ |
|  |  |  | FT2 | .04^**^ | .04^**^ | .05^**^ | | .04^**^ | .01 | .10^**^ | .18^**^ | .19^**^ | .15^**^ | .04^**^ |  | .01 | .00 | .09^**^ | | -.02 |
|  |  |  | FT3 | .06^**^ | .07^**^ | .06^**^ | | .04^**^ | .00 | .12^**^ | .17^**^ | .27^**^ | .21^**^ | .06^**^ |  | .03^**^ | -.01 | .12^**^ | | .00 |
|  |  |  | FT4 | -.05^**^ | -.07^**^ | -.09^**^ | | -.13^**^ | -.13^**^ | -.05^**^ | -.05^**^ | -.06^**^ | .23^**^ | .21^**^ |  | -.05^**^ | -.16^**^ | -.03^*^ | | .18^**^ |
|  |  |  | FT5 | -.07^**^ | -.09^**^ | -.11^**^ | | -.16^**^ | -.18^**^ | -.08^**^ | -.10^**^ | -.12^**^ | -.20^**^ | -.02 |  | -.07^**^ | -.20^**^ | -.08^**^ | | .17^**^ |

|  |  | | | rating | | | | | | | | | |  | ESIQ | | | | |
| --- | --- | --- | --- | --- | --- | --- | --- | --- | --- | --- | --- | --- | --- | --- | --- | --- | --- | --- | --- |
|  |  | | | MT1 | MT2 | MT3 | MT4 | MT5 | FT1 | FT2 | FT3 | FT4 | FT5 |  | boys | men | girls | women | |
| number of peaks | *middle 50%* | prog. | MT1 | .12^**^ | .13^**^ | .13^**^ | .11^**^ | .08^**^ | .11^**^ | .09^**^ | .07^**^ | -.03^*^ | -.07^**^ |  | .13^**^ | .07^**^ | .09^**^ | | -.09^**^ |
|  |  |  | MT2 | .11^**^ | .16^**^ | .16^**^ | .18^**^ | .12^**^ | .07^**^ | .08^**^ | .05^**^ | -.04^**^ | -.10^**^ |  | .13^**^ | .11^**^ | .04^**^ | | -.11^**^ |
|  |  |  | MT3 | .09^**^ | .13^**^ | .17^**^ | .19^**^ | .17^**^ | .06^**^ | .05^**^ | .04^**^ | -.08^**^ | -.16^**^ |  | .07^**^ | .18^**^ | .03^**^ | | -.21^**^ |
|  |  |  | MT4 | .11^**^ | .16^**^ | .21^**^ | .35^**^ | .30^**^ | .07^**^ | .06^**^ | .04^**^ | -.08^**^ | -.17^**^ |  | .09^**^ | .36^**^ | .03^*^ | | -.27^**^ |
|  |  |  | MT5 | .05^**^ | .08^**^ | .11^**^ | .17^**^ | .42^**^ | .02^*^ | .01 | -.02 | -.17^**^ | -.23^**^ |  | .05^**^ | .41^**^ | -.01 | | -.37^**^ |
|  |  |  | FT1 | .11^**^ | .10^**^ | .10^**^ | .07^**^ | .03^**^ | .23^**^ | .24^**^ | .26^**^ | .11^**^ | -.04^**^ |  | .06^**^ | .00 | .17^**^ | | -.05^**^ |
|  |  |  | FT2 | .05^**^ | .05^**^ | .05^**^ | .04^**^ | .02 | .09^**^ | .16^**^ | .17^**^ | .15^**^ | .04^**^ |  | .02 | .00 | .08^**^ | | -.03^**^ |
|  |  |  | FT3 | .07^**^ | .08^**^ | .07^**^ | .05^**^ | .02 | .13^**^ | .17^**^ | .26^**^ | .18^**^ | .03^**^ |  | .05^**^ | .00 | .13^**^ | | -.02^*^ |
|  |  |  | FT4 | -.04^**^ | -.06^**^ | -.08^**^ | -.11^**^ | -.12^**^ | -.04^**^ | -.04^**^ | -.04^**^ | .23^**^ | .20^**^ |  | -.04^**^ | -.14^**^ | -.02 | | .16^**^ |
|  |  |  | FT5 | -.07^**^ | -.09^**^ | -.11^**^ | -.15^**^ | -.16^**^ | -.07^**^ | -.08^**^ | -.10^**^ | -.20^**^ | -.02 |  | -.07^**^ | -.18^**^ | -.07^**^ | | .16^**^ |

*Note.* * *p* < .05, ** *p* < .01. Abbreviations: *rating* = rating of pictures of children and adults in the viewing time task; *ESIQ* = self-report measure for past sexual behavior and fantasies regarding children and adults; *M* = male; *F* = female; *Tx* = Tanner class x (e.g., MT1 = male Tanner 1); *total 100%* = cluster using all viewing times; *lower 75%* = cluster excluding the highest viewing time per category; *lower 50%* = cluster excluding the two highest viewing times per category; *middle 50%* = cluster excluding the highest and lowest viewing time per category; *cons.* = conservative approach in which the peak had to differ from the mean of all other VTs by more than one SD; *prog.* = progressive approach in which the peak had to differ from the mean of all other VTs by more than half a SD.

**Table S3**

*Internal Consistencies (Cronbach’s* α*) of the absolute viewing time scores and number of peaks*

| Tanner Stage | Reliability ($\alpha$) | | |
| --- | --- | --- | --- |
|  | Absolute Scores | Peak Scores | |
|  |  | Cons. | Prog. |
| MT1 | .73 | .09 | .10 |
| MT2 | .73 | .04 | .09 |
| MT3 | .73 | .10 | .08 |
| MT4 | .76 | .23 | .21 |
| MT5 | .77 | .37 | .36 |
| FT1 | .77 | .20 | .16 |
| FT2 | .76 | .09 | .06 |
| FT3 | .78 | .16 | .17 |
| FT4 | .73 | .23 | .22 |
| FT5 | .80 | .30 | .28 |

*Note.* Abbreviations: *M* = male; *F* = female; *Tx* = Tanner class x (e.g., MT1 = male Tanner 1); *cons.* = conservative approach in which the peak had to differ from the mean of all other VTs by more than one SD; *prog.* = progressive approach in which the peak had to differ from the mean of all other VTs by more than half a SD.

**Table S4**

*Correlations of the viewing time scores and confounding variables.*

| Score | | general processing speed ($\overline{VT}$) | test sequence | age | occupation | education |
| --- | --- | --- | --- | --- | --- | --- |
| *D* | | -.26^**^ | .03^*^ | .01 | -.04^**^ | -.04^**^ |
| *D_x̅_* | | .02 | .02 | .00 | .04^**^ | -.05^**^ |
| *D_SD_* | | .04^**^ | .02 | .00 | .00 | -.03^**^ |
| *maxD* | | -.13^**^ | .03^**^ | .01 | -.04^**^ | -.01 |
| *D_lower 75%_* | | -.32^**^ | .02^*^ | .01 | -.05^**^ | -.05^**^ |
| *D_lower 50%_* | | -.33^**^ | .01 | .01 | -.05^**^ | -.06^**^ |
| *D_middle 50%_* | | -.30^**^ | .02^*^ | .01 | -.04^**^ | -.04^**^ |
| *cons.* | *P_1_* | .06^**^ | .02^*^ | .02 | .00 | -.01 |
|  | *P_2_* | .04^**^ | .02^*^ | .01 | -.01 | -.01 |
|  | *P_3_* | .07^**^ | .02 | .02 | .00 | -.01 |
|  | *P_4_* | .07^**^ | .02 | .02 | .00 | -.01 |
|  | *P_5_* | .07^**^ | .01 | .02 | .00 | -.01 |
|  | *RCP* | .09^**^ | .01 | .02^*^ | .00 | -.01 |
| *prog.* | *P_1_* | .08^**^ | .03^*^ | .01 | .02 | -.02 |
|  | *P_2_* | .06^**^ | .03^*^ | .01 | .01 | -.02 |
|  | *P_3_* | .08^**^ | .02 | .01 | .02 | -.02 |
|  | *P_4_* | .08^**^ | .02 | .01 | .01 | -.01 |
|  | *P_5_* | .07^**^ | .01 | .01 | .01 | -.02 |
|  | *RCP* | .09^**^ | .00 | .02 | .01 | -.01 |
| *Note.* * *p* < .05, ** *p* < .01. Abbreviations: VT = viewing time. VT Difference scores: *D* = simple difference index subtracting the mean VT for adult stimuli from the mean VT for child stimuli; *D_x̅_* = *D* divided by the mean of all VTs; *D_SD_* = *D* divided by the SD of all VTs; *maxD* = difference index using the maximum child and adult categories; *D_lower 75%_* = *D* calculated with the mean of the three lowest VTs per category; *D_lower 50%_* = *D* calculated with the mean of the two lowest VTs per category; *D_middle 50%_* = *D* calculated with the mean excluding the highest and lowest VT per category. VT peak scores: cons. = conservative approach in which the peak had to differ from the mean of all other VTs by more than one SD; prog. = progressive approach in which the peak had to differ from the mean of all other VTs by more than half a SD; *P_1_* = positive points were given for peaks in child categories in the *total 100%* or *lower 50%* cluster and negative points for peak in adult categories; *P_2_* = peaks in boy categories were weight higher than peaks in girl categories; *P_3_* = used the *total 100%*, *lower 75%* and *lower 50%* cluster; *P_4_* = used the *total 100%*, *lower 75%,* *lower 50%* and *middle 50%* cluster; *P_5_* = peaks in the *lower 75%* and *lower 50%* cluster were weight higher than peaks in the other clusters; RCP = absolute score only taking the child categories into account. | | | | | | |

### SPSS syntax of the scoring (self-report and VT)

```
*******************************************************
*******************************************************
****************scoring Self Report********************
*******************************************************
*******************************************************

*****rating of picture's sexual attractiveness*****
***absolute scoring (mean of the VT in each category)***

DATASET ACTIVATE DataSet1.
COMPUTE rating_abs_MT1=MEAN(rating11, rating26, rating30, rating34).
EXECUTE.

COMPUTE rating_abs_MT2=MEAN(rating16, rating32, rating38, rating3).
EXECUTE.

COMPUTE rating_abs_MT3=MEAN(rating13, rating23, rating35, rating6).
EXECUTE.

COMPUTE rating_abs_MT4=MEAN(rating10, rating20, rating25, rating36).
EXECUTE.

COMPUTE rating_abs_MT5=MEAN(rating12, rating27, rating29, rating8).
EXECUTE.

COMPUTE rating_abs_FT1=MEAN(rating15, rating2, rating31, rating4).
EXECUTE.

COMPUTE rating_abs_FT2=MEAN(rating18, rating1, rating24, rating9).
EXECUTE.

COMPUTE rating_abs_FT3=MEAN(rating0, rating14, rating17, rating5).
EXECUTE.

COMPUTE rating_abs_FT4=MEAN(rating21, rating22, rating39, rating7).
EXECUTE.

COMPUTE rating_abs_FT5=MEAN(rating19, rating28, rating33, rating37).
EXECUTE.


***relative scoring: mean rating of the adult-tanner-stages (T4, T5) – mean rating of the children-tanner-stages (T1-3)***

COMPUTE rating_rel_both=(MEAN(rating_abs_MT1, rating_abs_MT2, rating_abs_MT3, rating_abs_FT1,
    rating_abs_FT2, rating_abs_FT3)) - (MEAN(rating_abs_MT4, rating_abs_MT5, rating_abs_FT4,
    rating_abs_FT5)).
EXECUTE.


***maximum scoring: only rating of the maximum children- & adult-categories included***

COMPUTE rating_max_adult_both=MAX(rating_abs_FT4, rating_abs_FT5, rating_abs_MT4, rating_abs_MT5).
EXECUTE.

COMPUTE rating_max_child_both=MAX(rating_abs_FT1, rating_abs_FT2, rating_abs_FT3, rating_abs_MT1,
    rating_abs_MT2, rating_abs_MT3).
EXECUTE.

COMPUTE rating_maxsc_both=rating_max_child_both - rating_max_adult_both.
EXECUTE.


*****ESIQ*****
***absolute scoring (mean of the answers of each category***

*men*

COMPUTE esiq_abs_m=(ESIQ_9 + ESIQ_10 + ESIQ_16 + ESIQ_2 + ESIQ_6 + ESIQ_15) / 6.
EXECUTE.

*women*

COMPUTE esiq_abs_w=(ESIQ_4 + ESIQ_8 + ESIQ_22 + ESIQ_1 + ESIQ_11 + ESIQ_20) / 6.
EXECUTE.

*boys*

COMPUTE esiq_abs_b=(ESIQ_3 + ESIQ_14 + ESIQ_19 + ESIQ_13 + ESIQ_18 + ESIQ_23) / 6.
EXECUTE.

*girls*

COMPUTE esiq_abs_g=(ESIQ_5 + ESIQ_21 + ESIQ_24 + ESIQ_7 + ESIQ_12 + ESIQ_17) / 6.
EXECUTE.

*adult*

COMPUTE esiq_abs_adult=(ESIQ_9 + ESIQ_10 + ESIQ_16 + ESIQ_2 + ESIQ_6 + ESIQ_15 +
    ESIQ_4 + ESIQ_8 + ESIQ_22 + ESIQ_1 + ESIQ_11 + ESIQ_20) / 12.
EXECUTE.

*child*

COMPUTE esiq_abs_child=(ESIQ_3 + ESIQ_14 + ESIQ_19 + ESIQ_13 + ESIQ_18 + ESIQ_23 +
    ESIQ_5 + ESIQ_21 + ESIQ_24 + ESIQ_7 + ESIQ_12 + ESIQ_17) / 12.
EXECUTE.


***relative scoring***

COMPUTE esiq_rel_both=esiq_abs_child - esiq_abs_adult.
EXECUTE.


***maximum scoring***

COMPUTE esiq_max_both=MAX(esiq_abs_g, esiq_abs_b) - MAX(esiq_abs_w, esiq_abs_m).
EXECUTE.


***dichotomization of the relative scores***

DATASET ACTIVATE DataSet1.
IF  (rating_rel_both > 0) rating_rel_dichotom=1.
EXECUTE.

IF  (rating_maxsc_both > 0) rating_maxsc_dichotom=1.
EXECUTE.

IF  (esiq_rel_both > 0) esiq_rel_dichotom=1.
EXECUTE.

IF  (esiq_max_both > 0) esiq_max_dichotom=1.
EXECUTE.


*****admitted behavior and fantasy*****
*any child picture rated > 1*

DATASET ACTIVATE DataSet1.
IF  ((rating0 > 0) OR (rating1 > 0) OR (rating2 > 0) OR (rating3 > 0) OR (rating4 > 0) OR (rating5
    > 0) OR (rating6 > 0) OR (rating9 > 0) OR (rating11 > 0) OR (rating13 > 0) OR (rating14 > 0) OR
    (rating15 > 0) OR (rating16 > 0) OR (rating17 > 0) OR (rating18 > 0) OR (rating23 > 0) OR (rating24
    > 0) OR (rating26 > 0) OR (rating30 > 0) OR (rating31 > 0) OR (rating32 > 0) OR (rating34 > 0) OR
    (rating35 > 0) OR (rating38 > 0) ) positiverating=1.
EXECUTE.

*any behavior admitted*

IF  ((porno_2 = 1) OR (porno_3 = 1) OR (porno_4 = 1) OR (ESIQ_3 = 1) OR (ESIQ_5 = 1) OR (ESIQ_14 =
    1) OR (ESIQ_19 = 1) OR (ESIQ_21 = 1) OR (ESIQ_24 = 1)) beh_all=1.
EXECUTE.

*any contact behavior admitted*

IF  ((porno_3 = 1) OR (porno_4 = 1) OR (ESIQ_3 = 1) OR (ESIQ_5 = 1) OR (ESIQ_14 =
    1) OR (ESIQ_19 = 1) OR (ESIQ_21 = 1) OR (ESIQ_24 = 1)) beh_contact=1.
EXECUTE.

*CSAM use admitted*

IF (porno_2 = 1) beh_porno=1.
EXECUTE.

*any interest (fantasy &/ behavior) admitted*

IF  ((porno_2 = 1) OR (porno_3 = 1) OR (porno_4 = 1) OR (ESIQ_3 = 1) OR (ESIQ_5 = 1) OR (ESIQ_7 =
    1) OR (ESIQ_12 = 1) OR (ESIQ_13 = 1) OR (ESIQ_14 = 1) OR (ESIQ_17 = 1) OR (ESIQ_18 = 1) OR (ESIQ_19
    = 1) OR (ESIQ_21 = 1) OR (ESIQ_23 = 1) OR (ESIQ_24 = 1) ) anyinterest=1.
EXECUTE.

*any fantasy admitted*

IF  ((ESIQ_7 = 1) OR (ESIQ_12 = 1) OR (ESIQ_13 = 1) OR (ESIQ_17 = 1) OR (ESIQ_18 = 1) OR (ESIQ_23 = 1)) fantasy=1.
EXECUTE.

RECODE rating_rel_dichotom rating_maxsc_dichotom esiq_rel_dichotom esiq_max_dichotom positiverating beh_all beh_contact beh_porno
    anyinterest fantasy(SYSMIS=0).
EXECUTE.
```

```
*******************************************************
*******************************************************
****************scoring of the viewing times***********
*******************************************************
*******************************************************

***************using all pictures (total 100%)****************

*****absolute scoring (means of the VTs of the different categories)*****

DATASET ACTIVATE DataSet1.
COMPUTE VT_abs_MT1=MEAN(rating11_dur_clean, rating26_dur_clean, rating30_dur_clean,
    rating34_dur_clean).
EXECUTE.

COMPUTE VT_abs_MT2=MEAN(rating16_dur_clean, rating32_dur_clean, rating38_dur_clean,
    rating3_dur_clean).
EXECUTE.

COMPUTE VT_abs_MT3=MEAN(rating13_dur_clean, rating23_dur_clean, rating35_dur_clean,
    rating6_dur_clean).
EXECUTE.

COMPUTE VT_abs_MT4=MEAN(rating10_dur_clean, rating20_dur_clean, rating25_dur_clean,
    rating36_dur_clean).
EXECUTE.

COMPUTE VT_abs_MT5=MEAN(rating12_dur_clean, rating27_dur_clean, rating29_dur_clean,
    rating8_dur_clean).
EXECUTE.

COMPUTE VT_abs_FT1=MEAN(rating15_dur_clean, rating2_dur_clean, rating31_dur_clean,
    rating4_dur_clean).
EXECUTE.

COMPUTE VT_abs_FT2=MEAN(rating18_dur_clean, rating1_dur_clean, rating24_dur_clean,
    rating9_dur_clean).
EXECUTE.

COMPUTE VT_abs_FT3=MEAN(rating0_dur_clean, rating14_dur_clean, rating17_dur_clean,
    rating5_dur_clean).
EXECUTE.

COMPUTE VT_abs_FT4=MEAN(rating21_dur_clean, rating22_dur_clean, rating39_dur_clean,
    rating7_dur_clean).
EXECUTE.

COMPUTE VT_abs_FT5=MEAN(rating19_dur_clean, rating28_dur_clean, rating33_dur_clean,
    rating37_dur_clean).
EXECUTE.


*****relative scoring: mean viewing time of the adult-tanner-stages (T4, T5) – mean viewing time of the children-tanner-stages (T1-3)*****
***simple difference score D (no standardization)****

COMPUTE VT_rel_D=(MEAN(VT_abs_MT1, VT_abs_MT2, VT_abs_MT3, VT_abs_FT1, VT_abs_FT2, VT_abs_FT3)) -
    (MEAN(VT_abs_MT4, VT_abs_MT5, VT_abs_FT4, VT_abs_FT5)).
EXECUTE.


***standardized***

*D(m): difference score standardized with the mean of all viewing times*

COMPUTE VT_rel_D_mean=VT_rel_both / MEAN(rating0_dur_clean, rating1_dur_clean, rating2_dur_clean,
    rating3_dur_clean, rating4_dur_clean, rating5_dur_clean, rating6_dur_clean, rating7_dur_clean,
    rating8_dur_clean, rating9_dur_clean, rating10_dur_clean, rating11_dur_clean, rating12_dur_clean,
    rating13_dur_clean, rating14_dur_clean, rating15_dur_clean, rating16_dur_clean, rating17_dur_clean,
    rating18_dur_clean, rating19_dur_clean, rating20_dur_clean, rating21_dur_clean, rating22_dur_clean,
    rating23_dur_clean, rating24_dur_clean, rating25_dur_clean, rating26_dur_clean, rating27_dur_clean,
    rating28_dur_clean, rating29_dur_clean, rating30_dur_clean, rating31_dur_clean, rating32_dur_clean,
    rating33_dur_clean, rating34_dur_clean, rating35_dur_clean, rating36_dur_clean, rating37_dur_clean,
    rating38_dur_clean, rating39_dur_clean).
EXECUTE.

*D(SD): difference score standardized with the SD of all viewing times*

COMPUTE VT_rel_D_SD=VT_rel_D / SD(rating0_dur_clean, rating1_dur_clean, rating2_dur_clean,
    rating3_dur_clean, rating4_dur_clean, rating5_dur_clean, rating6_dur_clean, rating7_dur_clean,
    rating8_dur_clean, rating9_dur_clean, rating10_dur_clean, rating11_dur_clean, rating12_dur_clean,
    rating13_dur_clean, rating14_dur_clean, rating15_dur_clean, rating16_dur_clean, rating17_dur_clean,
    rating18_dur_clean, rating19_dur_clean, rating20_dur_clean, rating21_dur_clean, rating22_dur_clean,
    rating23_dur_clean, rating24_dur_clean, rating25_dur_clean, rating26_dur_clean, rating27_dur_clean,
    rating28_dur_clean, rating29_dur_clean, rating30_dur_clean, rating31_dur_clean, rating32_dur_clean,
    rating33_dur_clean, rating34_dur_clean, rating35_dur_clean, rating36_dur_clean, rating37_dur_clean,
    rating38_dur_clean, rating39_dur_clean).
EXECUTE.


*****maximum scoring: only viewing times of the maximum child- & adult-category included*****
***max values***

COMPUTE VT_max_adult_both=MAX(VT_abs_FT4, VT_abs_FT5, VT_abs_MT4, VT_abs_MT5).
EXECUTE.

COMPUTE VT_max_child_both=MAX(VT_abs_FT1, VT_abs_FT2, VT_abs_FT3, VT_abs_MT1, VT_abs_MT2, VT_abs_MT3).
EXECUTE.

***no standardization***

COMPUTE VT_maxsc_both=VT_max_child_both - VT_max_adult_both.
EXECUTE.


*****peak detection*****
***conservative (peak lies at least 1 SD above the mean of all VT)***
*by categories*

IF  ((VT_abs_MT1 > VT_abs_MT2) AND (VT_abs_MT1 > (MEAN(VT_abs_MT2, VT_abs_MT3, VT_abs_MT4,
    VT_abs_MT5, VT_abs_FT1, VT_abs_FT2, VT_abs_FT3, VT_abs_FT4, VT_abs_FT5) + SD(VT_abs_MT2,
    VT_abs_MT3, VT_abs_MT4, VT_abs_MT5, VT_abs_FT1, VT_abs_FT2, VT_abs_FT3, VT_abs_FT4, VT_abs_FT5))))
    VT_peak_kons_MT1=1.
EXECUTE.

IF  ((VT_abs_MT2 > VT_abs_MT1) AND (VT_abs_MT2 > VT_abs_MT3) AND (VT_abs_MT2 > (MEAN(VT_abs_MT1, VT_abs_MT3, VT_abs_MT4,
    VT_abs_MT5, VT_abs_FT1, VT_abs_FT2, VT_abs_FT3, VT_abs_FT4, VT_abs_FT5) + SD(VT_abs_MT1,
    VT_abs_MT3, VT_abs_MT4, VT_abs_MT5, VT_abs_FT1, VT_abs_FT2, VT_abs_FT3, VT_abs_FT4, VT_abs_FT5))))
    VT_peak_kons_MT2=1.
EXECUTE.

IF  ((VT_abs_MT3 > VT_abs_MT2) AND (VT_abs_MT3 > VT_abs_MT4) AND (VT_abs_MT3 > (MEAN(VT_abs_MT1, VT_abs_MT2, VT_abs_MT4,
    VT_abs_MT5, VT_abs_FT1, VT_abs_FT2, VT_abs_FT3, VT_abs_FT4, VT_abs_FT5) + SD(VT_abs_MT1,
    VT_abs_MT2, VT_abs_MT4, VT_abs_MT5, VT_abs_FT1, VT_abs_FT2, VT_abs_FT3, VT_abs_FT4, VT_abs_FT5))))
    VT_peak_kons_MT3=1.
EXECUTE.

IF  ((VT_abs_MT4 > VT_abs_MT3) AND (VT_abs_MT4 > VT_abs_MT5) AND (VT_abs_MT4 > (MEAN(VT_abs_MT1, VT_abs_MT2, VT_abs_MT3,
    VT_abs_MT5, VT_abs_FT1, VT_abs_FT2, VT_abs_FT3, VT_abs_FT4, VT_abs_FT5) + SD(VT_abs_MT1,
    VT_abs_MT2, VT_abs_MT3, VT_abs_MT5, VT_abs_FT1, VT_abs_FT2, VT_abs_FT3, VT_abs_FT4, VT_abs_FT5))))
    VT_peak_kons_MT4=1.
EXECUTE.

IF  ((VT_abs_MT5 > VT_abs_MT4) AND (VT_abs_MT5 > (MEAN(VT_abs_MT1, VT_abs_MT2, VT_abs_MT3,
    VT_abs_MT4, VT_abs_FT1, VT_abs_FT2, VT_abs_FT3, VT_abs_FT4, VT_abs_FT5) + SD(VT_abs_MT1,
    VT_abs_MT2, VT_abs_MT3, VT_abs_MT4, VT_abs_FT1, VT_abs_FT2, VT_abs_FT3, VT_abs_FT4, VT_abs_FT5))))
    VT_peak_kons_MT5=1.
EXECUTE.

IF  ((VT_abs_FT1 > VT_abs_FT2) AND (VT_abs_FT1 > (MEAN(VT_abs_MT1, VT_abs_MT2, VT_abs_MT3,
    VT_abs_MT4, VT_abs_MT5, VT_abs_FT2, VT_abs_FT3, VT_abs_FT4, VT_abs_FT5) + SD(VT_abs_MT1,
    VT_abs_MT2, VT_abs_MT3, VT_abs_MT4, VT_abs_MT5, VT_abs_FT2, VT_abs_FT3, VT_abs_FT4, VT_abs_FT5))))
    VT_peak_kons_FT1=1.
EXECUTE.

IF  ((VT_abs_FT2 > VT_abs_FT1) AND (VT_abs_FT2 > VT_abs_FT3) AND (VT_abs_FT2 > (MEAN(VT_abs_MT1, VT_abs_MT2, VT_abs_MT3,
    VT_abs_MT4, VT_abs_MT5, VT_abs_FT1, VT_abs_FT3, VT_abs_FT4, VT_abs_FT5) + SD(VT_abs_MT1,
    VT_abs_MT2, VT_abs_MT3, VT_abs_MT4, VT_abs_MT5, VT_abs_FT1, VT_abs_FT3, VT_abs_FT4, VT_abs_FT5))))
    VT_peak_kons_FT2=1.
EXECUTE.

IF  ((VT_abs_FT3 > VT_abs_FT2) AND (VT_abs_FT3 > VT_abs_FT4) AND (VT_abs_FT3 > (MEAN(VT_abs_MT1, VT_abs_MT2, VT_abs_MT3,
    VT_abs_MT4, VT_abs_MT5, VT_abs_FT1, VT_abs_FT2, VT_abs_FT4, VT_abs_FT5) + SD(VT_abs_MT1,
    VT_abs_MT2, VT_abs_MT3, VT_abs_MT4, VT_abs_MT5, VT_abs_FT1, VT_abs_FT2, VT_abs_FT4, VT_abs_FT5))))
    VT_peak_kons_FT3=1.
EXECUTE.

IF  ((VT_abs_FT4 > VT_abs_FT3) AND (VT_abs_FT4 > VT_abs_FT5) AND (VT_abs_FT4 > (MEAN(VT_abs_MT1, VT_abs_MT2, VT_abs_MT3,
    VT_abs_MT4, VT_abs_MT5, VT_abs_FT1, VT_abs_FT2, VT_abs_FT3, VT_abs_FT5) + SD(VT_abs_MT1,
    VT_abs_MT2, VT_abs_MT3, VT_abs_MT4, VT_abs_MT5, VT_abs_FT1, VT_abs_FT2, VT_abs_FT3, VT_abs_FT5))))
    VT_peak_kons_FT4=1.
EXECUTE.

IF  ((VT_abs_FT5 > VT_abs_FT4) AND (VT_abs_FT5 > (MEAN(VT_abs_MT1, VT_abs_MT2, VT_abs_MT3,
    VT_abs_MT4, VT_abs_MT5, VT_abs_FT1, VT_abs_FT2, VT_abs_FT3, VT_abs_FT4) + SD(VT_abs_MT1,
    VT_abs_MT2, VT_abs_MT3, VT_abs_MT4, VT_abs_MT5, VT_abs_FT1, VT_abs_FT2, VT_abs_FT3, VT_abs_FT4))))
    VT_peak_kons_FT5=1.
EXECUTE.

RECODE VT_peak_kons_MT1 VT_peak_kons_MT2 VT_peak_kons_MT3 VT_peak_kons_MT4 VT_peak_kons_MT5
    VT_peak_kons_FT1 VT_peak_kons_FT2 VT_peak_kons_FT3 VT_peak_kons_FT4 VT_peak_kons_FT5(SYSMIS=0).
EXECUTE.

COUNT VT_peak_kons_child=VT_peak_kons_MT1 VT_peak_kons_MT2 VT_peak_kons_MT3
    VT_peak_kons_FT1 VT_peak_kons_FT2 VT_peak_kons_FT3(1).
VARIABLE LABELS  VT_peak_kons_child 'children-peaks all4 kons'.
EXECUTE.

COUNT VT_peak_kons_girl=VT_peak_kons_FT1 VT_peak_kons_FT2 VT_peak_kons_FT3(1).
VARIABLE LABELS  VT_peak_kons_girl 'Mädchen-peaks all4 kons'.
EXECUTE.

COUNT VT_peak_kons_boy=VT_peak_kons_MT1 VT_peak_kons_MT2 VT_peak_kons_MT3(1).
VARIABLE LABELS  VT_peak_kons_boy 'Jungen-peaks all4 kons'.
EXECUTE.

COUNT VT_peak_kons_adult=VT_peak_kons_MT4 VT_peak_kons_MT5 VT_peak_kons_FT4 VT_peak_kons_FT5(1).
VARIABLE LABELS  VT_peak_kons_adult 'adult-peaks all4 kons'.
EXECUTE.


***progressive (1/2 SD)***
*by categories*

IF  ((VT_abs_MT1 > VT_abs_MT2) AND (VT_abs_MT1 > (MEAN(VT_abs_MT2, VT_abs_MT3, VT_abs_MT4,
    VT_abs_MT5, VT_abs_FT1, VT_abs_FT2, VT_abs_FT3, VT_abs_FT4, VT_abs_FT5) + (0.5 * SD(VT_abs_MT2,
    VT_abs_MT3, VT_abs_MT4, VT_abs_MT5, VT_abs_FT1, VT_abs_FT2, VT_abs_FT3, VT_abs_FT4, VT_abs_FT5)))))
    VT_peak_prog_MT1=1.
EXECUTE.

IF  ((VT_abs_MT2 > VT_abs_MT1) AND (VT_abs_MT2 > VT_abs_MT3) AND (VT_abs_MT2 > (MEAN(VT_abs_MT1, VT_abs_MT3, VT_abs_MT4,
    VT_abs_MT5, VT_abs_FT1, VT_abs_FT2, VT_abs_FT3, VT_abs_FT4, VT_abs_FT5) + (0.5 * SD(VT_abs_MT1,
    VT_abs_MT3, VT_abs_MT4, VT_abs_MT5, VT_abs_FT1, VT_abs_FT2, VT_abs_FT3, VT_abs_FT4, VT_abs_FT5)))))
    VT_peak_prog_MT2=1.
EXECUTE.

IF  ((VT_abs_MT3 > VT_abs_MT2) AND (VT_abs_MT3 > VT_abs_MT4) AND (VT_abs_MT3 > (MEAN(VT_abs_MT1, VT_abs_MT2, VT_abs_MT4,
    VT_abs_MT5, VT_abs_FT1, VT_abs_FT2, VT_abs_FT3, VT_abs_FT4, VT_abs_FT5) + (0.5 * SD(VT_abs_MT1,
    VT_abs_MT2, VT_abs_MT4, VT_abs_MT5, VT_abs_FT1, VT_abs_FT2, VT_abs_FT3, VT_abs_FT4, VT_abs_FT5)))))
    VT_peak_prog_MT3=1.
EXECUTE.

IF  ((VT_abs_MT4 > VT_abs_MT3) AND (VT_abs_MT4 > VT_abs_MT5) AND (VT_abs_MT4 > (MEAN(VT_abs_MT1, VT_abs_MT2, VT_abs_MT3,
    VT_abs_MT5, VT_abs_FT1, VT_abs_FT2, VT_abs_FT3, VT_abs_FT4, VT_abs_FT5) + (0.5 * SD(VT_abs_MT1,
    VT_abs_MT2, VT_abs_MT3, VT_abs_MT5, VT_abs_FT1, VT_abs_FT2, VT_abs_FT3, VT_abs_FT4, VT_abs_FT5)))))
    VT_peak_prog_MT4=1.
EXECUTE.

IF  ((VT_abs_MT5 > VT_abs_MT4) AND (VT_abs_MT5 > (MEAN(VT_abs_MT1, VT_abs_MT2, VT_abs_MT3,
    VT_abs_MT4, VT_abs_FT1, VT_abs_FT2, VT_abs_FT3, VT_abs_FT4, VT_abs_FT5) + (0.5 * SD(VT_abs_MT1,
    VT_abs_MT2, VT_abs_MT3, VT_abs_MT4, VT_abs_FT1, VT_abs_FT2, VT_abs_FT3, VT_abs_FT4, VT_abs_FT5)))))
    VT_peak_prog_MT5=1.
EXECUTE.

IF  ((VT_abs_FT1 > VT_abs_FT2) AND (VT_abs_FT1 > (MEAN(VT_abs_MT1, VT_abs_MT2, VT_abs_MT3,
    VT_abs_MT4, VT_abs_MT5, VT_abs_FT2, VT_abs_FT3, VT_abs_FT4, VT_abs_FT5) + (0.5 * SD(VT_abs_MT1,
    VT_abs_MT2, VT_abs_MT3, VT_abs_MT4, VT_abs_MT5, VT_abs_FT2, VT_abs_FT3, VT_abs_FT4, VT_abs_FT5)))))
    VT_peak_prog_FT1=1.
EXECUTE.

IF  ((VT_abs_FT2 > VT_abs_FT1) AND (VT_abs_FT2 > VT_abs_FT3) AND (VT_abs_FT2 > (MEAN(VT_abs_MT1, VT_abs_MT2, VT_abs_MT3,
    VT_abs_MT4, VT_abs_MT5, VT_abs_FT1, VT_abs_FT3, VT_abs_FT4, VT_abs_FT5) + (0.5 * SD(VT_abs_MT1,
    VT_abs_MT2, VT_abs_MT3, VT_abs_MT4, VT_abs_MT5, VT_abs_FT1, VT_abs_FT3, VT_abs_FT4, VT_abs_FT5)))))
    VT_peak_prog_FT2=1.
EXECUTE.

IF  ((VT_abs_FT3 > VT_abs_FT2) AND (VT_abs_FT3 > VT_abs_FT4) AND (VT_abs_FT3 > (MEAN(VT_abs_MT1, VT_abs_MT2, VT_abs_MT3,
    VT_abs_MT4, VT_abs_MT5, VT_abs_FT1, VT_abs_FT2, VT_abs_FT4, VT_abs_FT5) + (0.5 * SD(VT_abs_MT1,
    VT_abs_MT2, VT_abs_MT3, VT_abs_MT4, VT_abs_MT5, VT_abs_FT1, VT_abs_FT2, VT_abs_FT4, VT_abs_FT5)))))
    VT_peak_prog_FT3=1.
EXECUTE.

IF  ((VT_abs_FT4 > VT_abs_FT3) AND (VT_abs_FT4 > VT_abs_FT5) AND (VT_abs_FT4 > (MEAN(VT_abs_MT1, VT_abs_MT2, VT_abs_MT3,
    VT_abs_MT4, VT_abs_MT5, VT_abs_FT1, VT_abs_FT2, VT_abs_FT3, VT_abs_FT5) + (0.5 * SD(VT_abs_MT1,
    VT_abs_MT2, VT_abs_MT3, VT_abs_MT4, VT_abs_MT5, VT_abs_FT1, VT_abs_FT2, VT_abs_FT3, VT_abs_FT5)))))
    VT_peak_prog_FT4=1.
EXECUTE.

IF  ((VT_abs_FT5 > VT_abs_FT4) AND (VT_abs_FT5 > (MEAN(VT_abs_MT1, VT_abs_MT2, VT_abs_MT3,
    VT_abs_MT4, VT_abs_MT5, VT_abs_FT1, VT_abs_FT2, VT_abs_FT3, VT_abs_FT4) + (0.5 * SD(VT_abs_MT1,
    VT_abs_MT2, VT_abs_MT3, VT_abs_MT4, VT_abs_MT5, VT_abs_FT1, VT_abs_FT2, VT_abs_FT3, VT_abs_FT4)))))
    VT_peak_prog_FT5=1.
EXECUTE.


RECODE VT_peak_prog_MT1 VT_peak_prog_MT2 VT_peak_prog_MT3 VT_peak_prog_MT4 VT_peak_prog_MT5
    VT_peak_prog_FT1 VT_peak_prog_FT2 VT_peak_prog_FT3 VT_peak_prog_FT4 VT_peak_prog_FT5(SYSMIS=0).
EXECUTE.


COUNT VT_peak_prog_child=VT_peak_prog_MT1 VT_peak_prog_MT2 VT_peak_prog_MT3
    VT_peak_prog_FT1 VT_peak_prog_FT2 VT_peak_prog_FT3(1).
VARIABLE LABELS  VT_peak_prog_child 'children-peaks all4 prog'.
EXECUTE.

COUNT VT_peak_prog_girl=VT_peak_prog_FT1 VT_peak_prog_FT2 VT_peak_prog_FT3(1).
VARIABLE LABELS  VT_peak_prog_girl 'Mädchen-peaks all4 prog'.
EXECUTE.

COUNT VT_peak_prog_boy=VT_peak_prog_MT1 VT_peak_prog_MT2 VT_peak_prog_MT3(1).
VARIABLE LABELS  VT_peak_prog_boy 'Jungen-peaks all4 prog'.
EXECUTE.

COUNT VT_peak_prog_adult=VT_peak_prog_MT4 VT_peak_prog_MT5 VT_peak_prog_FT4 VT_peak_prog_FT5(1).
VARIABLE LABELS  VT_peak_prog_adult 'adult-peaks all4 prog'.
EXECUTE.


RECODE VT_peak_prog_child VT_peak_prog_adult VT_peak_prog_girl VT_peak_prog_boy(SYSMIS=0).
EXECUTE.


***************scoring of the three pictures with the lower VT (lower 75%)***************
*****absolute scoring*****
*MT1*

IF  (rating11_dur_clean = MAX(rating11_dur_clean, rating26_dur_clean, rating30_dur_clean,
    rating34_dur_clean)) VT_abs_low3_MT1=MEAN(rating26_dur_clean, rating30_dur_clean,
    rating34_dur_clean).
EXECUTE.

IF  (rating26_dur_clean = MAX(rating11_dur_clean, rating26_dur_clean, rating30_dur_clean,
    rating34_dur_clean)) VT_abs_low3_MT1=MEAN(rating11_dur_clean, rating30_dur_clean,
    rating34_dur_clean).
EXECUTE.

IF  (rating30_dur_clean = MAX(rating11_dur_clean, rating26_dur_clean, rating30_dur_clean,
    rating34_dur_clean)) VT_abs_low3_MT1=MEAN(rating11_dur_clean, rating26_dur_clean,
    rating34_dur_clean).
EXECUTE.

IF  (rating34_dur_clean = MAX(rating11_dur_clean, rating26_dur_clean, rating30_dur_clean,
    rating34_dur_clean)) VT_abs_low3_MT1=MEAN(rating11_dur_clean, rating26_dur_clean,
    rating30_dur_clean).
EXECUTE.

*MT2*

IF  (rating3_dur_clean = MAX(rating3_dur_clean, rating16_dur_clean, rating32_dur_clean,
    rating38_dur_clean)) VT_abs_low3_MT2=MEAN(rating16_dur_clean, rating32_dur_clean,
    rating38_dur_clean).
EXECUTE.

IF  (rating16_dur_clean = MAX(rating3_dur_clean, rating16_dur_clean, rating32_dur_clean,
    rating38_dur_clean)) VT_abs_low3_MT2=MEAN(rating3_dur_clean, rating32_dur_clean,
    rating38_dur_clean).
EXECUTE.

IF  (rating32_dur_clean = MAX(rating3_dur_clean, rating16_dur_clean, rating32_dur_clean,
    rating38_dur_clean)) VT_abs_low3_MT2=MEAN(rating3_dur_clean, rating16_dur_clean,
    rating38_dur_clean).
EXECUTE.

IF  (rating38_dur_clean = MAX(rating3_dur_clean, rating16_dur_clean, rating32_dur_clean,
    rating38_dur_clean)) VT_abs_low3_MT2=MEAN(rating3_dur_clean, rating16_dur_clean,
    rating32_dur_clean).
EXECUTE.

*MT3*

IF  (rating6_dur_clean = MAX(rating6_dur_clean, rating13_dur_clean, rating23_dur_clean,
    rating35_dur_clean)) VT_abs_low3_MT3=MEAN(rating13_dur_clean, rating23_dur_clean,
    rating35_dur_clean).
EXECUTE.

IF  (rating13_dur_clean = MAX(rating6_dur_clean, rating13_dur_clean, rating23_dur_clean,
    rating35_dur_clean)) VT_abs_low3_MT3=MEAN(rating6_dur_clean, rating23_dur_clean,
    rating35_dur_clean).
EXECUTE.

IF  (rating23_dur_clean = MAX(rating6_dur_clean, rating13_dur_clean, rating23_dur_clean,
    rating35_dur_clean)) VT_abs_low3_MT3=MEAN(rating6_dur_clean, rating13_dur_clean,
    rating35_dur_clean).
EXECUTE.

IF  (rating35_dur_clean = MAX(rating6_dur_clean, rating13_dur_clean, rating23_dur_clean,
    rating35_dur_clean)) VT_abs_low3_MT3=MEAN(rating6_dur_clean, rating13_dur_clean,
    rating23_dur_clean).
EXECUTE.

*MT4*

IF  (rating10_dur_clean = MAX(rating10_dur_clean, rating20_dur_clean, rating25_dur_clean,
    rating36_dur_clean)) VT_abs_low3_MT4=MEAN(rating20_dur_clean, rating25_dur_clean,
    rating36_dur_clean).
EXECUTE.

IF  (rating20_dur_clean = MAX(rating10_dur_clean, rating20_dur_clean, rating25_dur_clean,
    rating36_dur_clean)) VT_abs_low3_MT4=MEAN(rating10_dur_clean, rating25_dur_clean,
    rating36_dur_clean).
EXECUTE.

IF  (rating25_dur_clean = MAX(rating10_dur_clean, rating20_dur_clean, rating25_dur_clean,
    rating36_dur_clean)) VT_abs_low3_MT4=MEAN(rating10_dur_clean, rating20_dur_clean,
    rating36_dur_clean).
EXECUTE.

IF  (rating36_dur_clean = MAX(rating10_dur_clean, rating20_dur_clean, rating25_dur_clean,
    rating36_dur_clean)) VT_abs_low3_MT4=MEAN(rating10_dur_clean, rating20_dur_clean,
    rating25_dur_clean).
EXECUTE.

*MT5*

IF  (rating8_dur_clean = MAX(rating8_dur_clean, rating12_dur_clean, rating27_dur_clean,
    rating29_dur_clean)) VT_abs_low3_MT5=MEAN(rating12_dur_clean, rating27_dur_clean,
    rating29_dur_clean).
EXECUTE.

IF  (rating12_dur_clean = MAX(rating8_dur_clean, rating12_dur_clean, rating27_dur_clean,
    rating29_dur_clean)) VT_abs_low3_MT5=MEAN(rating8_dur_clean, rating27_dur_clean,
    rating29_dur_clean).
EXECUTE.

IF  (rating27_dur_clean = MAX(rating8_dur_clean, rating12_dur_clean, rating27_dur_clean,
    rating29_dur_clean)) VT_abs_low3_MT5=MEAN(rating8_dur_clean, rating12_dur_clean,
    rating29_dur_clean).
EXECUTE.

IF  (rating29_dur_clean = MAX(rating8_dur_clean, rating12_dur_clean, rating27_dur_clean,
    rating29_dur_clean)) VT_abs_low3_MT5=MEAN(rating8_dur_clean, rating12_dur_clean,
    rating27_dur_clean).
EXECUTE.

*FT1*

IF  (rating2_dur_clean = MAX(rating2_dur_clean, rating4_dur_clean, rating15_dur_clean,
    rating31_dur_clean)) VT_abs_low3_FT1=MEAN(rating4_dur_clean, rating15_dur_clean,
    rating31_dur_clean).
EXECUTE.

IF  (rating4_dur_clean = MAX(rating2_dur_clean, rating4_dur_clean, rating15_dur_clean,
    rating31_dur_clean)) VT_abs_low3_FT1=MEAN(rating2_dur_clean, rating15_dur_clean,
    rating31_dur_clean).
EXECUTE.

IF  (rating15_dur_clean = MAX(rating2_dur_clean, rating4_dur_clean, rating15_dur_clean,
    rating31_dur_clean)) VT_abs_low3_FT1=MEAN(rating2_dur_clean, rating4_dur_clean,
    rating31_dur_clean).
EXECUTE.

IF  (rating31_dur_clean = MAX(rating2_dur_clean, rating4_dur_clean, rating15_dur_clean,
    rating31_dur_clean)) VT_abs_low3_FT1=MEAN(rating2_dur_clean, rating4_dur_clean,
    rating15_dur_clean).
EXECUTE.

*FT2*

IF  (rating1_dur_clean = MAX(rating1_dur_clean, rating9_dur_clean, rating18_dur_clean,
    rating24_dur_clean)) VT_abs_low3_FT2=MEAN(rating9_dur_clean, rating18_dur_clean,
    rating24_dur_clean).
EXECUTE.

IF  (rating9_dur_clean = MAX(rating1_dur_clean, rating9_dur_clean, rating18_dur_clean,
    rating24_dur_clean)) VT_abs_low3_FT2=MEAN(rating1_dur_clean, rating18_dur_clean,
    rating24_dur_clean).
EXECUTE.

IF  (rating18_dur_clean = MAX(rating1_dur_clean, rating9_dur_clean, rating18_dur_clean,
    rating24_dur_clean)) VT_abs_low3_FT2=MEAN(rating1_dur_clean, rating9_dur_clean,
    rating24_dur_clean).
EXECUTE.

IF  (rating24_dur_clean = MAX(rating1_dur_clean, rating9_dur_clean, rating18_dur_clean,
    rating24_dur_clean)) VT_abs_low3_FT2=MEAN(rating1_dur_clean, rating9_dur_clean,
    rating18_dur_clean).
EXECUTE.

*FT3*

IF  (rating0_dur_clean = MAX(rating0_dur_clean, rating5_dur_clean, rating14_dur_clean,
    rating17_dur_clean)) VT_abs_low3_FT3=MEAN(rating5_dur_clean, rating14_dur_clean,
    rating17_dur_clean).
EXECUTE.

IF  (rating5_dur_clean = MAX(rating0_dur_clean, rating5_dur_clean, rating14_dur_clean,
    rating17_dur_clean)) VT_abs_low3_FT3=MEAN(rating0_dur_clean, rating14_dur_clean,
    rating17_dur_clean).
EXECUTE.

IF  (rating14_dur_clean = MAX(rating0_dur_clean, rating5_dur_clean, rating14_dur_clean,
    rating17_dur_clean)) VT_abs_low3_FT3=MEAN(rating0_dur_clean, rating5_dur_clean,
    rating17_dur_clean).
EXECUTE.

IF  (rating17_dur_clean = MAX(rating0_dur_clean, rating5_dur_clean, rating14_dur_clean,
    rating17_dur_clean)) VT_abs_low3_FT3=MEAN(rating0_dur_clean, rating5_dur_clean,
    rating14_dur_clean).
EXECUTE.

*FT4*

IF  (rating7_dur_clean = MAX(rating7_dur_clean, rating21_dur_clean, rating22_dur_clean,
    rating39_dur_clean)) VT_abs_low3_FT4=MEAN(rating21_dur_clean, rating22_dur_clean,
    rating39_dur_clean).
EXECUTE.

IF  (rating21_dur_clean = MAX(rating7_dur_clean, rating21_dur_clean, rating22_dur_clean,
    rating39_dur_clean)) VT_abs_low3_FT4=MEAN(rating7_dur_clean, rating22_dur_clean,
    rating39_dur_clean).
EXECUTE.

IF  (rating22_dur_clean = MAX(rating7_dur_clean, rating21_dur_clean, rating22_dur_clean,
    rating39_dur_clean)) VT_abs_low3_FT4=MEAN(rating7_dur_clean, rating21_dur_clean,
    rating39_dur_clean).
EXECUTE.

IF  (rating39_dur_clean = MAX(rating7_dur_clean, rating21_dur_clean, rating22_dur_clean,
    rating39_dur_clean)) VT_abs_low3_FT4=MEAN(rating7_dur_clean, rating21_dur_clean,
    rating22_dur_clean).
EXECUTE.

*FT5*

IF  (rating19_dur_clean = MAX(rating19_dur_clean, rating28_dur_clean, rating33_dur_clean,
    rating37_dur_clean)) VT_abs_low3_FT5=MEAN(rating28_dur_clean, rating33_dur_clean,
    rating37_dur_clean).
EXECUTE.

IF  (rating28_dur_clean = MAX(rating19_dur_clean, rating28_dur_clean, rating33_dur_clean,
    rating37_dur_clean)) VT_abs_low3_FT5=MEAN(rating19_dur_clean, rating33_dur_clean,
    rating37_dur_clean).
EXECUTE.

IF  (rating33_dur_clean = MAX(rating19_dur_clean, rating28_dur_clean, rating33_dur_clean,
    rating37_dur_clean)) VT_abs_low3_FT5=MEAN(rating19_dur_clean, rating28_dur_clean,
    rating37_dur_clean).
EXECUTE.

IF  (rating37_dur_clean = MAX(rating19_dur_clean, rating28_dur_clean, rating33_dur_clean,
    rating37_dur_clean)) VT_abs_low3_FT5=MEAN(rating19_dur_clean, rating28_dur_clean,
    rating33_dur_clean).
EXECUTE.


*****relative scoring*****

COMPUTE VT_rel_D_low3=(MEAN(VT_abs_low3_MT1, VT_abs_low3_MT2, VT_abs_low3_MT3, VT_abs_low3_FT1, VT_abs_low3_FT2,
    VT_abs_low3_FT3)) - (MEAN(VT_abs_low3_MT4, VT_abs_low3_MT5, VT_abs_low3_FT4, VT_abs_low3_FT5)).
EXECUTE.


*****peak detection*****
***conservative (1 SD)***
*by categories*

IF  ((VT_abs_low3_MT1 > VT_abs_low3_MT2) AND (VT_abs_low3_MT1 > (MEAN(VT_abs_low3_MT2, VT_abs_low3_MT3, VT_abs_low3_MT4,
    VT_abs_low3_MT5, VT_abs_low3_FT1, VT_abs_low3_FT2, VT_abs_low3_FT3, VT_abs_low3_FT4, VT_abs_low3_FT5) + SD(VT_abs_low3_MT2,
    VT_abs_low3_MT3, VT_abs_low3_MT4, VT_abs_low3_MT5, VT_abs_low3_FT1, VT_abs_low3_FT2, VT_abs_low3_FT3, VT_abs_low3_FT4, VT_abs_low3_FT5))))
    VT_peak_low3_kons_MT1=1.
EXECUTE.

IF  ((VT_abs_low3_MT2 > VT_abs_low3_MT1) AND (VT_abs_low3_MT2 > VT_abs_low3_MT3) AND (VT_abs_low3_MT2 > (MEAN(VT_abs_low3_MT1, VT_abs_low3_MT3, VT_abs_low3_MT4,
    VT_abs_low3_MT5, VT_abs_low3_FT1, VT_abs_low3_FT2, VT_abs_low3_FT3, VT_abs_low3_FT4, VT_abs_low3_FT5) + SD(VT_abs_low3_MT1,
    VT_abs_low3_MT3, VT_abs_low3_MT4, VT_abs_low3_MT5, VT_abs_low3_FT1, VT_abs_low3_FT2, VT_abs_low3_FT3, VT_abs_low3_FT4, VT_abs_low3_FT5))))
    VT_peak_low3_kons_MT2=1.
EXECUTE.

IF  ((VT_abs_low3_MT3 > VT_abs_low3_MT2) AND (VT_abs_low3_MT3 > VT_abs_low3_MT4) AND (VT_abs_low3_MT3 > (MEAN(VT_abs_low3_MT1, VT_abs_low3_MT2, VT_abs_low3_MT4,
    VT_abs_low3_MT5, VT_abs_low3_FT1, VT_abs_low3_FT2, VT_abs_low3_FT3, VT_abs_low3_FT4, VT_abs_low3_FT5) + SD(VT_abs_low3_MT1,
    VT_abs_low3_MT2, VT_abs_low3_MT4, VT_abs_low3_MT5, VT_abs_low3_FT1, VT_abs_low3_FT2, VT_abs_low3_FT3, VT_abs_low3_FT4, VT_abs_low3_FT5))))
    VT_peak_low3_kons_MT3=1.
EXECUTE.

IF  ((VT_abs_low3_MT4 > VT_abs_low3_MT3) AND (VT_abs_low3_MT4 > VT_abs_low3_MT5) AND (VT_abs_low3_MT4 > (MEAN(VT_abs_low3_MT1, VT_abs_low3_MT2, VT_abs_low3_MT3,
    VT_abs_low3_MT5, VT_abs_low3_FT1, VT_abs_low3_FT2, VT_abs_low3_FT3, VT_abs_low3_FT4, VT_abs_low3_FT5) + SD(VT_abs_low3_MT1,
    VT_abs_low3_MT2, VT_abs_low3_MT3, VT_abs_low3_MT5, VT_abs_low3_FT1, VT_abs_low3_FT2, VT_abs_low3_FT3, VT_abs_low3_FT4, VT_abs_low3_FT5))))
    VT_peak_low3_kons_MT4=1.
EXECUTE.

IF  ((VT_abs_low3_MT5 > VT_abs_low3_MT4) AND (VT_abs_low3_MT5 > (MEAN(VT_abs_low3_MT1, VT_abs_low3_MT2, VT_abs_low3_MT3,
    VT_abs_low3_MT4, VT_abs_low3_FT1, VT_abs_low3_FT2, VT_abs_low3_FT3, VT_abs_low3_FT4, VT_abs_low3_FT5) + SD(VT_abs_low3_MT1,
    VT_abs_low3_MT2, VT_abs_low3_MT3, VT_abs_low3_MT4, VT_abs_low3_FT1, VT_abs_low3_FT2, VT_abs_low3_FT3, VT_abs_low3_FT4, VT_abs_low3_FT5))))
    VT_peak_low3_kons_MT5=1.
EXECUTE.

IF  ((VT_abs_low3_FT1 > VT_abs_low3_FT2) AND (VT_abs_low3_FT1 > (MEAN(VT_abs_low3_MT1, VT_abs_low3_MT2, VT_abs_low3_MT3,
    VT_abs_low3_MT4, VT_abs_low3_MT5, VT_abs_low3_FT2, VT_abs_low3_FT3, VT_abs_low3_FT4, VT_abs_low3_FT5) + SD(VT_abs_low3_MT1,
    VT_abs_low3_MT2, VT_abs_low3_MT3, VT_abs_low3_MT4, VT_abs_low3_MT5, VT_abs_low3_FT2, VT_abs_low3_FT3, VT_abs_low3_FT4, VT_abs_low3_FT5))))
    VT_peak_low3_kons_FT1=1.
EXECUTE.

IF  ((VT_abs_low3_FT2 > VT_abs_low3_FT1) AND (VT_abs_low3_FT2 > VT_abs_low3_FT3) AND (VT_abs_low3_FT2 > (MEAN(VT_abs_low3_MT1, VT_abs_low3_MT2, VT_abs_low3_MT3,
    VT_abs_low3_MT4, VT_abs_low3_MT5, VT_abs_low3_FT1, VT_abs_low3_FT3, VT_abs_low3_FT4, VT_abs_low3_FT5) + SD(VT_abs_low3_MT1,
    VT_abs_low3_MT2, VT_abs_low3_MT3, VT_abs_low3_MT4, VT_abs_low3_MT5, VT_abs_low3_FT1, VT_abs_low3_FT3, VT_abs_low3_FT4, VT_abs_low3_FT5))))
    VT_peak_low3_kons_FT2=1.
EXECUTE.

IF  ((VT_abs_low3_FT3 > VT_abs_low3_FT2) AND (VT_abs_low3_FT3 > VT_abs_low3_FT4) AND (VT_abs_low3_FT3 > (MEAN(VT_abs_low3_MT1, VT_abs_low3_MT2, VT_abs_low3_MT3,
    VT_abs_low3_MT4, VT_abs_low3_MT5, VT_abs_low3_FT1, VT_abs_low3_FT2, VT_abs_low3_FT4, VT_abs_low3_FT5) + SD(VT_abs_low3_MT1,
    VT_abs_low3_MT2, VT_abs_low3_MT3, VT_abs_low3_MT4, VT_abs_low3_MT5, VT_abs_low3_FT1, VT_abs_low3_FT2, VT_abs_low3_FT4, VT_abs_low3_FT5))))
    VT_peak_low3_kons_FT3=1.
EXECUTE.

IF  ((VT_abs_low3_FT4 > VT_abs_low3_FT3) AND (VT_abs_low3_FT4 > VT_abs_low3_FT5) AND (VT_abs_low3_FT4 > (MEAN(VT_abs_low3_MT1, VT_abs_low3_MT2, VT_abs_low3_MT3,
    VT_abs_low3_MT4, VT_abs_low3_MT5, VT_abs_low3_FT1, VT_abs_low3_FT2, VT_abs_low3_FT3, VT_abs_low3_FT5) + SD(VT_abs_low3_MT1,
    VT_abs_low3_MT2, VT_abs_low3_MT3, VT_abs_low3_MT4, VT_abs_low3_MT5, VT_abs_low3_FT1, VT_abs_low3_FT2, VT_abs_low3_FT3, VT_abs_low3_FT5))))
    VT_peak_low3_kons_FT4=1.
EXECUTE.

IF  ((VT_abs_low3_FT5 > VT_abs_low3_FT4) AND (VT_abs_low3_FT5 > (MEAN(VT_abs_low3_MT1, VT_abs_low3_MT2, VT_abs_low3_MT3,
    VT_abs_low3_MT4, VT_abs_low3_MT5, VT_abs_low3_FT1, VT_abs_low3_FT2, VT_abs_low3_FT3, VT_abs_low3_FT4) + SD(VT_abs_low3_MT1,
    VT_abs_low3_MT2, VT_abs_low3_MT3, VT_abs_low3_MT4, VT_abs_low3_MT5, VT_abs_low3_FT1, VT_abs_low3_FT2, VT_abs_low3_FT3, VT_abs_low3_FT4))))
    VT_peak_low3_kons_FT5=1.
EXECUTE.

RECODE VT_peak_low3_kons_MT1 VT_peak_low3_kons_MT2 VT_peak_low3_kons_MT3 VT_peak_low3_kons_MT4
    VT_peak_low3_kons_MT5 VT_peak_low3_kons_FT1 VT_peak_low3_kons_FT2
    VT_peak_low3_kons_FT3 VT_peak_low3_kons_FT4 VT_peak_low3_kons_FT5(SYSMIS=0).
EXECUTE.

COUNT VT_peak_low3_kons_child=VT_peak_low3_kons_MT1 VT_peak_low3_kons_MT2 VT_peak_low3_kons_MT3
    VT_peak_low3_kons_FT1 VT_peak_low3_kons_FT2 VT_peak_low3_kons_FT3(1).
VARIABLE LABELS  VT_peak_low3_kons_child 'children-peaks low3 kons'.
EXECUTE.

COUNT VT_peak_low3_kons_girl=VT_peak_low3_kons_FT1 VT_peak_low3_kons_FT2 VT_peak_low3_kons_FT3(1).
VARIABLE LABELS  VT_peak_low3_kons_girl 'Mädchen-peaks low3 kons'.
EXECUTE.

COUNT VT_peak_low3_kons_boy=VT_peak_low3_kons_MT1 VT_peak_low3_kons_MT2 VT_peak_low3_kons_MT3(1).
VARIABLE LABELS  VT_peak_low3_kons_boy 'Jungen-peaks low3 kons'.
EXECUTE.

COUNT VT_peak_low3_kons_adult=VT_peak_low3_kons_MT4 VT_peak_low3_kons_MT5 VT_peak_low3_kons_FT4
    VT_peak_low3_kons_FT5(1).
VARIABLE LABELS  VT_peak_low3_kons_adult 'adult-peaks low3 kons'.
EXECUTE.

RECODE VT_peak_low3_kons_child VT_peak_low3_kons_adult VT_peak_low3_kons_girl VT_peak_low3_kons_boy(SYSMIS=0).
EXECUTE.


***progressive (1/2 SD)***
*by categories*

IF  ((VT_abs_low3_MT1 > VT_abs_low3_MT2) AND (VT_abs_low3_MT1 > (MEAN(VT_abs_low3_MT2, VT_abs_low3_MT3, VT_abs_low3_MT4,
    VT_abs_low3_MT5, VT_abs_low3_FT1, VT_abs_low3_FT2, VT_abs_low3_FT3, VT_abs_low3_FT4, VT_abs_low3_FT5) +
    (0.5 * SD(VT_abs_low3_MT2, VT_abs_low3_MT3, VT_abs_low3_MT4, VT_abs_low3_MT5, VT_abs_low3_FT1, VT_abs_low3_FT2,
    VT_abs_low3_FT3, VT_abs_low3_FT4, VT_abs_low3_FT5)))))
    VT_peak_low3_prog_MT1=1.
EXECUTE.

IF  ((VT_abs_low3_MT2 > VT_abs_low3_MT1) AND (VT_abs_low3_MT2 > VT_abs_low3_MT3) AND (VT_abs_low3_MT2 > (MEAN
    (VT_abs_low3_MT1, VT_abs_low3_MT3, VT_abs_low3_MT4, VT_abs_low3_MT5, VT_abs_low3_FT1, VT_abs_low3_FT2, VT_abs_low3_FT3,
    VT_abs_low3_FT4, VT_abs_low3_FT5) + (0.5 * SD(VT_abs_low3_MT1, VT_abs_low3_MT3, VT_abs_low3_MT4, VT_abs_low3_MT5,
    VT_abs_low3_FT1, VT_abs_low3_FT2, VT_abs_low3_FT3, VT_abs_low3_FT4, VT_abs_low3_FT5)))))
    VT_peak_low3_prog_MT2=1.
EXECUTE.

IF  ((VT_abs_low3_MT3 > VT_abs_low3_MT2) AND (VT_abs_low3_MT3 > VT_abs_low3_MT4) AND (VT_abs_low3_MT3 > (MEAN
    (VT_abs_low3_MT1, VT_abs_low3_MT2, VT_abs_low3_MT4, VT_abs_low3_MT5, VT_abs_low3_FT1, VT_abs_low3_FT2, VT_abs_low3_FT3,
    VT_abs_low3_FT4, VT_abs_low3_FT5) + (0.5 * SD(VT_abs_low3_MT1, VT_abs_low3_MT2, VT_abs_low3_MT4, VT_abs_low3_MT5,
    VT_abs_low3_FT1, VT_abs_low3_FT2, VT_abs_low3_FT3, VT_abs_low3_FT4, VT_abs_low3_FT5)))))
    VT_peak_low3_prog_MT3=1.
EXECUTE.

IF  ((VT_abs_low3_MT4 > VT_abs_low3_MT3) AND (VT_abs_low3_MT4 > VT_abs_low3_MT5) AND (VT_abs_low3_MT4 > (MEAN
    (VT_abs_low3_MT1, VT_abs_low3_MT2, VT_abs_low3_MT3, VT_abs_low3_MT5, VT_abs_low3_FT1, VT_abs_low3_FT2, VT_abs_low3_FT3,
    VT_abs_low3_FT4, VT_abs_low3_FT5) + (0.5 * SD(VT_abs_low3_MT1, VT_abs_low3_MT2, VT_abs_low3_MT3, VT_abs_low3_MT5,
    VT_abs_low3_FT1, VT_abs_low3_FT2, VT_abs_low3_FT3, VT_abs_low3_FT4, VT_abs_low3_FT5)))))
    VT_peak_low3_prog_MT4=1.
EXECUTE.

IF  ((VT_abs_low3_MT5 > VT_abs_low3_MT4) AND (VT_abs_low3_MT5 > (MEAN(VT_abs_low3_MT1, VT_abs_low3_MT2, VT_abs_low3_MT3,
    VT_abs_low3_MT4, VT_abs_low3_FT1, VT_abs_low3_FT2, VT_abs_low3_FT3, VT_abs_low3_FT4, VT_abs_low3_FT5) + (0.5 * SD
    (VT_abs_low3_MT1, VT_abs_low3_MT2, VT_abs_low3_MT3, VT_abs_low3_MT4, VT_abs_low3_FT1, VT_abs_low3_FT2, VT_abs_low3_FT3,
    VT_abs_low3_FT4, VT_abs_low3_FT5)))))
    VT_peak_low3_prog_MT5=1.
EXECUTE.

IF  ((VT_abs_low3_FT1 > VT_abs_low3_FT2) AND (VT_abs_low3_FT1 > (MEAN(VT_abs_low3_MT1, VT_abs_low3_MT2, VT_abs_low3_MT3,
    VT_abs_low3_MT4, VT_abs_low3_MT5, VT_abs_low3_FT2, VT_abs_low3_FT3, VT_abs_low3_FT4, VT_abs_low3_FT5) + (0.5 * SD
    (VT_abs_low3_MT1, VT_abs_low3_MT2, VT_abs_low3_MT3, VT_abs_low3_MT4, VT_abs_low3_MT5, VT_abs_low3_FT2, VT_abs_low3_FT3,
    VT_abs_low3_FT4, VT_abs_low3_FT5)))))
    VT_peak_low3_prog_FT1=1.
EXECUTE.

IF  ((VT_abs_low3_FT2 > VT_abs_low3_FT1) AND (VT_abs_low3_FT2 > VT_abs_low3_FT3) AND (VT_abs_low3_FT2 > (MEAN
    (VT_abs_low3_MT1, VT_abs_low3_MT2, VT_abs_low3_MT3, VT_abs_low3_MT4, VT_abs_low3_MT5, VT_abs_low3_FT1, VT_abs_low3_FT3,
    VT_abs_low3_FT4, VT_abs_low3_FT5) + (0.5 * SD(VT_abs_low3_MT1, VT_abs_low3_MT2, VT_abs_low3_MT3, VT_abs_low3_MT4,
    VT_abs_low3_MT5, VT_abs_low3_FT1, VT_abs_low3_FT3, VT_abs_low3_FT4, VT_abs_low3_FT5)))))
    VT_peak_low3_prog_FT2=1.
EXECUTE.

IF  ((VT_abs_low3_FT3 > VT_abs_low3_FT2) AND (VT_abs_low3_FT3 > VT_abs_low3_FT4) AND (VT_abs_low3_FT3 > (MEAN
    (VT_abs_low3_MT1, VT_abs_low3_MT2, VT_abs_low3_MT3, VT_abs_low3_MT4, VT_abs_low3_MT5, VT_abs_low3_FT1, VT_abs_low3_FT2,
    VT_abs_low3_FT4, VT_abs_low3_FT5) + (0.5 * SD(VT_abs_low3_MT1, VT_abs_low3_MT2, VT_abs_low3_MT3, VT_abs_low3_MT4,
    VT_abs_low3_MT5, VT_abs_low3_FT1, VT_abs_low3_FT2, VT_abs_low3_FT4, VT_abs_low3_FT5)))))
    VT_peak_low3_prog_FT3=1.
EXECUTE.

IF  ((VT_abs_low3_FT4 > VT_abs_low3_FT3) AND (VT_abs_low3_FT4 > VT_abs_low3_FT5) AND (VT_abs_low3_FT4 > (MEAN
    (VT_abs_low3_MT1, VT_abs_low3_MT2, VT_abs_low3_MT3, VT_abs_low3_MT4, VT_abs_low3_MT5, VT_abs_low3_FT1, VT_abs_low3_FT2,
    VT_abs_low3_FT3, VT_abs_low3_FT5) + (0.5 * SD(VT_abs_low3_MT1, VT_abs_low3_MT2, VT_abs_low3_MT3, VT_abs_low3_MT4,
    VT_abs_low3_MT5, VT_abs_low3_FT1, VT_abs_low3_FT2, VT_abs_low3_FT3, VT_abs_low3_FT5)))))
    VT_peak_low3_prog_FT4=1.
EXECUTE.

IF  ((VT_abs_low3_FT5 > VT_abs_low3_FT4) AND (VT_abs_low3_FT5 > (MEAN(VT_abs_low3_MT1, VT_abs_low3_MT2, VT_abs_low3_MT3,
    VT_abs_low3_MT4, VT_abs_low3_MT5, VT_abs_low3_FT1, VT_abs_low3_FT2, VT_abs_low3_FT3, VT_abs_low3_FT4) + (0.5 * SD
    (VT_abs_low3_MT1, VT_abs_low3_MT2, VT_abs_low3_MT3, VT_abs_low3_MT4, VT_abs_low3_MT5, VT_abs_low3_FT1, VT_abs_low3_FT2,
    VT_abs_low3_FT3, VT_abs_low3_FT4)))))
    VT_peak_low3_prog_FT5=1.
EXECUTE.

RECODE VT_peak_low3_prog_MT1 VT_peak_low3_prog_MT2 VT_peak_low3_prog_MT3 VT_peak_low3_prog_MT4
    VT_peak_low3_prog_MT5 VT_peak_low3_prog_FT1 VT_peak_low3_prog_FT2
    VT_peak_low3_prog_FT3 VT_peak_low3_prog_FT4 VT_peak_low3_prog_FT5(SYSMIS=0).
EXECUTE.

COUNT VT_peak_low3_prog_child=VT_peak_low3_prog_MT1 VT_peak_low3_prog_MT2 VT_peak_low3_prog_MT3
    VT_peak_low3_prog_FT1 VT_peak_low3_prog_FT2 VT_peak_low3_prog_FT3(1).
VARIABLE LABELS  VT_peak_low3_prog_child 'children-peaks low3 prog'.
EXECUTE.

COUNT VT_peak_low3_prog_girl=VT_peak_low3_prog_FT1 VT_peak_low3_prog_FT2 VT_peak_low3_prog_FT3(1).
VARIABLE LABELS  VT_peak_low3_prog_girl 'Mädchen-peaks low3 prog'.
EXECUTE.

COUNT VT_peak_low3_prog_boy=VT_peak_low3_prog_MT1 VT_peak_low3_prog_MT2 VT_peak_low3_prog_MT3(1).
VARIABLE LABELS  VT_peak_low3_prog_boy 'Jungen-peaks low3 prog'.
EXECUTE.

COUNT VT_peak_low3_prog_adult=VT_peak_low3_prog_MT4 VT_peak_low3_prog_MT5 VT_peak_low3_prog_FT4
    VT_peak_low3_prog_FT5(1).
VARIABLE LABELS  VT_peak_low3_prog_adult 'adult-peaks low3 prog'.
EXECUTE.


RECODE VT_peak_low3_prog_child VT_peak_low3_prog_adult VT_peak_low3_prog_girl VT_peak_low3_prog_boy(SYSMIS=0).
EXECUTE.

*relative values*

COMPUTE VT_peak_low3_prog_rel=VT_peak_low3_prog_child - VT_peak_low3_prog_adult.
EXECUTE.


***************scoring of the two pictures with the lowest VT (lower 50%)***************
*****absolute scoring*****
*MT1*

IF  (rating11_dur_clean = MAX(rating11_dur_clean, rating26_dur_clean, rating30_dur_clean,
    rating34_dur_clean) AND rating26_dur_clean = MAX(rating26_dur_clean, rating30_dur_clean,
    rating34_dur_clean)) VT_abs_low2_MT1=MEAN(rating30_dur_clean, rating34_dur_clean).
EXECUTE.

IF  (rating11_dur_clean = MAX(rating11_dur_clean, rating26_dur_clean, rating30_dur_clean,
    rating34_dur_clean) AND rating30_dur_clean = MAX(rating26_dur_clean, rating30_dur_clean,
    rating34_dur_clean)) VT_abs_low2_MT1=MEAN(rating26_dur_clean, rating34_dur_clean).
EXECUTE.

IF  (rating11_dur_clean = MAX(rating11_dur_clean, rating26_dur_clean, rating30_dur_clean,
    rating34_dur_clean) AND rating34_dur_clean = MAX(rating26_dur_clean, rating30_dur_clean,
    rating34_dur_clean)) VT_abs_low2_MT1=MEAN(rating26_dur_clean, rating30_dur_clean).
EXECUTE.

IF  (rating26_dur_clean = MAX(rating11_dur_clean, rating26_dur_clean, rating30_dur_clean,
    rating34_dur_clean) AND rating11_dur_clean = MAX(rating11_dur_clean, rating30_dur_clean,
    rating34_dur_clean)) VT_abs_low2_MT1=MEAN(rating30_dur_clean, rating34_dur_clean).
EXECUTE.

IF  (rating26_dur_clean = MAX(rating11_dur_clean, rating26_dur_clean, rating30_dur_clean,
    rating34_dur_clean) AND rating30_dur_clean = MAX(rating11_dur_clean, rating30_dur_clean,
    rating34_dur_clean)) VT_abs_low2_MT1=MEAN(rating11_dur_clean, rating34_dur_clean).
EXECUTE.

IF  (rating26_dur_clean = MAX(rating11_dur_clean, rating26_dur_clean, rating30_dur_clean,
    rating34_dur_clean) AND rating34_dur_clean = MAX(rating11_dur_clean, rating30_dur_clean,
    rating34_dur_clean)) VT_abs_low2_MT1=MEAN(rating11_dur_clean, rating30_dur_clean).
EXECUTE.

IF  (rating30_dur_clean = MAX(rating11_dur_clean, rating26_dur_clean, rating30_dur_clean,
    rating34_dur_clean) AND rating11_dur_clean = MAX(rating11_dur_clean, rating26_dur_clean,
    rating34_dur_clean)) VT_abs_low2_MT1=MEAN(rating26_dur_clean, rating34_dur_clean).
EXECUTE.

IF  (rating30_dur_clean = MAX(rating11_dur_clean, rating26_dur_clean, rating30_dur_clean,
    rating34_dur_clean) AND rating26_dur_clean = MAX(rating11_dur_clean, rating26_dur_clean,
    rating34_dur_clean)) VT_abs_low2_MT1=MEAN(rating11_dur_clean, rating34_dur_clean).
EXECUTE.

IF  (rating30_dur_clean = MAX(rating11_dur_clean, rating26_dur_clean, rating30_dur_clean,
    rating34_dur_clean) AND rating34_dur_clean = MAX(rating11_dur_clean, rating26_dur_clean,
    rating34_dur_clean)) VT_abs_low2_MT1=MEAN(rating11_dur_clean, rating26_dur_clean).
EXECUTE.

IF  (rating34_dur_clean = MAX(rating11_dur_clean, rating26_dur_clean, rating30_dur_clean,
    rating34_dur_clean) AND rating11_dur_clean = MAX(rating11_dur_clean, rating26_dur_clean,
    rating30_dur_clean)) VT_abs_low2_MT1=MEAN(rating26_dur_clean, rating30_dur_clean).
EXECUTE.

IF  (rating34_dur_clean = MAX(rating11_dur_clean, rating26_dur_clean, rating30_dur_clean,
    rating34_dur_clean) AND rating26_dur_clean = MAX(rating11_dur_clean, rating26_dur_clean,
    rating30_dur_clean)) VT_abs_low2_MT1=MEAN(rating11_dur_clean, rating30_dur_clean).
EXECUTE.

IF  (rating34_dur_clean = MAX(rating11_dur_clean, rating26_dur_clean, rating30_dur_clean,
    rating34_dur_clean) AND rating30_dur_clean = MAX(rating11_dur_clean, rating26_dur_clean,
    rating30_dur_clean)) VT_abs_low2_MT1=MEAN(rating11_dur_clean, rating26_dur_clean).
EXECUTE.

*MT2*

IF  (rating3_dur_clean = MAX(rating3_dur_clean, rating16_dur_clean, rating32_dur_clean,
    rating38_dur_clean) AND rating16_dur_clean = MAX(rating16_dur_clean, rating32_dur_clean,
    rating38_dur_clean)) VT_abs_low2_MT2=MEAN(rating32_dur_clean, rating38_dur_clean).
EXECUTE.

IF  (rating3_dur_clean = MAX(rating3_dur_clean, rating16_dur_clean, rating32_dur_clean,
    rating38_dur_clean) AND rating32_dur_clean = MAX(rating16_dur_clean, rating32_dur_clean,
    rating38_dur_clean)) VT_abs_low2_MT2=MEAN(rating16_dur_clean, rating38_dur_clean).
EXECUTE.

IF  (rating3_dur_clean = MAX(rating3_dur_clean, rating16_dur_clean, rating32_dur_clean,
    rating38_dur_clean) AND rating38_dur_clean = MAX(rating16_dur_clean, rating32_dur_clean,
    rating38_dur_clean)) VT_abs_low2_MT2=MEAN(rating16_dur_clean, rating32_dur_clean).
EXECUTE.

IF  (rating16_dur_clean = MAX(rating3_dur_clean, rating16_dur_clean, rating32_dur_clean,
    rating38_dur_clean) AND rating3_dur_clean = MAX(rating3_dur_clean, rating32_dur_clean,
    rating38_dur_clean)) VT_abs_low2_MT2=MEAN(rating32_dur_clean, rating38_dur_clean).
EXECUTE.

IF  (rating16_dur_clean = MAX(rating3_dur_clean, rating16_dur_clean, rating32_dur_clean,
    rating38_dur_clean) AND rating32_dur_clean = MAX(rating3_dur_clean, rating32_dur_clean,
    rating38_dur_clean)) VT_abs_low2_MT2=MEAN(rating3_dur_clean, rating38_dur_clean).
EXECUTE.

IF  (rating16_dur_clean = MAX(rating3_dur_clean, rating16_dur_clean, rating32_dur_clean,
    rating38_dur_clean) AND rating38_dur_clean = MAX(rating3_dur_clean, rating32_dur_clean,
    rating38_dur_clean)) VT_abs_low2_MT2=MEAN(rating3_dur_clean, rating32_dur_clean).
EXECUTE.

IF  (rating32_dur_clean = MAX(rating3_dur_clean, rating16_dur_clean, rating32_dur_clean,
    rating38_dur_clean) AND rating3_dur_clean = MAX(rating3_dur_clean, rating16_dur_clean,
    rating38_dur_clean)) VT_abs_low2_MT2=MEAN(rating16_dur_clean, rating38_dur_clean).
EXECUTE.

IF  (rating32_dur_clean = MAX(rating3_dur_clean, rating16_dur_clean, rating32_dur_clean,
    rating38_dur_clean) AND rating16_dur_clean = MAX(rating3_dur_clean, rating16_dur_clean,
    rating38_dur_clean)) VT_abs_low2_MT2=MEAN(rating3_dur_clean, rating38_dur_clean).
EXECUTE.

IF  (rating32_dur_clean = MAX(rating3_dur_clean, rating16_dur_clean, rating32_dur_clean,
    rating38_dur_clean) AND rating38_dur_clean = MAX(rating3_dur_clean, rating16_dur_clean,
    rating38_dur_clean)) VT_abs_low2_MT2=MEAN(rating3_dur_clean, rating16_dur_clean).
EXECUTE.

IF  (rating38_dur_clean = MAX(rating3_dur_clean, rating16_dur_clean, rating32_dur_clean,
    rating38_dur_clean) AND rating3_dur_clean = MAX(rating3_dur_clean, rating16_dur_clean,
    rating32_dur_clean)) VT_abs_low2_MT2=MEAN(rating16_dur_clean, rating32_dur_clean).
EXECUTE.

IF  (rating38_dur_clean = MAX(rating3_dur_clean, rating16_dur_clean, rating32_dur_clean,
    rating38_dur_clean) AND rating16_dur_clean = MAX(rating3_dur_clean, rating16_dur_clean,
    rating32_dur_clean)) VT_abs_low2_MT2=MEAN(rating3_dur_clean, rating32_dur_clean).
EXECUTE.

IF  (rating38_dur_clean = MAX(rating3_dur_clean, rating16_dur_clean, rating32_dur_clean,
    rating38_dur_clean) AND rating32_dur_clean = MAX(rating3_dur_clean, rating16_dur_clean,
    rating32_dur_clean)) VT_abs_low2_MT2=MEAN(rating3_dur_clean, rating16_dur_clean).
EXECUTE.

*MT3*

IF  (rating6_dur_clean = MAX(rating6_dur_clean, rating13_dur_clean, rating23_dur_clean,
    rating35_dur_clean) AND rating13_dur_clean = MAX(rating13_dur_clean, rating23_dur_clean,
    rating35_dur_clean)) VT_abs_low2_MT3=MEAN(rating23_dur_clean, rating35_dur_clean).
EXECUTE.

IF  (rating6_dur_clean = MAX(rating6_dur_clean, rating13_dur_clean, rating23_dur_clean,
    rating35_dur_clean) AND rating23_dur_clean = MAX(rating13_dur_clean, rating23_dur_clean,
    rating35_dur_clean)) VT_abs_low2_MT3=MEAN(rating13_dur_clean, rating35_dur_clean).
EXECUTE.

IF  (rating6_dur_clean = MAX(rating6_dur_clean, rating13_dur_clean, rating23_dur_clean,
    rating35_dur_clean) AND rating35_dur_clean = MAX(rating13_dur_clean, rating23_dur_clean,
    rating35_dur_clean)) VT_abs_low2_MT3=MEAN(rating13_dur_clean, rating23_dur_clean).
EXECUTE.

IF  (rating13_dur_clean = MAX(rating6_dur_clean, rating13_dur_clean, rating23_dur_clean,
    rating35_dur_clean) AND rating6_dur_clean = MAX(rating6_dur_clean, rating23_dur_clean,
    rating35_dur_clean)) VT_abs_low2_MT3=MEAN(rating23_dur_clean, rating35_dur_clean).
EXECUTE.

IF  (rating13_dur_clean = MAX(rating6_dur_clean, rating13_dur_clean, rating23_dur_clean,
    rating35_dur_clean) AND rating23_dur_clean = MAX(rating6_dur_clean, rating23_dur_clean,
    rating35_dur_clean)) VT_abs_low2_MT3=MEAN(rating6_dur_clean, rating35_dur_clean).
EXECUTE.

IF  (rating13_dur_clean = MAX(rating6_dur_clean, rating13_dur_clean, rating23_dur_clean,
    rating35_dur_clean) AND rating35_dur_clean = MAX(rating6_dur_clean, rating23_dur_clean,
    rating35_dur_clean)) VT_abs_low2_MT3=MEAN(rating6_dur_clean, rating23_dur_clean).
EXECUTE.

IF  (rating23_dur_clean = MAX(rating6_dur_clean, rating13_dur_clean, rating23_dur_clean,
    rating35_dur_clean) AND rating6_dur_clean = MAX(rating6_dur_clean, rating13_dur_clean,
    rating35_dur_clean)) VT_abs_low2_MT3=MEAN(rating13_dur_clean, rating35_dur_clean).
EXECUTE.

IF  (rating23_dur_clean = MAX(rating6_dur_clean, rating13_dur_clean, rating23_dur_clean,
    rating35_dur_clean) AND rating13_dur_clean = MAX(rating6_dur_clean, rating13_dur_clean,
    rating35_dur_clean)) VT_abs_low2_MT3=MEAN(rating6_dur_clean, rating35_dur_clean).
EXECUTE.

IF  (rating23_dur_clean = MAX(rating6_dur_clean, rating13_dur_clean, rating23_dur_clean,
    rating35_dur_clean) AND rating35_dur_clean = MAX(rating6_dur_clean, rating13_dur_clean,
    rating35_dur_clean)) VT_abs_low2_MT3=MEAN(rating6_dur_clean, rating13_dur_clean).
EXECUTE.

IF  (rating35_dur_clean = MAX(rating6_dur_clean, rating13_dur_clean, rating23_dur_clean,
    rating35_dur_clean) AND rating6_dur_clean = MAX(rating6_dur_clean, rating13_dur_clean,
    rating23_dur_clean)) VT_abs_low2_MT3=MEAN(rating13_dur_clean, rating23_dur_clean).
EXECUTE.

IF  (rating35_dur_clean = MAX(rating6_dur_clean, rating13_dur_clean, rating23_dur_clean,
    rating35_dur_clean) AND rating13_dur_clean = MAX(rating6_dur_clean, rating13_dur_clean,
    rating23_dur_clean)) VT_abs_low2_MT3=MEAN(rating6_dur_clean, rating23_dur_clean).
EXECUTE.

IF  (rating35_dur_clean = MAX(rating6_dur_clean, rating13_dur_clean, rating23_dur_clean,
    rating35_dur_clean) AND rating23_dur_clean = MAX(rating6_dur_clean, rating13_dur_clean,
    rating23_dur_clean)) VT_abs_low2_MT3=MEAN(rating6_dur_clean, rating13_dur_clean).
EXECUTE.

*MT4*

IF  (rating10_dur_clean = MAX(rating10_dur_clean, rating20_dur_clean, rating25_dur_clean,
    rating36_dur_clean) AND rating20_dur_clean = MAX(rating20_dur_clean, rating25_dur_clean,
    rating36_dur_clean)) VT_abs_low2_MT4=MEAN(rating25_dur_clean, rating36_dur_clean).
EXECUTE.

IF  (rating10_dur_clean = MAX(rating10_dur_clean, rating20_dur_clean, rating25_dur_clean,
    rating36_dur_clean) AND rating25_dur_clean = MAX(rating20_dur_clean, rating25_dur_clean,
    rating36_dur_clean)) VT_abs_low2_MT4=MEAN(rating20_dur_clean, rating36_dur_clean).
EXECUTE.

IF  (rating10_dur_clean = MAX(rating10_dur_clean, rating20_dur_clean, rating25_dur_clean,
    rating36_dur_clean) AND rating36_dur_clean = MAX(rating20_dur_clean, rating25_dur_clean,
    rating36_dur_clean)) VT_abs_low2_MT4=MEAN(rating20_dur_clean, rating25_dur_clean).
EXECUTE.

IF  (rating20_dur_clean = MAX(rating10_dur_clean, rating20_dur_clean, rating25_dur_clean,
    rating36_dur_clean) AND rating10_dur_clean = MAX(rating10_dur_clean, rating25_dur_clean,
    rating36_dur_clean)) VT_abs_low2_MT4=MEAN(rating25_dur_clean, rating36_dur_clean).
EXECUTE.

IF  (rating20_dur_clean = MAX(rating10_dur_clean, rating20_dur_clean, rating25_dur_clean,
    rating36_dur_clean) AND rating25_dur_clean = MAX(rating10_dur_clean, rating25_dur_clean,
    rating36_dur_clean)) VT_abs_low2_MT4=MEAN(rating10_dur_clean, rating36_dur_clean).
EXECUTE.

IF  (rating20_dur_clean = MAX(rating10_dur_clean, rating20_dur_clean, rating25_dur_clean,
    rating36_dur_clean) AND rating36_dur_clean = MAX(rating10_dur_clean, rating25_dur_clean,
    rating36_dur_clean)) VT_abs_low2_MT4=MEAN(rating10_dur_clean, rating25_dur_clean).
EXECUTE.

IF  (rating25_dur_clean = MAX(rating10_dur_clean, rating20_dur_clean, rating25_dur_clean,
    rating36_dur_clean) AND rating10_dur_clean = MAX(rating10_dur_clean, rating20_dur_clean,
    rating36_dur_clean)) VT_abs_low2_MT4=MEAN(rating20_dur_clean, rating36_dur_clean).
EXECUTE.

IF  (rating25_dur_clean = MAX(rating10_dur_clean, rating20_dur_clean, rating25_dur_clean,
    rating36_dur_clean) AND rating20_dur_clean = MAX(rating10_dur_clean, rating20_dur_clean,
    rating36_dur_clean)) VT_abs_low2_MT4=MEAN(rating10_dur_clean, rating36_dur_clean).
EXECUTE.

IF  (rating25_dur_clean = MAX(rating10_dur_clean, rating20_dur_clean, rating25_dur_clean,
    rating36_dur_clean) AND rating36_dur_clean = MAX(rating10_dur_clean, rating20_dur_clean,
    rating36_dur_clean)) VT_abs_low2_MT4=MEAN(rating10_dur_clean, rating20_dur_clean).
EXECUTE.

IF  (rating36_dur_clean = MAX(rating10_dur_clean, rating20_dur_clean, rating25_dur_clean,
    rating36_dur_clean) AND rating10_dur_clean = MAX(rating10_dur_clean, rating20_dur_clean,
    rating25_dur_clean)) VT_abs_low2_MT4=MEAN(rating20_dur_clean, rating25_dur_clean).
EXECUTE.

IF  (rating36_dur_clean = MAX(rating10_dur_clean, rating20_dur_clean, rating25_dur_clean,
    rating36_dur_clean) AND rating20_dur_clean = MAX(rating10_dur_clean, rating20_dur_clean,
    rating25_dur_clean)) VT_abs_low2_MT4=MEAN(rating10_dur_clean, rating25_dur_clean).
EXECUTE.

IF  (rating36_dur_clean = MAX(rating10_dur_clean, rating20_dur_clean, rating25_dur_clean,
    rating36_dur_clean) AND rating25_dur_clean = MAX(rating10_dur_clean, rating20_dur_clean,
    rating25_dur_clean)) VT_abs_low2_MT4=MEAN(rating10_dur_clean, rating20_dur_clean).
EXECUTE.

*MT5*

IF  (rating8_dur_clean = MAX(rating8_dur_clean, rating12_dur_clean, rating27_dur_clean,
    rating29_dur_clean) AND rating12_dur_clean = MAX(rating12_dur_clean, rating27_dur_clean,
    rating29_dur_clean)) VT_abs_low2_MT5=MEAN(rating27_dur_clean, rating29_dur_clean).
EXECUTE.

IF  (rating8_dur_clean = MAX(rating8_dur_clean, rating12_dur_clean, rating27_dur_clean,
    rating29_dur_clean) AND rating27_dur_clean = MAX(rating12_dur_clean, rating27_dur_clean,
    rating29_dur_clean)) VT_abs_low2_MT5=MEAN(rating12_dur_clean, rating29_dur_clean).
EXECUTE.

IF  (rating8_dur_clean = MAX(rating8_dur_clean, rating12_dur_clean, rating27_dur_clean,
    rating29_dur_clean) AND rating29_dur_clean = MAX(rating12_dur_clean, rating27_dur_clean,
    rating29_dur_clean)) VT_abs_low2_MT5=MEAN(rating12_dur_clean, rating27_dur_clean).
EXECUTE.

IF  (rating12_dur_clean = MAX(rating8_dur_clean, rating12_dur_clean, rating27_dur_clean,
    rating29_dur_clean) AND rating8_dur_clean = MAX(rating8_dur_clean, rating27_dur_clean,
    rating29_dur_clean)) VT_abs_low2_MT5=MEAN(rating27_dur_clean, rating29_dur_clean).
EXECUTE.

IF  (rating12_dur_clean = MAX(rating8_dur_clean, rating12_dur_clean, rating27_dur_clean,
    rating29_dur_clean) AND rating27_dur_clean = MAX(rating8_dur_clean, rating27_dur_clean,
    rating29_dur_clean)) VT_abs_low2_MT5=MEAN(rating8_dur_clean, rating29_dur_clean).
EXECUTE.

IF  (rating12_dur_clean = MAX(rating8_dur_clean, rating12_dur_clean, rating27_dur_clean,
    rating29_dur_clean) AND rating29_dur_clean = MAX(rating8_dur_clean, rating27_dur_clean,
    rating29_dur_clean)) VT_abs_low2_MT5=MEAN(rating8_dur_clean, rating27_dur_clean).
EXECUTE.

IF  (rating27_dur_clean = MAX(rating8_dur_clean, rating12_dur_clean, rating27_dur_clean,
    rating29_dur_clean) AND rating8_dur_clean = MAX(rating8_dur_clean, rating12_dur_clean,
    rating29_dur_clean)) VT_abs_low2_MT5=MEAN(rating12_dur_clean, rating29_dur_clean).
EXECUTE.

IF  (rating27_dur_clean = MAX(rating8_dur_clean, rating12_dur_clean, rating27_dur_clean,
    rating29_dur_clean) AND rating12_dur_clean = MAX(rating8_dur_clean, rating12_dur_clean,
    rating29_dur_clean)) VT_abs_low2_MT5=MEAN(rating8_dur_clean, rating29_dur_clean).
EXECUTE.

IF  (rating27_dur_clean = MAX(rating8_dur_clean, rating12_dur_clean, rating27_dur_clean,
    rating29_dur_clean) AND rating29_dur_clean = MAX(rating8_dur_clean, rating12_dur_clean,
    rating29_dur_clean)) VT_abs_low2_MT5=MEAN(rating8_dur_clean, rating12_dur_clean).
EXECUTE.

IF  (rating29_dur_clean = MAX(rating8_dur_clean, rating12_dur_clean, rating27_dur_clean,
    rating29_dur_clean) AND rating8_dur_clean = MAX(rating8_dur_clean, rating12_dur_clean,
    rating27_dur_clean)) VT_abs_low2_MT5=MEAN(rating12_dur_clean, rating27_dur_clean).
EXECUTE.

IF  (rating29_dur_clean = MAX(rating8_dur_clean, rating12_dur_clean, rating27_dur_clean,
    rating29_dur_clean) AND rating12_dur_clean = MAX(rating8_dur_clean, rating12_dur_clean,
    rating27_dur_clean)) VT_abs_low2_MT5=MEAN(rating8_dur_clean, rating27_dur_clean).
EXECUTE.

IF  (rating29_dur_clean = MAX(rating8_dur_clean, rating12_dur_clean, rating27_dur_clean,
    rating29_dur_clean) AND rating27_dur_clean = MAX(rating8_dur_clean, rating12_dur_clean,
    rating27_dur_clean)) VT_abs_low2_MT5=MEAN(rating8_dur_clean, rating12_dur_clean).
EXECUTE.

*FT1*

IF  (rating2_dur_clean = MAX(rating2_dur_clean, rating4_dur_clean, rating15_dur_clean,
    rating31_dur_clean) AND rating4_dur_clean = MAX(rating4_dur_clean, rating15_dur_clean,
    rating31_dur_clean)) VT_abs_low2_FT1=MEAN(rating15_dur_clean, rating31_dur_clean).
EXECUTE.

IF  (rating2_dur_clean = MAX(rating2_dur_clean, rating4_dur_clean, rating15_dur_clean,
    rating31_dur_clean) AND rating15_dur_clean = MAX(rating4_dur_clean, rating15_dur_clean,
    rating31_dur_clean)) VT_abs_low2_FT1=MEAN(rating4_dur_clean, rating31_dur_clean).
EXECUTE.

IF  (rating2_dur_clean = MAX(rating2_dur_clean, rating4_dur_clean, rating15_dur_clean,
    rating31_dur_clean) AND rating31_dur_clean = MAX(rating4_dur_clean, rating15_dur_clean,
    rating31_dur_clean)) VT_abs_low2_FT1=MEAN(rating4_dur_clean, rating15_dur_clean).
EXECUTE.

IF  (rating4_dur_clean = MAX(rating2_dur_clean, rating4_dur_clean, rating15_dur_clean,
    rating31_dur_clean) AND rating2_dur_clean = MAX(rating2_dur_clean, rating15_dur_clean,
    rating31_dur_clean)) VT_abs_low2_FT1=MEAN(rating15_dur_clean, rating31_dur_clean).
EXECUTE.

IF  (rating4_dur_clean = MAX(rating2_dur_clean, rating4_dur_clean, rating15_dur_clean,
    rating31_dur_clean) AND rating15_dur_clean = MAX(rating2_dur_clean, rating15_dur_clean,
    rating31_dur_clean)) VT_abs_low2_FT1=MEAN(rating2_dur_clean, rating31_dur_clean).
EXECUTE.

IF  (rating4_dur_clean = MAX(rating2_dur_clean, rating4_dur_clean, rating15_dur_clean,
    rating31_dur_clean) AND rating31_dur_clean = MAX(rating2_dur_clean, rating15_dur_clean,
    rating31_dur_clean)) VT_abs_low2_FT1=MEAN(rating2_dur_clean, rating15_dur_clean).
EXECUTE.

IF  (rating15_dur_clean = MAX(rating2_dur_clean, rating4_dur_clean, rating15_dur_clean,
    rating31_dur_clean) AND rating2_dur_clean = MAX(rating2_dur_clean, rating4_dur_clean,
    rating31_dur_clean)) VT_abs_low2_FT1=MEAN(rating4_dur_clean, rating31_dur_clean).
EXECUTE.

IF  (rating15_dur_clean = MAX(rating2_dur_clean, rating4_dur_clean, rating15_dur_clean,
    rating31_dur_clean) AND rating4_dur_clean = MAX(rating2_dur_clean, rating4_dur_clean,
    rating31_dur_clean)) VT_abs_low2_FT1=MEAN(rating2_dur_clean, rating31_dur_clean).
EXECUTE.

IF  (rating15_dur_clean = MAX(rating2_dur_clean, rating4_dur_clean, rating15_dur_clean,
    rating31_dur_clean) AND rating31_dur_clean = MAX(rating2_dur_clean, rating4_dur_clean,
    rating31_dur_clean)) VT_abs_low2_FT1=MEAN(rating2_dur_clean, rating4_dur_clean).
EXECUTE.

IF  (rating31_dur_clean = MAX(rating2_dur_clean, rating4_dur_clean, rating15_dur_clean,
    rating31_dur_clean) AND rating2_dur_clean = MAX(rating2_dur_clean, rating4_dur_clean,
    rating15_dur_clean)) VT_abs_low2_FT1=MEAN(rating4_dur_clean, rating15_dur_clean).
EXECUTE.

IF  (rating31_dur_clean = MAX(rating2_dur_clean, rating4_dur_clean, rating15_dur_clean,
    rating31_dur_clean) AND rating4_dur_clean = MAX(rating2_dur_clean, rating4_dur_clean,
    rating15_dur_clean)) VT_abs_low2_FT1=MEAN(rating2_dur_clean, rating15_dur_clean).
EXECUTE.

IF  (rating31_dur_clean = MAX(rating2_dur_clean, rating4_dur_clean, rating15_dur_clean,
    rating31_dur_clean) AND rating15_dur_clean = MAX(rating2_dur_clean, rating4_dur_clean,
    rating15_dur_clean)) VT_abs_low2_FT1=MEAN(rating2_dur_clean, rating4_dur_clean).
EXECUTE.


*FT2*

IF  (rating1_dur_clean = MAX(rating1_dur_clean, rating9_dur_clean, rating18_dur_clean,
    rating24_dur_clean) AND rating9_dur_clean = MAX(rating9_dur_clean, rating18_dur_clean,
    rating24_dur_clean)) VT_abs_low2_FT2=MEAN(rating18_dur_clean, rating24_dur_clean).
EXECUTE.

IF  (rating1_dur_clean = MAX(rating1_dur_clean, rating9_dur_clean, rating18_dur_clean,
    rating24_dur_clean) AND rating18_dur_clean = MAX(rating9_dur_clean, rating18_dur_clean,
    rating24_dur_clean)) VT_abs_low2_FT2=MEAN(rating9_dur_clean, rating24_dur_clean).
EXECUTE.

IF  (rating1_dur_clean = MAX(rating1_dur_clean, rating9_dur_clean, rating18_dur_clean,
    rating24_dur_clean) AND rating24_dur_clean = MAX(rating9_dur_clean, rating18_dur_clean,
    rating24_dur_clean)) VT_abs_low2_FT2=MEAN(rating9_dur_clean, rating18_dur_clean).
EXECUTE.

IF  (rating9_dur_clean = MAX(rating1_dur_clean, rating9_dur_clean, rating18_dur_clean,
    rating24_dur_clean) AND rating1_dur_clean = MAX(rating1_dur_clean, rating18_dur_clean,
    rating24_dur_clean)) VT_abs_low2_FT2=MEAN(rating18_dur_clean, rating24_dur_clean).
EXECUTE.

IF  (rating9_dur_clean = MAX(rating1_dur_clean, rating9_dur_clean, rating18_dur_clean,
    rating24_dur_clean) AND rating18_dur_clean = MAX(rating1_dur_clean, rating18_dur_clean,
    rating24_dur_clean)) VT_abs_low2_FT2=MEAN(rating1_dur_clean, rating24_dur_clean).
EXECUTE.

IF  (rating9_dur_clean = MAX(rating1_dur_clean, rating9_dur_clean, rating18_dur_clean,
    rating24_dur_clean) AND rating24_dur_clean = MAX(rating1_dur_clean, rating18_dur_clean,
    rating24_dur_clean)) VT_abs_low2_FT2=MEAN(rating1_dur_clean, rating18_dur_clean).
EXECUTE.

IF  (rating18_dur_clean = MAX(rating1_dur_clean, rating9_dur_clean, rating18_dur_clean,
    rating24_dur_clean) AND rating1_dur_clean = MAX(rating1_dur_clean, rating9_dur_clean,
    rating24_dur_clean)) VT_abs_low2_FT2=MEAN(rating9_dur_clean, rating24_dur_clean).
EXECUTE.

IF  (rating18_dur_clean = MAX(rating1_dur_clean, rating9_dur_clean, rating18_dur_clean,
    rating24_dur_clean) AND rating9_dur_clean = MAX(rating1_dur_clean, rating9_dur_clean,
    rating24_dur_clean)) VT_abs_low2_FT2=MEAN(rating1_dur_clean, rating24_dur_clean).
EXECUTE.

IF  (rating18_dur_clean = MAX(rating1_dur_clean, rating9_dur_clean, rating18_dur_clean,
    rating24_dur_clean) AND rating24_dur_clean = MAX(rating1_dur_clean, rating9_dur_clean,
    rating24_dur_clean)) VT_abs_low2_FT2=MEAN(rating1_dur_clean, rating9_dur_clean).
EXECUTE.

IF  (rating24_dur_clean = MAX(rating1_dur_clean, rating9_dur_clean, rating18_dur_clean,
    rating24_dur_clean) AND rating1_dur_clean = MAX(rating1_dur_clean, rating9_dur_clean,
    rating18_dur_clean)) VT_abs_low2_FT2=MEAN(rating9_dur_clean, rating18_dur_clean).
EXECUTE.

IF  (rating24_dur_clean = MAX(rating1_dur_clean, rating9_dur_clean, rating18_dur_clean,
    rating24_dur_clean) AND rating9_dur_clean = MAX(rating1_dur_clean, rating9_dur_clean,
    rating18_dur_clean)) VT_abs_low2_FT2=MEAN(rating1_dur_clean, rating18_dur_clean).
EXECUTE.

IF  (rating24_dur_clean = MAX(rating1_dur_clean, rating9_dur_clean, rating18_dur_clean,
    rating24_dur_clean) AND rating18_dur_clean = MAX(rating1_dur_clean, rating9_dur_clean,
    rating18_dur_clean)) VT_abs_low2_FT2=MEAN(rating1_dur_clean, rating9_dur_clean).
EXECUTE.

*FT3*

IF  (rating0_dur_clean = MAX(rating0_dur_clean, rating5_dur_clean, rating14_dur_clean,
    rating17_dur_clean) AND rating5_dur_clean = MAX(rating5_dur_clean, rating14_dur_clean,
    rating17_dur_clean)) VT_abs_low2_FT3=MEAN(rating14_dur_clean, rating17_dur_clean).
EXECUTE.

IF  (rating0_dur_clean = MAX(rating0_dur_clean, rating5_dur_clean, rating14_dur_clean,
    rating17_dur_clean) AND rating14_dur_clean = MAX(rating5_dur_clean, rating14_dur_clean,
    rating17_dur_clean)) VT_abs_low2_FT3=MEAN(rating5_dur_clean, rating17_dur_clean).
EXECUTE.

IF  (rating0_dur_clean = MAX(rating0_dur_clean, rating5_dur_clean, rating14_dur_clean,
    rating17_dur_clean) AND rating17_dur_clean = MAX(rating5_dur_clean, rating14_dur_clean,
    rating17_dur_clean)) VT_abs_low2_FT3=MEAN(rating5_dur_clean, rating14_dur_clean).
EXECUTE.

IF  (rating5_dur_clean = MAX(rating0_dur_clean, rating5_dur_clean, rating14_dur_clean,
    rating17_dur_clean) AND rating0_dur_clean = MAX(rating0_dur_clean, rating14_dur_clean,
    rating17_dur_clean)) VT_abs_low2_FT3=MEAN(rating14_dur_clean, rating17_dur_clean).
EXECUTE.

IF  (rating5_dur_clean = MAX(rating0_dur_clean, rating5_dur_clean, rating14_dur_clean,
    rating17_dur_clean) AND rating14_dur_clean = MAX(rating0_dur_clean, rating14_dur_clean,
    rating17_dur_clean)) VT_abs_low2_FT3=MEAN(rating0_dur_clean, rating17_dur_clean).
EXECUTE.

IF  (rating5_dur_clean = MAX(rating0_dur_clean, rating5_dur_clean, rating14_dur_clean,
    rating17_dur_clean) AND rating17_dur_clean = MAX(rating0_dur_clean, rating14_dur_clean,
    rating17_dur_clean)) VT_abs_low2_FT3=MEAN(rating0_dur_clean, rating14_dur_clean).
EXECUTE.

IF  (rating14_dur_clean = MAX(rating0_dur_clean, rating5_dur_clean, rating14_dur_clean,
    rating17_dur_clean) AND rating0_dur_clean = MAX(rating0_dur_clean, rating5_dur_clean,
    rating17_dur_clean)) VT_abs_low2_FT3=MEAN(rating5_dur_clean, rating17_dur_clean).
EXECUTE.

IF  (rating14_dur_clean = MAX(rating0_dur_clean, rating5_dur_clean, rating14_dur_clean,
    rating17_dur_clean) AND rating5_dur_clean = MAX(rating0_dur_clean, rating5_dur_clean,
    rating17_dur_clean)) VT_abs_low2_FT3=MEAN(rating0_dur_clean, rating17_dur_clean).
EXECUTE.

IF  (rating14_dur_clean = MAX(rating0_dur_clean, rating5_dur_clean, rating14_dur_clean,
    rating17_dur_clean) AND rating17_dur_clean = MAX(rating0_dur_clean, rating5_dur_clean,
    rating17_dur_clean)) VT_abs_low2_FT3=MEAN(rating0_dur_clean, rating5_dur_clean).
EXECUTE.

IF  (rating17_dur_clean = MAX(rating0_dur_clean, rating5_dur_clean, rating14_dur_clean,
    rating17_dur_clean) AND rating0_dur_clean = MAX(rating0_dur_clean, rating5_dur_clean,
    rating14_dur_clean)) VT_abs_low2_FT3=MEAN(rating5_dur_clean, rating14_dur_clean).
EXECUTE.

IF  (rating17_dur_clean = MAX(rating0_dur_clean, rating5_dur_clean, rating14_dur_clean,
    rating17_dur_clean) AND rating5_dur_clean = MAX(rating0_dur_clean, rating5_dur_clean,
    rating14_dur_clean)) VT_abs_low2_FT3=MEAN(rating0_dur_clean, rating14_dur_clean).
EXECUTE.

IF  (rating17_dur_clean = MAX(rating0_dur_clean, rating5_dur_clean, rating14_dur_clean,
    rating17_dur_clean) AND rating14_dur_clean = MAX(rating0_dur_clean, rating5_dur_clean,
    rating14_dur_clean)) VT_abs_low2_FT3=MEAN(rating0_dur_clean, rating5_dur_clean).
EXECUTE.

*FT4*

IF  (rating7_dur_clean = MAX(rating7_dur_clean, rating21_dur_clean, rating22_dur_clean,
    rating39_dur_clean) AND rating21_dur_clean = MAX(rating21_dur_clean, rating22_dur_clean,
    rating39_dur_clean)) VT_abs_low2_FT4=MEAN(rating22_dur_clean, rating39_dur_clean).
EXECUTE.

IF  (rating7_dur_clean = MAX(rating7_dur_clean, rating21_dur_clean, rating22_dur_clean,
    rating39_dur_clean) AND rating22_dur_clean = MAX(rating21_dur_clean, rating22_dur_clean,
    rating39_dur_clean)) VT_abs_low2_FT4=MEAN(rating21_dur_clean, rating39_dur_clean).
EXECUTE.

IF  (rating7_dur_clean = MAX(rating7_dur_clean, rating21_dur_clean, rating22_dur_clean,
    rating39_dur_clean) AND rating39_dur_clean = MAX(rating21_dur_clean, rating22_dur_clean,
    rating39_dur_clean)) VT_abs_low2_FT4=MEAN(rating21_dur_clean, rating22_dur_clean).
EXECUTE.

IF  (rating21_dur_clean = MAX(rating7_dur_clean, rating21_dur_clean, rating22_dur_clean,
    rating39_dur_clean) AND rating7_dur_clean = MAX(rating7_dur_clean, rating22_dur_clean,
    rating39_dur_clean)) VT_abs_low2_FT4=MEAN(rating22_dur_clean, rating39_dur_clean).
EXECUTE.

IF  (rating21_dur_clean = MAX(rating7_dur_clean, rating21_dur_clean, rating22_dur_clean,
    rating39_dur_clean) AND rating22_dur_clean = MAX(rating7_dur_clean, rating22_dur_clean,
    rating39_dur_clean)) VT_abs_low2_FT4=MEAN(rating7_dur_clean, rating39_dur_clean).
EXECUTE.

IF  (rating21_dur_clean = MAX(rating7_dur_clean, rating21_dur_clean, rating22_dur_clean,
    rating39_dur_clean) AND rating39_dur_clean = MAX(rating7_dur_clean, rating22_dur_clean,
    rating39_dur_clean)) VT_abs_low2_FT4=MEAN(rating7_dur_clean, rating22_dur_clean).
EXECUTE.

IF  (rating22_dur_clean = MAX(rating7_dur_clean, rating21_dur_clean, rating22_dur_clean,
    rating39_dur_clean) AND rating7_dur_clean = MAX(rating7_dur_clean, rating21_dur_clean,
    rating39_dur_clean)) VT_abs_low2_FT4=MEAN(rating21_dur_clean, rating39_dur_clean).
EXECUTE.

IF  (rating22_dur_clean = MAX(rating7_dur_clean, rating21_dur_clean, rating22_dur_clean,
    rating39_dur_clean) AND rating21_dur_clean = MAX(rating7_dur_clean, rating21_dur_clean,
    rating39_dur_clean)) VT_abs_low2_FT4=MEAN(rating7_dur_clean, rating39_dur_clean).
EXECUTE.

IF  (rating22_dur_clean = MAX(rating7_dur_clean, rating21_dur_clean, rating22_dur_clean,
    rating39_dur_clean) AND rating39_dur_clean = MAX(rating7_dur_clean, rating21_dur_clean,
    rating39_dur_clean)) VT_abs_low2_FT4=MEAN(rating7_dur_clean, rating21_dur_clean).
EXECUTE.

IF  (rating39_dur_clean = MAX(rating7_dur_clean, rating21_dur_clean, rating22_dur_clean,
    rating39_dur_clean) AND rating7_dur_clean = MAX(rating7_dur_clean, rating21_dur_clean,
    rating22_dur_clean)) VT_abs_low2_FT4=MEAN(rating21_dur_clean, rating22_dur_clean).
EXECUTE.

IF  (rating39_dur_clean = MAX(rating7_dur_clean, rating21_dur_clean, rating22_dur_clean,
    rating39_dur_clean) AND rating21_dur_clean = MAX(rating7_dur_clean, rating21_dur_clean,
    rating22_dur_clean)) VT_abs_low2_FT4=MEAN(rating7_dur_clean, rating22_dur_clean).
EXECUTE.

IF  (rating39_dur_clean = MAX(rating7_dur_clean, rating21_dur_clean, rating22_dur_clean,
    rating39_dur_clean) AND rating22_dur_clean = MAX(rating7_dur_clean, rating21_dur_clean,
    rating22_dur_clean)) VT_abs_low2_FT4=MEAN(rating7_dur_clean, rating21_dur_clean).
EXECUTE.

*FT5*

IF  (rating19_dur_clean = MAX(rating19_dur_clean, rating28_dur_clean, rating33_dur_clean,
    rating37_dur_clean) AND rating28_dur_clean = MAX(rating28_dur_clean, rating33_dur_clean,
    rating37_dur_clean)) VT_abs_low2_FT5=MEAN(rating33_dur_clean, rating37_dur_clean).
EXECUTE.

IF  (rating19_dur_clean = MAX(rating19_dur_clean, rating28_dur_clean, rating33_dur_clean,
    rating37_dur_clean) AND rating33_dur_clean = MAX(rating28_dur_clean, rating33_dur_clean,
    rating37_dur_clean)) VT_abs_low2_FT5=MEAN(rating28_dur_clean, rating37_dur_clean).
EXECUTE.

IF  (rating19_dur_clean = MAX(rating19_dur_clean, rating28_dur_clean, rating33_dur_clean,
    rating37_dur_clean) AND rating37_dur_clean = MAX(rating28_dur_clean, rating33_dur_clean,
    rating37_dur_clean)) VT_abs_low2_FT5=MEAN(rating28_dur_clean, rating33_dur_clean).
EXECUTE.

IF  (rating28_dur_clean = MAX(rating19_dur_clean, rating28_dur_clean, rating33_dur_clean,
    rating37_dur_clean) AND rating19_dur_clean = MAX(rating19_dur_clean, rating33_dur_clean,
    rating37_dur_clean)) VT_abs_low2_FT5=MEAN(rating33_dur_clean, rating37_dur_clean).
EXECUTE.

IF  (rating28_dur_clean = MAX(rating19_dur_clean, rating28_dur_clean, rating33_dur_clean,
    rating37_dur_clean) AND rating33_dur_clean = MAX(rating19_dur_clean, rating33_dur_clean,
    rating37_dur_clean)) VT_abs_low2_FT5=MEAN(rating19_dur_clean, rating37_dur_clean).
EXECUTE.

IF  (rating28_dur_clean = MAX(rating19_dur_clean, rating28_dur_clean, rating33_dur_clean,
    rating37_dur_clean) AND rating37_dur_clean = MAX(rating19_dur_clean, rating33_dur_clean,
    rating37_dur_clean)) VT_abs_low2_FT5=MEAN(rating19_dur_clean, rating33_dur_clean).
EXECUTE.

IF  (rating33_dur_clean = MAX(rating19_dur_clean, rating28_dur_clean, rating33_dur_clean,
    rating37_dur_clean) AND rating19_dur_clean = MAX(rating19_dur_clean, rating28_dur_clean,
    rating37_dur_clean)) VT_abs_low2_FT5=MEAN(rating28_dur_clean, rating37_dur_clean).
EXECUTE.

IF  (rating33_dur_clean = MAX(rating19_dur_clean, rating28_dur_clean, rating33_dur_clean,
    rating37_dur_clean) AND rating28_dur_clean = MAX(rating19_dur_clean, rating28_dur_clean,
    rating37_dur_clean)) VT_abs_low2_FT5=MEAN(rating19_dur_clean, rating37_dur_clean).
EXECUTE.

IF  (rating33_dur_clean = MAX(rating19_dur_clean, rating28_dur_clean, rating33_dur_clean,
    rating37_dur_clean) AND rating37_dur_clean = MAX(rating19_dur_clean, rating28_dur_clean,
    rating37_dur_clean)) VT_abs_low2_FT5=MEAN(rating19_dur_clean, rating28_dur_clean).
EXECUTE.

IF  (rating37_dur_clean = MAX(rating19_dur_clean, rating28_dur_clean, rating33_dur_clean,
    rating37_dur_clean) AND rating19_dur_clean = MAX(rating19_dur_clean, rating28_dur_clean,
    rating33_dur_clean)) VT_abs_low2_FT5=MEAN(rating28_dur_clean, rating33_dur_clean).
EXECUTE.

IF  (rating37_dur_clean = MAX(rating19_dur_clean, rating28_dur_clean, rating33_dur_clean,
    rating37_dur_clean) AND rating28_dur_clean = MAX(rating19_dur_clean, rating28_dur_clean,
    rating33_dur_clean)) VT_abs_low2_FT5=MEAN(rating19_dur_clean, rating33_dur_clean).
EXECUTE.

IF  (rating37_dur_clean = MAX(rating19_dur_clean, rating28_dur_clean, rating33_dur_clean,
    rating37_dur_clean) AND rating33_dur_clean = MAX(rating19_dur_clean, rating28_dur_clean,
    rating33_dur_clean)) VT_abs_low2_FT5=MEAN(rating19_dur_clean, rating28_dur_clean).
EXECUTE.


*****relative scoring*****

COMPUTE VT_rel_D_low2=(MEAN(VT_abs_low2_MT1, VT_abs_low2_MT2, VT_abs_low2_MT3, VT_abs_low2_FT1, VT_abs_low2_FT2,
    VT_abs_low2_FT3)) - (MEAN(VT_abs_low2_MT4, VT_abs_low2_MT5, VT_abs_low2_FT4, VT_abs_low2_FT5)).
EXECUTE.


*****peak detection*****
***conservative (SD)***
*by categories*

IF  ((VT_abs_low2_MT1 > VT_abs_low2_MT2) AND (VT_abs_low2_MT1 > (MEAN(VT_abs_low2_MT2, VT_abs_low2_MT3, VT_abs_low2_MT4,
    VT_abs_low2_MT5, VT_abs_low2_FT1, VT_abs_low2_FT2, VT_abs_low2_FT3, VT_abs_low2_FT4, VT_abs_low2_FT5) + SD(VT_abs_low2_MT2,
    VT_abs_low2_MT3, VT_abs_low2_MT4, VT_abs_low2_MT5, VT_abs_low2_FT1, VT_abs_low2_FT2, VT_abs_low2_FT3, VT_abs_low2_FT4, VT_abs_low2_FT5))))
    VT_peak_low2_kons_MT1=1.
EXECUTE.

IF  ((VT_abs_low2_MT2 > VT_abs_low2_MT1) AND (VT_abs_low2_MT2 > VT_abs_low2_MT3) AND (VT_abs_low2_MT2 > (MEAN(VT_abs_low2_MT1, VT_abs_low2_MT3, VT_abs_low2_MT4,
    VT_abs_low2_MT5, VT_abs_low2_FT1, VT_abs_low2_FT2, VT_abs_low2_FT3, VT_abs_low2_FT4, VT_abs_low2_FT5) + SD(VT_abs_low2_MT1,
    VT_abs_low2_MT3, VT_abs_low2_MT4, VT_abs_low2_MT5, VT_abs_low2_FT1, VT_abs_low2_FT2, VT_abs_low2_FT3, VT_abs_low2_FT4, VT_abs_low2_FT5))))
    VT_peak_low2_kons_MT2=1.
EXECUTE.

IF  ((VT_abs_low2_MT3 > VT_abs_low2_MT2) AND (VT_abs_low2_MT3 > VT_abs_low2_MT4) AND (VT_abs_low2_MT3 > (MEAN(VT_abs_low2_MT1, VT_abs_low2_MT2, VT_abs_low2_MT4,
    VT_abs_low2_MT5, VT_abs_low2_FT1, VT_abs_low2_FT2, VT_abs_low2_FT3, VT_abs_low2_FT4, VT_abs_low2_FT5) + SD(VT_abs_low2_MT1,
    VT_abs_low2_MT2, VT_abs_low2_MT4, VT_abs_low2_MT5, VT_abs_low2_FT1, VT_abs_low2_FT2, VT_abs_low2_FT3, VT_abs_low2_FT4, VT_abs_low2_FT5))))
    VT_peak_low2_kons_MT3=1.
EXECUTE.

IF  ((VT_abs_low2_MT4 > VT_abs_low2_MT3) AND (VT_abs_low2_MT4 > VT_abs_low2_MT5) AND (VT_abs_low2_MT4 > (MEAN(VT_abs_low2_MT1, VT_abs_low2_MT2, VT_abs_low2_MT3,
    VT_abs_low2_MT5, VT_abs_low2_FT1, VT_abs_low2_FT2, VT_abs_low2_FT3, VT_abs_low2_FT4, VT_abs_low2_FT5) + SD(VT_abs_low2_MT1,
    VT_abs_low2_MT2, VT_abs_low2_MT3, VT_abs_low2_MT5, VT_abs_low2_FT1, VT_abs_low2_FT2, VT_abs_low2_FT3, VT_abs_low2_FT4, VT_abs_low2_FT5))))
    VT_peak_low2_kons_MT4=1.
EXECUTE.

IF  ((VT_abs_low2_MT5 > VT_abs_low2_MT4) AND (VT_abs_low2_MT5 > (MEAN(VT_abs_low2_MT1, VT_abs_low2_MT2, VT_abs_low2_MT3,
    VT_abs_low2_MT4, VT_abs_low2_FT1, VT_abs_low2_FT2, VT_abs_low2_FT3, VT_abs_low2_FT4, VT_abs_low2_FT5) + SD(VT_abs_low2_MT1,
    VT_abs_low2_MT2, VT_abs_low2_MT3, VT_abs_low2_MT4, VT_abs_low2_FT1, VT_abs_low2_FT2, VT_abs_low2_FT3, VT_abs_low2_FT4, VT_abs_low2_FT5))))
    VT_peak_low2_kons_MT5=1.
EXECUTE.

IF  ((VT_abs_low2_FT1 > VT_abs_low2_FT2) AND (VT_abs_low2_FT1 > (MEAN(VT_abs_low2_MT1, VT_abs_low2_MT2, VT_abs_low2_MT3,
    VT_abs_low2_MT4, VT_abs_low2_MT5, VT_abs_low2_FT2, VT_abs_low2_FT3, VT_abs_low2_FT4, VT_abs_low2_FT5) + SD(VT_abs_low2_MT1,
    VT_abs_low2_MT2, VT_abs_low2_MT3, VT_abs_low2_MT4, VT_abs_low2_MT5, VT_abs_low2_FT2, VT_abs_low2_FT3, VT_abs_low2_FT4, VT_abs_low2_FT5))))
    VT_peak_low2_kons_FT1=1.
EXECUTE.

IF  ((VT_abs_low2_FT2 > VT_abs_low2_FT1) AND (VT_abs_low2_FT2 > VT_abs_low2_FT3) AND (VT_abs_low2_FT2 > (MEAN(VT_abs_low2_MT1, VT_abs_low2_MT2, VT_abs_low2_MT3,
    VT_abs_low2_MT4, VT_abs_low2_MT5, VT_abs_low2_FT1, VT_abs_low2_FT3, VT_abs_low2_FT4, VT_abs_low2_FT5) + SD(VT_abs_low2_MT1,
    VT_abs_low2_MT2, VT_abs_low2_MT3, VT_abs_low2_MT4, VT_abs_low2_MT5, VT_abs_low2_FT1, VT_abs_low2_FT3, VT_abs_low2_FT4, VT_abs_low2_FT5))))
    VT_peak_low2_kons_FT2=1.
EXECUTE.

IF  ((VT_abs_low2_FT3 > VT_abs_low2_FT2) AND (VT_abs_low2_FT3 > VT_abs_low2_FT4) AND (VT_abs_low2_FT3 > (MEAN(VT_abs_low2_MT1, VT_abs_low2_MT2, VT_abs_low2_MT3,
    VT_abs_low2_MT4, VT_abs_low2_MT5, VT_abs_low2_FT1, VT_abs_low2_FT2, VT_abs_low2_FT4, VT_abs_low2_FT5) + SD(VT_abs_low2_MT1,
    VT_abs_low2_MT2, VT_abs_low2_MT3, VT_abs_low2_MT4, VT_abs_low2_MT5, VT_abs_low2_FT1, VT_abs_low2_FT2, VT_abs_low2_FT4, VT_abs_low2_FT5))))
    VT_peak_low2_kons_FT3=1.
EXECUTE.

IF  ((VT_abs_low2_FT4 > VT_abs_low2_FT3) AND (VT_abs_low2_FT4 > VT_abs_low2_FT5) AND (VT_abs_low2_FT4 > (MEAN(VT_abs_low2_MT1, VT_abs_low2_MT2, VT_abs_low2_MT3,
    VT_abs_low2_MT4, VT_abs_low2_MT5, VT_abs_low2_FT1, VT_abs_low2_FT2, VT_abs_low2_FT3, VT_abs_low2_FT5) + SD(VT_abs_low2_MT1,
    VT_abs_low2_MT2, VT_abs_low2_MT3, VT_abs_low2_MT4, VT_abs_low2_MT5, VT_abs_low2_FT1, VT_abs_low2_FT2, VT_abs_low2_FT3, VT_abs_low2_FT5))))
    VT_peak_low2_kons_FT4=1.
EXECUTE.

IF  ((VT_abs_low2_FT5 > VT_abs_low2_FT4) AND (VT_abs_low2_FT5 > (MEAN(VT_abs_low2_MT1, VT_abs_low2_MT2, VT_abs_low2_MT3,
    VT_abs_low2_MT4, VT_abs_low2_MT5, VT_abs_low2_FT1, VT_abs_low2_FT2, VT_abs_low2_FT3, VT_abs_low2_FT4) + SD(VT_abs_low2_MT1,
    VT_abs_low2_MT2, VT_abs_low2_MT3, VT_abs_low2_MT4, VT_abs_low2_MT5, VT_abs_low2_FT1, VT_abs_low2_FT2, VT_abs_low2_FT3, VT_abs_low2_FT4))))
    VT_peak_low2_kons_FT5=1.
EXECUTE.

COUNT VT_peak_low2_kons_child=VT_peak_low2_kons_MT1 VT_peak_low2_kons_MT2 VT_peak_low2_kons_MT3
    VT_peak_low2_kons_FT1 VT_peak_low2_kons_FT2 VT_peak_low2_kons_FT3(1).
VARIABLE LABELS  VT_peak_low2_kons_child 'children-peaks low2 kons'.
EXECUTE.

COUNT VT_peak_low2_kons_girl=VT_peak_low2_kons_FT1 VT_peak_low2_kons_FT2 VT_peak_low2_kons_FT3(1).
VARIABLE LABELS  VT_peak_low2_kons_girl 'Mädchen-peaks low2 kons'.
EXECUTE.

COUNT VT_peak_low2_kons_boy=VT_peak_low2_kons_MT1 VT_peak_low2_kons_MT2 VT_peak_low2_kons_MT3(1).
VARIABLE LABELS  VT_peak_low2_kons_boy 'Jungen-peaks low2 kons'.
EXECUTE.

COUNT VT_peak_low2_kons_adult=VT_peak_low2_kons_MT4 VT_peak_low2_kons_MT5 VT_peak_low2_kons_FT4
    VT_peak_low2_kons_FT5(1).
VARIABLE LABELS  VT_peak_low2_kons_adult 'adult-peaks low2 kons'.
EXECUTE.

RECODE VT_peak_low2_kons_MT1 VT_peak_low2_kons_MT2 VT_peak_low2_kons_MT3 VT_peak_low2_kons_MT4
    VT_peak_low2_kons_MT5 VT_peak_low2_kons_FT1 VT_peak_low2_kons_FT2 VT_peak_low2_kons_FT3
    VT_peak_low2_kons_FT4 VT_peak_low2_kons_FT5 VT_peak_low2_kons_child VT_peak_low2_kons_adult
    VT_peak_low2_kons_girl VT_peak_low2_kons_boy (SYSMIS=0).
EXECUTE.


***progressive (1/2 SD)***
*by categories*

IF  ((VT_abs_low2_MT1 > VT_abs_low2_MT2) AND (VT_abs_low2_MT1 > (MEAN(VT_abs_low2_MT2, VT_abs_low2_MT3, VT_abs_low2_MT4,
    VT_abs_low2_MT5, VT_abs_low2_FT1, VT_abs_low2_FT2, VT_abs_low2_FT3, VT_abs_low2_FT4, VT_abs_low2_FT5) + (0.5 * SD(VT_abs_low2_MT2,
    VT_abs_low2_MT3, VT_abs_low2_MT4, VT_abs_low2_MT5, VT_abs_low2_FT1, VT_abs_low2_FT2, VT_abs_low2_FT3, VT_abs_low2_FT4, VT_abs_low2_FT5)))))
    VT_peak_low2_prog_MT1=1.
EXECUTE.

IF  ((VT_abs_low2_MT2 > VT_abs_low2_MT1) AND (VT_abs_low2_MT2 > VT_abs_low2_MT3) AND (VT_abs_low2_MT2 > (MEAN(VT_abs_low2_MT1, VT_abs_low2_MT3, VT_abs_low2_MT4,
    VT_abs_low2_MT5, VT_abs_low2_FT1, VT_abs_low2_FT2, VT_abs_low2_FT3, VT_abs_low2_FT4, VT_abs_low2_FT5) + (0.5 * SD(VT_abs_low2_MT1,
    VT_abs_low2_MT3, VT_abs_low2_MT4, VT_abs_low2_MT5, VT_abs_low2_FT1, VT_abs_low2_FT2, VT_abs_low2_FT3, VT_abs_low2_FT4, VT_abs_low2_FT5)))))
    VT_peak_low2_prog_MT2=1.
EXECUTE.

IF  ((VT_abs_low2_MT3 > VT_abs_low2_MT2) AND (VT_abs_low2_MT3 > VT_abs_low2_MT4) AND (VT_abs_low2_MT3 > (MEAN(VT_abs_low2_MT1, VT_abs_low2_MT2, VT_abs_low2_MT4,
    VT_abs_low2_MT5, VT_abs_low2_FT1, VT_abs_low2_FT2, VT_abs_low2_FT3, VT_abs_low2_FT4, VT_abs_low2_FT5) + (0.5 * SD(VT_abs_low2_MT1,
    VT_abs_low2_MT2, VT_abs_low2_MT4, VT_abs_low2_MT5, VT_abs_low2_FT1, VT_abs_low2_FT2, VT_abs_low2_FT3, VT_abs_low2_FT4, VT_abs_low2_FT5)))))
    VT_peak_low2_prog_MT3=1.
EXECUTE.

IF  ((VT_abs_low2_MT4 > VT_abs_low2_MT3) AND (VT_abs_low2_MT4 > VT_abs_low2_MT5) AND (VT_abs_low2_MT4 > (MEAN(VT_abs_low2_MT1, VT_abs_low2_MT2, VT_abs_low2_MT3,
    VT_abs_low2_MT5, VT_abs_low2_FT1, VT_abs_low2_FT2, VT_abs_low2_FT3, VT_abs_low2_FT4, VT_abs_low2_FT5) + (0.5 * SD(VT_abs_low2_MT1,
    VT_abs_low2_MT2, VT_abs_low2_MT3, VT_abs_low2_MT5, VT_abs_low2_FT1, VT_abs_low2_FT2, VT_abs_low2_FT3, VT_abs_low2_FT4, VT_abs_low2_FT5)))))
    VT_peak_low2_prog_MT4=1.
EXECUTE.

IF  ((VT_abs_low2_MT5 > VT_abs_low2_MT4) AND (VT_abs_low2_MT5 > (MEAN(VT_abs_low2_MT1, VT_abs_low2_MT2, VT_abs_low2_MT3,
    VT_abs_low2_MT4, VT_abs_low2_FT1, VT_abs_low2_FT2, VT_abs_low2_FT3, VT_abs_low2_FT4, VT_abs_low2_FT5) + (0.5 * SD(VT_abs_low2_MT1,
    VT_abs_low2_MT2, VT_abs_low2_MT3, VT_abs_low2_MT4, VT_abs_low2_FT1, VT_abs_low2_FT2, VT_abs_low2_FT3, VT_abs_low2_FT4, VT_abs_low2_FT5)))))
    VT_peak_low2_prog_MT5=1.
EXECUTE.

IF  ((VT_abs_low2_FT1 > VT_abs_low2_FT2) AND (VT_abs_low2_FT1 > (MEAN(VT_abs_low2_MT1, VT_abs_low2_MT2, VT_abs_low2_MT3,
    VT_abs_low2_MT4, VT_abs_low2_MT5, VT_abs_low2_FT2, VT_abs_low2_FT3, VT_abs_low2_FT4, VT_abs_low2_FT5) + (0.5 * SD(VT_abs_low2_MT1,
    VT_abs_low2_MT2, VT_abs_low2_MT3, VT_abs_low2_MT4, VT_abs_low2_MT5, VT_abs_low2_FT2, VT_abs_low2_FT3, VT_abs_low2_FT4, VT_abs_low2_FT5)))))
    VT_peak_low2_prog_FT1=1.
EXECUTE.

IF  ((VT_abs_low2_FT2 > VT_abs_low2_FT1) AND (VT_abs_low2_FT2 > VT_abs_low2_FT3) AND (VT_abs_low2_FT2 > (MEAN(VT_abs_low2_MT1, VT_abs_low2_MT2, VT_abs_low2_MT3,
    VT_abs_low2_MT4, VT_abs_low2_MT5, VT_abs_low2_FT1, VT_abs_low2_FT3, VT_abs_low2_FT4, VT_abs_low2_FT5) + (0.5 * SD(VT_abs_low2_MT1,
    VT_abs_low2_MT2, VT_abs_low2_MT3, VT_abs_low2_MT4, VT_abs_low2_MT5, VT_abs_low2_FT1, VT_abs_low2_FT3, VT_abs_low2_FT4, VT_abs_low2_FT5)))))
    VT_peak_low2_prog_FT2=1.
EXECUTE.

IF  ((VT_abs_low2_FT3 > VT_abs_low2_FT2) AND (VT_abs_low2_FT3 > VT_abs_low2_FT4) AND (VT_abs_low2_FT3 > (MEAN(VT_abs_low2_MT1, VT_abs_low2_MT2, VT_abs_low2_MT3,
    VT_abs_low2_MT4, VT_abs_low2_MT5, VT_abs_low2_FT1, VT_abs_low2_FT2, VT_abs_low2_FT4, VT_abs_low2_FT5) + (0.5 * SD(VT_abs_low2_MT1,
    VT_abs_low2_MT2, VT_abs_low2_MT3, VT_abs_low2_MT4, VT_abs_low2_MT5, VT_abs_low2_FT1, VT_abs_low2_FT2, VT_abs_low2_FT4, VT_abs_low2_FT5)))))
    VT_peak_low2_prog_FT3=1.
EXECUTE.

IF  ((VT_abs_low2_FT4 > VT_abs_low2_FT3) AND (VT_abs_low2_FT4 > VT_abs_low2_FT5) AND (VT_abs_low2_FT4 > (MEAN(VT_abs_low2_MT1, VT_abs_low2_MT2, VT_abs_low2_MT3,
    VT_abs_low2_MT4, VT_abs_low2_MT5, VT_abs_low2_FT1, VT_abs_low2_FT2, VT_abs_low2_FT3, VT_abs_low2_FT5) + (0.5 * SD(VT_abs_low2_MT1,
    VT_abs_low2_MT2, VT_abs_low2_MT3, VT_abs_low2_MT4, VT_abs_low2_MT5, VT_abs_low2_FT1, VT_abs_low2_FT2, VT_abs_low2_FT3, VT_abs_low2_FT5)))))
    VT_peak_low2_prog_FT4=1.
EXECUTE.

IF  ((VT_abs_low2_FT5 > VT_abs_low2_FT4) AND (VT_abs_low2_FT5 > (MEAN(VT_abs_low2_MT1, VT_abs_low2_MT2, VT_abs_low2_MT3,
    VT_abs_low2_MT4, VT_abs_low2_MT5, VT_abs_low2_FT1, VT_abs_low2_FT2, VT_abs_low2_FT3, VT_abs_low2_FT4) + (0.5 * SD(VT_abs_low2_MT1,
    VT_abs_low2_MT2, VT_abs_low2_MT3, VT_abs_low2_MT4, VT_abs_low2_MT5, VT_abs_low2_FT1, VT_abs_low2_FT2, VT_abs_low2_FT3, VT_abs_low2_FT4)))))
    VT_peak_low2_prog_FT5=1.
EXECUTE.

COUNT VT_peak_low2_prog_child=VT_peak_low2_prog_MT1 VT_peak_low2_prog_MT2 VT_peak_low2_prog_MT3
    VT_peak_low2_prog_FT1 VT_peak_low2_prog_FT2 VT_peak_low2_prog_FT3(1).
VARIABLE LABELS  VT_peak_low2_prog_child 'children-peaks low2 prog'.
EXECUTE.

COUNT VT_peak_low2_prog_girl=VT_peak_low2_prog_FT1 VT_peak_low2_prog_FT2 VT_peak_low2_prog_FT3(1).
VARIABLE LABELS  VT_peak_low2_prog_girl 'Mädchen-peaks low2 prog'.
EXECUTE.

COUNT VT_peak_low2_prog_boy=VT_peak_low2_prog_MT1 VT_peak_low2_prog_MT2 VT_peak_low2_prog_MT3 (1).
VARIABLE LABELS  VT_peak_low2_prog_boy 'Jungen-peaks low2 prog'.
EXECUTE.

COUNT VT_peak_low2_prog_adult=VT_peak_low2_prog_MT4 VT_peak_low2_prog_MT5 VT_peak_low2_prog_FT4
    VT_peak_low2_prog_FT5(1).
VARIABLE LABELS  VT_peak_low2_prog_adult 'adult-peaks low2 prog'.
EXECUTE.

RECODE VT_peak_low2_prog_MT1 VT_peak_low2_prog_MT2 VT_peak_low2_prog_MT3 VT_peak_low2_prog_MT4
    VT_peak_low2_prog_MT5 VT_peak_low2_prog_FT1 VT_peak_low2_prog_FT2 VT_peak_low2_prog_FT3
    VT_peak_low2_prog_FT4 VT_peak_low2_prog_FT5 VT_peak_low2_prog_child VT_peak_low2_prog_adult
    VT_peak_low2_prog_girl VT_peak_low2_prog_boy (SYSMIS=0).
EXECUTE.


***************scoring of the middle two pictures (middle 50%)***************
*****absolute scoring*****
*MT1*

IF  (rating11_dur_clean = MAX(rating11_dur_clean, rating26_dur_clean, rating30_dur_clean,
    rating34_dur_clean) AND rating26_dur_clean = MIN(rating11_dur_clean, rating26_dur_clean, rating30_dur_clean,
    rating34_dur_clean)) VT_abs_mid2_MT1=MEAN(rating30_dur_clean, rating34_dur_clean).
EXECUTE.

IF  (rating11_dur_clean = MAX(rating11_dur_clean, rating26_dur_clean, rating30_dur_clean,
    rating34_dur_clean) AND rating30_dur_clean = MIN(rating11_dur_clean, rating26_dur_clean, rating30_dur_clean,
    rating34_dur_clean)) VT_abs_mid2_MT1=MEAN(rating26_dur_clean, rating34_dur_clean).
EXECUTE.

IF  (rating11_dur_clean = MAX(rating11_dur_clean, rating26_dur_clean, rating30_dur_clean,
    rating34_dur_clean) AND rating34_dur_clean = MIN(rating11_dur_clean, rating26_dur_clean, rating30_dur_clean,
    rating34_dur_clean)) VT_abs_mid2_MT1=MEAN(rating26_dur_clean, rating30_dur_clean).
EXECUTE.

IF  (rating26_dur_clean = MAX(rating11_dur_clean, rating26_dur_clean, rating30_dur_clean,
    rating34_dur_clean) AND rating11_dur_clean = MIN(rating11_dur_clean, rating26_dur_clean, rating30_dur_clean,
    rating34_dur_clean)) VT_abs_mid2_MT1=MEAN(rating30_dur_clean, rating34_dur_clean).
EXECUTE.

IF  (rating26_dur_clean = MAX(rating11_dur_clean, rating26_dur_clean, rating30_dur_clean,
    rating34_dur_clean) AND rating30_dur_clean = MIN(rating11_dur_clean, rating26_dur_clean, rating30_dur_clean,
    rating34_dur_clean)) VT_abs_mid2_MT1=MEAN(rating11_dur_clean, rating34_dur_clean).
EXECUTE.

IF  (rating26_dur_clean = MAX(rating11_dur_clean, rating26_dur_clean, rating30_dur_clean,
    rating34_dur_clean) AND rating34_dur_clean = MIN(rating11_dur_clean, rating26_dur_clean, rating30_dur_clean,
    rating34_dur_clean)) VT_abs_mid2_MT1=MEAN(rating11_dur_clean, rating30_dur_clean).
EXECUTE.

IF  (rating30_dur_clean = MAX(rating11_dur_clean, rating26_dur_clean, rating30_dur_clean,
    rating34_dur_clean) AND rating11_dur_clean = MIN(rating11_dur_clean, rating26_dur_clean, rating30_dur_clean,
    rating34_dur_clean)) VT_abs_mid2_MT1=MEAN(rating26_dur_clean, rating34_dur_clean).
EXECUTE.

IF  (rating30_dur_clean = MAX(rating11_dur_clean, rating26_dur_clean, rating30_dur_clean,
    rating34_dur_clean) AND rating26_dur_clean = MIN(rating11_dur_clean, rating26_dur_clean, rating30_dur_clean,
    rating34_dur_clean)) VT_abs_mid2_MT1=MEAN(rating11_dur_clean, rating34_dur_clean).
EXECUTE.

IF  (rating30_dur_clean = MAX(rating11_dur_clean, rating26_dur_clean, rating30_dur_clean,
    rating34_dur_clean) AND rating34_dur_clean = MIN(rating11_dur_clean, rating26_dur_clean, rating30_dur_clean,
    rating34_dur_clean)) VT_abs_mid2_MT1=MEAN(rating11_dur_clean, rating26_dur_clean).
EXECUTE.

IF  (rating34_dur_clean = MAX(rating11_dur_clean, rating26_dur_clean, rating30_dur_clean,
    rating34_dur_clean) AND rating11_dur_clean = MIN(rating11_dur_clean, rating26_dur_clean, rating30_dur_clean,
    rating34_dur_clean)) VT_abs_mid2_MT1=MEAN(rating26_dur_clean, rating30_dur_clean).
EXECUTE.

IF  (rating34_dur_clean = MAX(rating11_dur_clean, rating26_dur_clean, rating30_dur_clean,
    rating34_dur_clean) AND rating26_dur_clean = MIN(rating11_dur_clean, rating26_dur_clean, rating30_dur_clean,
    rating34_dur_clean)) VT_abs_mid2_MT1=MEAN(rating11_dur_clean, rating30_dur_clean).
EXECUTE.

IF  (rating34_dur_clean = MAX(rating11_dur_clean, rating26_dur_clean, rating30_dur_clean,
    rating34_dur_clean) AND rating30_dur_clean = MIN(rating11_dur_clean, rating26_dur_clean, rating30_dur_clean,
    rating34_dur_clean)) VT_abs_mid2_MT1=MEAN(rating11_dur_clean, rating26_dur_clean).
EXECUTE.

*MT2*

IF  (rating3_dur_clean = MAX(rating3_dur_clean, rating16_dur_clean, rating32_dur_clean,
    rating38_dur_clean) AND rating16_dur_clean = MIN (rating3_dur_clean, rating16_dur_clean, rating32_dur_clean,
    rating38_dur_clean)) VT_abs_mid2_MT2=MEAN(rating32_dur_clean, rating38_dur_clean).
EXECUTE.

IF  (rating3_dur_clean = MAX(rating3_dur_clean, rating16_dur_clean, rating32_dur_clean,
    rating38_dur_clean) AND rating32_dur_clean = MIN (rating3_dur_clean, rating16_dur_clean, rating32_dur_clean,
    rating38_dur_clean)) VT_abs_mid2_MT2=MEAN(rating16_dur_clean, rating38_dur_clean).
EXECUTE.

IF  (rating3_dur_clean = MAX(rating3_dur_clean, rating16_dur_clean, rating32_dur_clean,
    rating38_dur_clean) AND rating38_dur_clean = MIN (rating3_dur_clean, rating16_dur_clean, rating32_dur_clean,
    rating38_dur_clean)) VT_abs_mid2_MT2=MEAN(rating16_dur_clean, rating32_dur_clean).
EXECUTE.

IF  (rating16_dur_clean = MAX(rating3_dur_clean, rating16_dur_clean, rating32_dur_clean,
    rating38_dur_clean) AND rating3_dur_clean = MIN (rating3_dur_clean, rating16_dur_clean, rating32_dur_clean,
    rating38_dur_clean)) VT_abs_mid2_MT2=MEAN(rating32_dur_clean, rating38_dur_clean).
EXECUTE.

IF  (rating16_dur_clean = MAX(rating3_dur_clean, rating16_dur_clean, rating32_dur_clean,
    rating38_dur_clean) AND rating32_dur_clean = MIN (rating3_dur_clean, rating16_dur_clean, rating32_dur_clean,
    rating38_dur_clean)) VT_abs_mid2_MT2=MEAN(rating3_dur_clean, rating38_dur_clean).
EXECUTE.

IF  (rating16_dur_clean = MAX(rating3_dur_clean, rating16_dur_clean, rating32_dur_clean,
    rating38_dur_clean) AND rating38_dur_clean = MIN (rating3_dur_clean, rating16_dur_clean, rating32_dur_clean,
    rating38_dur_clean)) VT_abs_mid2_MT2=MEAN(rating3_dur_clean, rating32_dur_clean).
EXECUTE.

IF  (rating32_dur_clean = MAX(rating3_dur_clean, rating16_dur_clean, rating32_dur_clean,
    rating38_dur_clean) AND rating3_dur_clean = MIN (rating3_dur_clean, rating16_dur_clean, rating32_dur_clean,
    rating38_dur_clean)) VT_abs_mid2_MT2=MEAN(rating16_dur_clean, rating38_dur_clean).
EXECUTE.

IF  (rating32_dur_clean = MAX(rating3_dur_clean, rating16_dur_clean, rating32_dur_clean,
    rating38_dur_clean) AND rating16_dur_clean = MIN (rating3_dur_clean, rating16_dur_clean, rating32_dur_clean,
    rating38_dur_clean)) VT_abs_mid2_MT2=MEAN(rating3_dur_clean, rating38_dur_clean).
EXECUTE.

IF  (rating32_dur_clean = MAX(rating3_dur_clean, rating16_dur_clean, rating32_dur_clean,
    rating38_dur_clean) AND rating38_dur_clean = MIN (rating3_dur_clean, rating16_dur_clean, rating32_dur_clean,
    rating38_dur_clean)) VT_abs_mid2_MT2=MEAN(rating3_dur_clean, rating16_dur_clean).
EXECUTE.

IF  (rating38_dur_clean = MAX(rating3_dur_clean, rating16_dur_clean, rating32_dur_clean,
    rating38_dur_clean) AND rating3_dur_clean = MIN (rating3_dur_clean, rating16_dur_clean, rating32_dur_clean,
    rating38_dur_clean)) VT_abs_mid2_MT2=MEAN(rating16_dur_clean, rating32_dur_clean).
EXECUTE.

IF  (rating38_dur_clean = MAX(rating3_dur_clean, rating16_dur_clean, rating32_dur_clean,
    rating38_dur_clean) AND rating16_dur_clean = MIN (rating3_dur_clean, rating16_dur_clean, rating32_dur_clean,
    rating38_dur_clean)) VT_abs_mid2_MT2=MEAN(rating3_dur_clean, rating32_dur_clean).
EXECUTE.

IF  (rating38_dur_clean = MAX(rating3_dur_clean, rating16_dur_clean, rating32_dur_clean,
    rating38_dur_clean) AND rating32_dur_clean = MIN (rating3_dur_clean, rating16_dur_clean, rating32_dur_clean,
    rating38_dur_clean)) VT_abs_mid2_MT2=MEAN(rating3_dur_clean, rating16_dur_clean).
EXECUTE.

*MT3*

IF  (rating6_dur_clean = MAX(rating6_dur_clean, rating13_dur_clean, rating23_dur_clean,
    rating35_dur_clean) AND rating13_dur_clean = MIN(rating6_dur_clean, rating13_dur_clean, rating23_dur_clean,
    rating35_dur_clean)) VT_abs_mid2_MT3=MEAN(rating23_dur_clean, rating35_dur_clean).
EXECUTE.

IF  (rating6_dur_clean = MAX(rating6_dur_clean, rating13_dur_clean, rating23_dur_clean,
    rating35_dur_clean) AND rating23_dur_clean = MIN(rating6_dur_clean, rating13_dur_clean, rating23_dur_clean,
    rating35_dur_clean)) VT_abs_mid2_MT3=MEAN(rating13_dur_clean, rating35_dur_clean).
EXECUTE.

IF  (rating6_dur_clean = MAX(rating6_dur_clean, rating13_dur_clean, rating23_dur_clean,
    rating35_dur_clean) AND rating35_dur_clean = MIN(rating6_dur_clean, rating13_dur_clean, rating23_dur_clean,
    rating35_dur_clean)) VT_abs_mid2_MT3=MEAN(rating13_dur_clean, rating23_dur_clean).
EXECUTE.

IF  (rating13_dur_clean = MAX(rating6_dur_clean, rating13_dur_clean, rating23_dur_clean,
    rating35_dur_clean) AND rating6_dur_clean = MIN(rating6_dur_clean, rating13_dur_clean, rating23_dur_clean,
    rating35_dur_clean)) VT_abs_mid2_MT3=MEAN(rating23_dur_clean, rating35_dur_clean).
EXECUTE.

IF  (rating13_dur_clean = MAX(rating6_dur_clean, rating13_dur_clean, rating23_dur_clean,
    rating35_dur_clean) AND rating23_dur_clean = MIN(rating6_dur_clean, rating13_dur_clean, rating23_dur_clean,
    rating35_dur_clean)) VT_abs_mid2_MT3=MEAN(rating6_dur_clean, rating35_dur_clean).
EXECUTE.

IF  (rating13_dur_clean = MAX(rating6_dur_clean, rating13_dur_clean, rating23_dur_clean,
    rating35_dur_clean) AND rating35_dur_clean = MIN(rating6_dur_clean, rating13_dur_clean, rating23_dur_clean,
    rating35_dur_clean)) VT_abs_mid2_MT3=MEAN(rating6_dur_clean, rating23_dur_clean).
EXECUTE.

IF  (rating23_dur_clean = MAX(rating6_dur_clean, rating13_dur_clean, rating23_dur_clean,
    rating35_dur_clean) AND rating6_dur_clean = MIN(rating6_dur_clean, rating13_dur_clean, rating23_dur_clean,
    rating35_dur_clean)) VT_abs_mid2_MT3=MEAN(rating13_dur_clean, rating35_dur_clean).
EXECUTE.

IF  (rating23_dur_clean = MAX(rating6_dur_clean, rating13_dur_clean, rating23_dur_clean,
    rating35_dur_clean) AND rating13_dur_clean = MIN(rating6_dur_clean, rating13_dur_clean, rating23_dur_clean,
    rating35_dur_clean)) VT_abs_mid2_MT3=MEAN(rating6_dur_clean, rating35_dur_clean).
EXECUTE.

IF  (rating23_dur_clean = MAX(rating6_dur_clean, rating13_dur_clean, rating23_dur_clean,
    rating35_dur_clean) AND rating35_dur_clean = MIN(rating6_dur_clean, rating13_dur_clean, rating23_dur_clean,
    rating35_dur_clean)) VT_abs_mid2_MT3=MEAN(rating6_dur_clean, rating13_dur_clean).
EXECUTE.

IF  (rating35_dur_clean = MAX(rating6_dur_clean, rating13_dur_clean, rating23_dur_clean,
    rating35_dur_clean) AND rating6_dur_clean = MIN(rating6_dur_clean, rating13_dur_clean, rating23_dur_clean,
    rating35_dur_clean)) VT_abs_mid2_MT3=MEAN(rating13_dur_clean, rating23_dur_clean).
EXECUTE.

IF  (rating35_dur_clean = MAX(rating6_dur_clean, rating13_dur_clean, rating23_dur_clean,
    rating35_dur_clean) AND rating13_dur_clean = MIN(rating6_dur_clean, rating13_dur_clean, rating23_dur_clean,
    rating35_dur_clean)) VT_abs_mid2_MT3=MEAN(rating6_dur_clean, rating23_dur_clean).
EXECUTE.

IF  (rating35_dur_clean = MAX(rating6_dur_clean, rating13_dur_clean, rating23_dur_clean,
    rating35_dur_clean) AND rating23_dur_clean = MIN(rating6_dur_clean, rating13_dur_clean, rating23_dur_clean,
    rating35_dur_clean)) VT_abs_mid2_MT3=MEAN(rating6_dur_clean, rating13_dur_clean).
EXECUTE.


*MT4*

IF  (rating10_dur_clean = MAX(rating10_dur_clean, rating20_dur_clean, rating25_dur_clean,
    rating36_dur_clean) AND rating20_dur_clean = MIN(rating10_dur_clean, rating20_dur_clean, rating25_dur_clean,
    rating36_dur_clean)) VT_abs_mid2_MT4=MEAN(rating25_dur_clean, rating36_dur_clean).
EXECUTE.

IF  (rating10_dur_clean = MAX(rating10_dur_clean, rating20_dur_clean, rating25_dur_clean,
    rating36_dur_clean) AND rating25_dur_clean = MIN(rating10_dur_clean, rating20_dur_clean, rating25_dur_clean,
    rating36_dur_clean)) VT_abs_mid2_MT4=MEAN(rating20_dur_clean, rating36_dur_clean).
EXECUTE.

IF  (rating10_dur_clean = MAX(rating10_dur_clean, rating20_dur_clean, rating25_dur_clean,
    rating36_dur_clean) AND rating36_dur_clean = MIN(rating10_dur_clean, rating20_dur_clean, rating25_dur_clean,
    rating36_dur_clean)) VT_abs_mid2_MT4=MEAN(rating20_dur_clean, rating25_dur_clean).
EXECUTE.

IF  (rating20_dur_clean = MAX(rating10_dur_clean, rating20_dur_clean, rating25_dur_clean,
    rating36_dur_clean) AND rating10_dur_clean = MIN(rating10_dur_clean, rating20_dur_clean, rating25_dur_clean,
    rating36_dur_clean)) VT_abs_mid2_MT4=MEAN(rating25_dur_clean, rating36_dur_clean).
EXECUTE.

IF  (rating20_dur_clean = MAX(rating10_dur_clean, rating20_dur_clean, rating25_dur_clean,
    rating36_dur_clean) AND rating25_dur_clean = MIN(rating10_dur_clean, rating20_dur_clean, rating25_dur_clean,
    rating36_dur_clean)) VT_abs_mid2_MT4=MEAN(rating10_dur_clean, rating36_dur_clean).
EXECUTE.

IF  (rating20_dur_clean = MAX(rating10_dur_clean, rating20_dur_clean, rating25_dur_clean,
    rating36_dur_clean) AND rating36_dur_clean = MIN(rating10_dur_clean, rating20_dur_clean, rating25_dur_clean,
    rating36_dur_clean)) VT_abs_mid2_MT4=MEAN(rating10_dur_clean, rating25_dur_clean).
EXECUTE.

IF  (rating25_dur_clean = MAX(rating10_dur_clean, rating20_dur_clean, rating25_dur_clean,
    rating36_dur_clean) AND rating10_dur_clean = MIN(rating10_dur_clean, rating20_dur_clean, rating25_dur_clean,
    rating36_dur_clean)) VT_abs_mid2_MT4=MEAN(rating20_dur_clean, rating36_dur_clean).
EXECUTE.

IF  (rating25_dur_clean = MAX(rating10_dur_clean, rating20_dur_clean, rating25_dur_clean,
    rating36_dur_clean) AND rating20_dur_clean = MIN(rating10_dur_clean, rating20_dur_clean, rating25_dur_clean,
    rating36_dur_clean)) VT_abs_mid2_MT4=MEAN(rating10_dur_clean, rating36_dur_clean).
EXECUTE.

IF  (rating25_dur_clean = MAX(rating10_dur_clean, rating20_dur_clean, rating25_dur_clean,
    rating36_dur_clean) AND rating36_dur_clean = MIN(rating10_dur_clean, rating20_dur_clean, rating25_dur_clean,
    rating36_dur_clean)) VT_abs_mid2_MT4=MEAN(rating10_dur_clean, rating20_dur_clean).
EXECUTE.

IF  (rating36_dur_clean = MAX(rating10_dur_clean, rating20_dur_clean, rating25_dur_clean,
    rating36_dur_clean) AND rating10_dur_clean = MIN(rating10_dur_clean, rating20_dur_clean, rating25_dur_clean,
    rating36_dur_clean)) VT_abs_mid2_MT4=MEAN(rating20_dur_clean, rating25_dur_clean).
EXECUTE.

IF  (rating36_dur_clean = MAX(rating10_dur_clean, rating20_dur_clean, rating25_dur_clean,
    rating36_dur_clean) AND rating20_dur_clean = MIN(rating10_dur_clean, rating20_dur_clean, rating25_dur_clean,
    rating36_dur_clean)) VT_abs_mid2_MT4=MEAN(rating10_dur_clean, rating25_dur_clean).
EXECUTE.

IF  (rating36_dur_clean = MAX(rating10_dur_clean, rating20_dur_clean, rating25_dur_clean,
    rating36_dur_clean) AND rating25_dur_clean = MIN(rating10_dur_clean, rating20_dur_clean, rating25_dur_clean,
    rating36_dur_clean)) VT_abs_mid2_MT4=MEAN(rating10_dur_clean, rating20_dur_clean).
EXECUTE.

*MT5*

IF  (rating8_dur_clean = MAX(rating8_dur_clean, rating12_dur_clean, rating27_dur_clean,
    rating29_dur_clean) AND rating12_dur_clean = MIN(rating8_dur_clean, rating12_dur_clean, rating27_dur_clean,
    rating29_dur_clean)) VT_abs_mid2_MT5=MEAN(rating27_dur_clean, rating29_dur_clean).
EXECUTE.

IF  (rating8_dur_clean = MAX(rating8_dur_clean, rating12_dur_clean, rating27_dur_clean,
    rating29_dur_clean) AND rating27_dur_clean = MIN(rating8_dur_clean, rating12_dur_clean, rating27_dur_clean,
    rating29_dur_clean)) VT_abs_mid2_MT5=MEAN(rating12_dur_clean, rating29_dur_clean).
EXECUTE.

IF  (rating8_dur_clean = MAX(rating8_dur_clean, rating12_dur_clean, rating27_dur_clean,
    rating29_dur_clean) AND rating29_dur_clean = MIN(rating8_dur_clean, rating12_dur_clean, rating27_dur_clean,
    rating29_dur_clean)) VT_abs_mid2_MT5=MEAN(rating12_dur_clean, rating27_dur_clean).
EXECUTE.

IF  (rating12_dur_clean = MAX(rating8_dur_clean, rating12_dur_clean, rating27_dur_clean,
    rating29_dur_clean) AND rating8_dur_clean = MIN(rating8_dur_clean, rating12_dur_clean, rating27_dur_clean,
    rating29_dur_clean)) VT_abs_mid2_MT5=MEAN(rating27_dur_clean, rating29_dur_clean).
EXECUTE.

IF  (rating12_dur_clean = MAX(rating8_dur_clean, rating12_dur_clean, rating27_dur_clean,
    rating29_dur_clean) AND rating27_dur_clean = MIN(rating8_dur_clean, rating12_dur_clean, rating27_dur_clean,
    rating29_dur_clean)) VT_abs_mid2_MT5=MEAN(rating8_dur_clean, rating29_dur_clean).
EXECUTE.

IF  (rating12_dur_clean = MAX(rating8_dur_clean, rating12_dur_clean, rating27_dur_clean,
    rating29_dur_clean) AND rating29_dur_clean = MIN(rating8_dur_clean, rating12_dur_clean, rating27_dur_clean,
    rating29_dur_clean)) VT_abs_mid2_MT5=MEAN(rating8_dur_clean, rating27_dur_clean).
EXECUTE.

IF  (rating27_dur_clean = MAX(rating8_dur_clean, rating12_dur_clean, rating27_dur_clean,
    rating29_dur_clean) AND rating8_dur_clean = MIN(rating8_dur_clean, rating12_dur_clean, rating27_dur_clean,
    rating29_dur_clean)) VT_abs_mid2_MT5=MEAN(rating12_dur_clean, rating29_dur_clean).
EXECUTE.

IF  (rating27_dur_clean = MAX(rating8_dur_clean, rating12_dur_clean, rating27_dur_clean,
    rating29_dur_clean) AND rating12_dur_clean = MIN(rating8_dur_clean, rating12_dur_clean, rating27_dur_clean,
    rating29_dur_clean)) VT_abs_mid2_MT5=MEAN(rating8_dur_clean, rating29_dur_clean).
EXECUTE.

IF  (rating27_dur_clean = MAX(rating8_dur_clean, rating12_dur_clean, rating27_dur_clean,
    rating29_dur_clean) AND rating29_dur_clean = MIN(rating8_dur_clean, rating12_dur_clean, rating27_dur_clean,
    rating29_dur_clean)) VT_abs_mid2_MT5=MEAN(rating8_dur_clean, rating12_dur_clean).
EXECUTE.

IF  (rating29_dur_clean = MAX(rating8_dur_clean, rating12_dur_clean, rating27_dur_clean,
    rating29_dur_clean) AND rating8_dur_clean = MIN(rating8_dur_clean, rating12_dur_clean, rating27_dur_clean,
    rating29_dur_clean)) VT_abs_mid2_MT5=MEAN(rating12_dur_clean, rating27_dur_clean).
EXECUTE.

IF  (rating29_dur_clean = MAX(rating8_dur_clean, rating12_dur_clean, rating27_dur_clean,
    rating29_dur_clean) AND rating12_dur_clean = MIN(rating8_dur_clean, rating12_dur_clean, rating27_dur_clean,
    rating29_dur_clean)) VT_abs_mid2_MT5=MEAN(rating8_dur_clean, rating27_dur_clean).
EXECUTE.

IF  (rating29_dur_clean = MAX(rating8_dur_clean, rating12_dur_clean, rating27_dur_clean,
    rating29_dur_clean) AND rating27_dur_clean = MIN(rating8_dur_clean, rating12_dur_clean, rating27_dur_clean,
    rating29_dur_clean)) VT_abs_mid2_MT5=MEAN(rating8_dur_clean, rating12_dur_clean).
EXECUTE.

*FT1*

IF  (rating2_dur_clean = MAX(rating2_dur_clean, rating4_dur_clean, rating15_dur_clean,
    rating31_dur_clean) AND rating4_dur_clean = MIN(rating2_dur_clean, rating4_dur_clean, rating15_dur_clean,
    rating31_dur_clean)) VT_abs_mid2_FT1=MEAN(rating15_dur_clean, rating31_dur_clean).
EXECUTE.

IF  (rating2_dur_clean = MAX(rating2_dur_clean, rating4_dur_clean, rating15_dur_clean,
    rating31_dur_clean) AND rating15_dur_clean = MIN(rating2_dur_clean, rating4_dur_clean, rating15_dur_clean,
    rating31_dur_clean)) VT_abs_mid2_FT1=MEAN(rating4_dur_clean, rating31_dur_clean).
EXECUTE.

IF  (rating2_dur_clean = MAX(rating2_dur_clean, rating4_dur_clean, rating15_dur_clean,
    rating31_dur_clean) AND rating31_dur_clean = MIN(rating2_dur_clean, rating4_dur_clean, rating15_dur_clean,
    rating31_dur_clean)) VT_abs_mid2_FT1=MEAN(rating4_dur_clean, rating15_dur_clean).
EXECUTE.

IF  (rating4_dur_clean = MAX(rating2_dur_clean, rating4_dur_clean, rating15_dur_clean,
    rating31_dur_clean) AND rating2_dur_clean = MIN(rating2_dur_clean, rating4_dur_clean, rating15_dur_clean,
    rating31_dur_clean)) VT_abs_mid2_FT1=MEAN(rating15_dur_clean, rating31_dur_clean).
EXECUTE.

IF  (rating4_dur_clean = MAX(rating2_dur_clean, rating4_dur_clean, rating15_dur_clean,
    rating31_dur_clean) AND rating15_dur_clean = MIN(rating2_dur_clean, rating4_dur_clean, rating15_dur_clean,
    rating31_dur_clean)) VT_abs_mid2_FT1=MEAN(rating2_dur_clean, rating31_dur_clean).
EXECUTE.

IF  (rating4_dur_clean = MAX(rating2_dur_clean, rating4_dur_clean, rating15_dur_clean,
    rating31_dur_clean) AND rating31_dur_clean = MIN(rating2_dur_clean, rating4_dur_clean, rating15_dur_clean,
    rating31_dur_clean)) VT_abs_mid2_FT1=MEAN(rating2_dur_clean, rating15_dur_clean).
EXECUTE.

IF  (rating15_dur_clean = MAX(rating2_dur_clean, rating4_dur_clean, rating15_dur_clean,
    rating31_dur_clean) AND rating2_dur_clean = MIN(rating2_dur_clean, rating4_dur_clean, rating15_dur_clean,
    rating31_dur_clean)) VT_abs_mid2_FT1=MEAN(rating4_dur_clean, rating31_dur_clean).
EXECUTE.

IF  (rating15_dur_clean = MAX(rating2_dur_clean, rating4_dur_clean, rating15_dur_clean,
    rating31_dur_clean) AND rating4_dur_clean = MIN(rating2_dur_clean, rating4_dur_clean, rating15_dur_clean,
    rating31_dur_clean)) VT_abs_mid2_FT1=MEAN(rating2_dur_clean, rating31_dur_clean).
EXECUTE.

IF  (rating15_dur_clean = MAX(rating2_dur_clean, rating4_dur_clean, rating15_dur_clean,
    rating31_dur_clean) AND rating31_dur_clean = MIN(rating2_dur_clean, rating4_dur_clean, rating15_dur_clean,
    rating31_dur_clean)) VT_abs_mid2_FT1=MEAN(rating2_dur_clean, rating4_dur_clean).
EXECUTE.

IF  (rating31_dur_clean = MAX(rating2_dur_clean, rating4_dur_clean, rating15_dur_clean,
    rating31_dur_clean) AND rating2_dur_clean = MIN(rating2_dur_clean, rating4_dur_clean, rating15_dur_clean,
    rating31_dur_clean)) VT_abs_mid2_FT1=MEAN(rating4_dur_clean, rating15_dur_clean).
EXECUTE.

IF  (rating31_dur_clean = MAX(rating2_dur_clean, rating4_dur_clean, rating15_dur_clean,
    rating31_dur_clean) AND rating4_dur_clean = MIN(rating2_dur_clean, rating4_dur_clean, rating15_dur_clean,
    rating31_dur_clean)) VT_abs_mid2_FT1=MEAN(rating2_dur_clean, rating15_dur_clean).
EXECUTE.

IF  (rating31_dur_clean = MAX(rating2_dur_clean, rating4_dur_clean, rating15_dur_clean,
    rating31_dur_clean) AND rating15_dur_clean = MIN(rating2_dur_clean, rating4_dur_clean, rating15_dur_clean,
    rating31_dur_clean)) VT_abs_mid2_FT1=MEAN(rating2_dur_clean, rating4_dur_clean).
EXECUTE.

*FT2*

IF  (rating1_dur_clean = MAX(rating1_dur_clean, rating9_dur_clean, rating18_dur_clean,
    rating24_dur_clean) AND rating9_dur_clean = MIN(rating1_dur_clean, rating9_dur_clean, rating18_dur_clean,
    rating24_dur_clean)) VT_abs_mid2_FT2=MEAN(rating18_dur_clean, rating24_dur_clean).
EXECUTE.

IF  (rating1_dur_clean = MAX(rating1_dur_clean, rating9_dur_clean, rating18_dur_clean,
    rating24_dur_clean) AND rating18_dur_clean = MIN(rating1_dur_clean, rating9_dur_clean, rating18_dur_clean,
    rating24_dur_clean)) VT_abs_mid2_FT2=MEAN(rating9_dur_clean, rating24_dur_clean).
EXECUTE.

IF  (rating1_dur_clean = MAX(rating1_dur_clean, rating9_dur_clean, rating18_dur_clean,
    rating24_dur_clean) AND rating24_dur_clean = MIN(rating1_dur_clean, rating9_dur_clean, rating18_dur_clean,
    rating24_dur_clean)) VT_abs_mid2_FT2=MEAN(rating9_dur_clean, rating18_dur_clean).
EXECUTE.

IF  (rating9_dur_clean = MAX(rating1_dur_clean, rating9_dur_clean, rating18_dur_clean,
    rating24_dur_clean) AND rating1_dur_clean = MIN(rating1_dur_clean, rating9_dur_clean, rating18_dur_clean,
    rating24_dur_clean)) VT_abs_mid2_FT2=MEAN(rating18_dur_clean, rating24_dur_clean).
EXECUTE.

IF  (rating9_dur_clean = MAX(rating1_dur_clean, rating9_dur_clean, rating18_dur_clean,
    rating24_dur_clean) AND rating18_dur_clean = MIN(rating1_dur_clean, rating9_dur_clean, rating18_dur_clean,
    rating24_dur_clean)) VT_abs_mid2_FT2=MEAN(rating1_dur_clean, rating24_dur_clean).
EXECUTE.

IF  (rating9_dur_clean = MAX(rating1_dur_clean, rating9_dur_clean, rating18_dur_clean,
    rating24_dur_clean) AND rating24_dur_clean = MIN(rating1_dur_clean, rating9_dur_clean, rating18_dur_clean,
    rating24_dur_clean)) VT_abs_mid2_FT2=MEAN(rating1_dur_clean, rating18_dur_clean).
EXECUTE.

IF  (rating18_dur_clean = MAX(rating1_dur_clean, rating9_dur_clean, rating18_dur_clean,
    rating24_dur_clean) AND rating1_dur_clean = MIN(rating1_dur_clean, rating9_dur_clean, rating18_dur_clean,
    rating24_dur_clean)) VT_abs_mid2_FT2=MEAN(rating9_dur_clean, rating24_dur_clean).
EXECUTE.

IF  (rating18_dur_clean = MAX(rating1_dur_clean, rating9_dur_clean, rating18_dur_clean,
    rating24_dur_clean) AND rating9_dur_clean = MIN(rating1_dur_clean, rating9_dur_clean, rating18_dur_clean,
    rating24_dur_clean)) VT_abs_mid2_FT2=MEAN(rating1_dur_clean, rating24_dur_clean).
EXECUTE.

IF  (rating18_dur_clean = MAX(rating1_dur_clean, rating9_dur_clean, rating18_dur_clean,
    rating24_dur_clean) AND rating24_dur_clean = MIN(rating1_dur_clean, rating9_dur_clean, rating18_dur_clean,
    rating24_dur_clean)) VT_abs_mid2_FT2=MEAN(rating1_dur_clean, rating9_dur_clean).
EXECUTE.

IF  (rating24_dur_clean = MAX(rating1_dur_clean, rating9_dur_clean, rating18_dur_clean,
    rating24_dur_clean) AND rating1_dur_clean = MIN(rating1_dur_clean, rating9_dur_clean, rating18_dur_clean,
    rating24_dur_clean)) VT_abs_mid2_FT2=MEAN(rating9_dur_clean, rating18_dur_clean).
EXECUTE.

IF  (rating24_dur_clean = MAX(rating1_dur_clean, rating9_dur_clean, rating18_dur_clean,
    rating24_dur_clean) AND rating9_dur_clean = MIN(rating1_dur_clean, rating9_dur_clean, rating18_dur_clean,
    rating24_dur_clean)) VT_abs_mid2_FT2=MEAN(rating1_dur_clean, rating18_dur_clean).
EXECUTE.

IF  (rating24_dur_clean = MAX(rating1_dur_clean, rating9_dur_clean, rating18_dur_clean,
    rating24_dur_clean) AND rating18_dur_clean = MIN(rating1_dur_clean, rating9_dur_clean, rating18_dur_clean,
    rating24_dur_clean)) VT_abs_mid2_FT2=MEAN(rating1_dur_clean, rating9_dur_clean).
EXECUTE.

*FT3*

IF  (rating0_dur_clean = MAX(rating0_dur_clean, rating5_dur_clean, rating14_dur_clean,
    rating17_dur_clean) AND rating5_dur_clean = MIN(rating0_dur_clean, rating5_dur_clean, rating14_dur_clean,
    rating17_dur_clean)) VT_abs_mid2_FT3=MEAN(rating14_dur_clean, rating17_dur_clean).
EXECUTE.

IF  (rating0_dur_clean = MAX(rating0_dur_clean, rating5_dur_clean, rating14_dur_clean,
    rating17_dur_clean) AND rating14_dur_clean = MIN(rating0_dur_clean, rating5_dur_clean, rating14_dur_clean,
    rating17_dur_clean)) VT_abs_mid2_FT3=MEAN(rating5_dur_clean, rating17_dur_clean).
EXECUTE.

IF  (rating0_dur_clean = MAX(rating0_dur_clean, rating5_dur_clean, rating14_dur_clean,
    rating17_dur_clean) AND rating17_dur_clean = MIN(rating0_dur_clean, rating5_dur_clean, rating14_dur_clean,
    rating17_dur_clean)) VT_abs_mid2_FT3=MEAN(rating5_dur_clean, rating14_dur_clean).
EXECUTE.

IF  (rating5_dur_clean = MAX(rating0_dur_clean, rating5_dur_clean, rating14_dur_clean,
    rating17_dur_clean) AND rating0_dur_clean = MIN(rating0_dur_clean, rating5_dur_clean, rating14_dur_clean,
    rating17_dur_clean)) VT_abs_mid2_FT3=MEAN(rating14_dur_clean, rating17_dur_clean).
EXECUTE.

IF  (rating5_dur_clean = MAX(rating0_dur_clean, rating5_dur_clean, rating14_dur_clean,
    rating17_dur_clean) AND rating14_dur_clean = MIN(rating0_dur_clean, rating5_dur_clean, rating14_dur_clean,
    rating17_dur_clean)) VT_abs_mid2_FT3=MEAN(rating0_dur_clean, rating17_dur_clean).
EXECUTE.

IF  (rating5_dur_clean = MAX(rating0_dur_clean, rating5_dur_clean, rating14_dur_clean,
    rating17_dur_clean) AND rating17_dur_clean = MIN(rating0_dur_clean, rating5_dur_clean, rating14_dur_clean,
    rating17_dur_clean)) VT_abs_mid2_FT3=MEAN(rating0_dur_clean, rating14_dur_clean).
EXECUTE.

IF  (rating14_dur_clean = MAX(rating0_dur_clean, rating5_dur_clean, rating14_dur_clean,
    rating17_dur_clean) AND rating0_dur_clean = MIN(rating0_dur_clean, rating5_dur_clean, rating14_dur_clean,
    rating17_dur_clean)) VT_abs_mid2_FT3=MEAN(rating5_dur_clean, rating17_dur_clean).
EXECUTE.

IF  (rating14_dur_clean = MAX(rating0_dur_clean, rating5_dur_clean, rating14_dur_clean,
    rating17_dur_clean) AND rating5_dur_clean = MIN(rating0_dur_clean, rating5_dur_clean, rating14_dur_clean,
    rating17_dur_clean)) VT_abs_mid2_FT3=MEAN(rating0_dur_clean, rating17_dur_clean).
EXECUTE.

IF  (rating14_dur_clean = MAX(rating0_dur_clean, rating5_dur_clean, rating14_dur_clean,
    rating17_dur_clean) AND rating17_dur_clean = MIN(rating0_dur_clean, rating5_dur_clean, rating14_dur_clean,
    rating17_dur_clean)) VT_abs_mid2_FT3=MEAN(rating0_dur_clean, rating5_dur_clean).
EXECUTE.

IF  (rating17_dur_clean = MAX(rating0_dur_clean, rating5_dur_clean, rating14_dur_clean,
    rating17_dur_clean) AND rating0_dur_clean = MIN(rating0_dur_clean, rating5_dur_clean, rating14_dur_clean,
    rating17_dur_clean)) VT_abs_mid2_FT3=MEAN(rating5_dur_clean, rating14_dur_clean).
EXECUTE.

IF  (rating17_dur_clean = MAX(rating0_dur_clean, rating5_dur_clean, rating14_dur_clean,
    rating17_dur_clean) AND rating5_dur_clean = MIN(rating0_dur_clean, rating5_dur_clean, rating14_dur_clean,
    rating17_dur_clean)) VT_abs_mid2_FT3=MEAN(rating0_dur_clean, rating14_dur_clean).
EXECUTE.

IF  (rating17_dur_clean = MAX(rating0_dur_clean, rating5_dur_clean, rating14_dur_clean,
    rating17_dur_clean) AND rating14_dur_clean = MIN(rating0_dur_clean, rating5_dur_clean, rating14_dur_clean,
    rating17_dur_clean)) VT_abs_mid2_FT3=MEAN(rating0_dur_clean, rating5_dur_clean).
EXECUTE.

*FT4*

IF  (rating7_dur_clean = MAX(rating7_dur_clean, rating21_dur_clean, rating22_dur_clean,
    rating39_dur_clean) AND rating21_dur_clean = MIN(rating7_dur_clean, rating21_dur_clean, rating22_dur_clean,
    rating39_dur_clean)) VT_abs_mid2_FT4=MEAN(rating22_dur_clean, rating39_dur_clean).
EXECUTE.

IF  (rating7_dur_clean = MAX(rating7_dur_clean, rating21_dur_clean, rating22_dur_clean,
    rating39_dur_clean) AND rating22_dur_clean = MIN(rating7_dur_clean, rating21_dur_clean, rating22_dur_clean,
    rating39_dur_clean)) VT_abs_mid2_FT4=MEAN(rating21_dur_clean, rating39_dur_clean).
EXECUTE.

IF  (rating7_dur_clean = MAX(rating7_dur_clean, rating21_dur_clean, rating22_dur_clean,
    rating39_dur_clean) AND rating39_dur_clean = MIN(rating7_dur_clean, rating21_dur_clean, rating22_dur_clean,
    rating39_dur_clean)) VT_abs_mid2_FT4=MEAN(rating21_dur_clean, rating22_dur_clean).
EXECUTE.

IF  (rating21_dur_clean = MAX(rating7_dur_clean, rating21_dur_clean, rating22_dur_clean,
    rating39_dur_clean) AND rating7_dur_clean = MIN(rating7_dur_clean, rating21_dur_clean, rating22_dur_clean,
    rating39_dur_clean)) VT_abs_mid2_FT4=MEAN(rating22_dur_clean, rating39_dur_clean).
EXECUTE.

IF  (rating21_dur_clean = MAX(rating7_dur_clean, rating21_dur_clean, rating22_dur_clean,
    rating39_dur_clean) AND rating22_dur_clean = MIN(rating7_dur_clean, rating21_dur_clean, rating22_dur_clean,
    rating39_dur_clean)) VT_abs_mid2_FT4=MEAN(rating7_dur_clean, rating39_dur_clean).
EXECUTE.

IF  (rating21_dur_clean = MAX(rating7_dur_clean, rating21_dur_clean, rating22_dur_clean,
    rating39_dur_clean) AND rating39_dur_clean = MIN(rating7_dur_clean, rating21_dur_clean, rating22_dur_clean,
    rating39_dur_clean)) VT_abs_mid2_FT4=MEAN(rating7_dur_clean, rating22_dur_clean).
EXECUTE.

IF  (rating22_dur_clean = MAX(rating7_dur_clean, rating21_dur_clean, rating22_dur_clean,
    rating39_dur_clean) AND rating7_dur_clean = MIN(rating7_dur_clean, rating21_dur_clean, rating22_dur_clean,
    rating39_dur_clean)) VT_abs_mid2_FT4=MEAN(rating21_dur_clean, rating39_dur_clean).
EXECUTE.

IF  (rating22_dur_clean = MAX(rating7_dur_clean, rating21_dur_clean, rating22_dur_clean,
    rating39_dur_clean) AND rating21_dur_clean = MIN(rating7_dur_clean, rating21_dur_clean, rating22_dur_clean,
    rating39_dur_clean)) VT_abs_mid2_FT4=MEAN(rating7_dur_clean, rating39_dur_clean).
EXECUTE.

IF  (rating22_dur_clean = MAX(rating7_dur_clean, rating21_dur_clean, rating22_dur_clean,
    rating39_dur_clean) AND rating39_dur_clean = MIN(rating7_dur_clean, rating21_dur_clean, rating22_dur_clean,
    rating39_dur_clean)) VT_abs_mid2_FT4=MEAN(rating7_dur_clean, rating21_dur_clean).
EXECUTE.

IF  (rating39_dur_clean = MAX(rating7_dur_clean, rating21_dur_clean, rating22_dur_clean,
    rating39_dur_clean) AND rating7_dur_clean = MIN(rating7_dur_clean, rating21_dur_clean, rating22_dur_clean,
    rating39_dur_clean)) VT_abs_mid2_FT4=MEAN(rating21_dur_clean, rating22_dur_clean).
EXECUTE.

IF  (rating39_dur_clean = MAX(rating7_dur_clean, rating21_dur_clean, rating22_dur_clean,
    rating39_dur_clean) AND rating21_dur_clean = MIN(rating7_dur_clean, rating21_dur_clean, rating22_dur_clean,
    rating39_dur_clean)) VT_abs_mid2_FT4=MEAN(rating7_dur_clean, rating22_dur_clean).
EXECUTE.

IF  (rating39_dur_clean = MAX(rating7_dur_clean, rating21_dur_clean, rating22_dur_clean,
    rating39_dur_clean) AND rating22_dur_clean = MIN(rating7_dur_clean, rating21_dur_clean, rating22_dur_clean,
    rating39_dur_clean)) VT_abs_mid2_FT4=MEAN(rating7_dur_clean, rating21_dur_clean).
EXECUTE.

*FT5*

IF  (rating19_dur_clean = MAX(rating19_dur_clean, rating28_dur_clean, rating33_dur_clean,
    rating37_dur_clean) AND rating28_dur_clean = MIN(rating19_dur_clean, rating28_dur_clean, rating33_dur_clean,
    rating37_dur_clean)) VT_abs_mid2_FT5=MEAN(rating33_dur_clean, rating37_dur_clean).
EXECUTE.

IF  (rating19_dur_clean = MAX(rating19_dur_clean, rating28_dur_clean, rating33_dur_clean,
    rating37_dur_clean) AND rating33_dur_clean = MIN(rating19_dur_clean, rating28_dur_clean, rating33_dur_clean,
    rating37_dur_clean)) VT_abs_mid2_FT5=MEAN(rating28_dur_clean, rating37_dur_clean).
EXECUTE.

IF  (rating19_dur_clean = MAX(rating19_dur_clean, rating28_dur_clean, rating33_dur_clean,
    rating37_dur_clean) AND rating37_dur_clean = MIN(rating19_dur_clean, rating28_dur_clean, rating33_dur_clean,
    rating37_dur_clean)) VT_abs_mid2_FT5=MEAN(rating28_dur_clean, rating33_dur_clean).
EXECUTE.

IF  (rating28_dur_clean = MAX(rating19_dur_clean, rating28_dur_clean, rating33_dur_clean,
    rating37_dur_clean) AND rating19_dur_clean = MIN(rating19_dur_clean, rating28_dur_clean, rating33_dur_clean,
    rating37_dur_clean)) VT_abs_mid2_FT5=MEAN(rating33_dur_clean, rating37_dur_clean).
EXECUTE.

IF  (rating28_dur_clean = MAX(rating19_dur_clean, rating28_dur_clean, rating33_dur_clean,
    rating37_dur_clean) AND rating33_dur_clean = MIN(rating19_dur_clean, rating28_dur_clean, rating33_dur_clean,
    rating37_dur_clean)) VT_abs_mid2_FT5=MEAN(rating19_dur_clean, rating37_dur_clean).
EXECUTE.

IF  (rating28_dur_clean = MAX(rating19_dur_clean, rating28_dur_clean, rating33_dur_clean,
    rating37_dur_clean) AND rating37_dur_clean = MIN(rating19_dur_clean, rating28_dur_clean, rating33_dur_clean,
    rating37_dur_clean)) VT_abs_mid2_FT5=MEAN(rating19_dur_clean, rating33_dur_clean).
EXECUTE.

IF  (rating33_dur_clean = MAX(rating19_dur_clean, rating28_dur_clean, rating33_dur_clean,
    rating37_dur_clean) AND rating19_dur_clean = MIN(rating19_dur_clean, rating28_dur_clean, rating33_dur_clean,
    rating37_dur_clean)) VT_abs_mid2_FT5=MEAN(rating28_dur_clean, rating37_dur_clean).
EXECUTE.

IF  (rating33_dur_clean = MAX(rating19_dur_clean, rating28_dur_clean, rating33_dur_clean,
    rating37_dur_clean) AND rating28_dur_clean = MIN(rating19_dur_clean, rating28_dur_clean, rating33_dur_clean,
    rating37_dur_clean)) VT_abs_mid2_FT5=MEAN(rating19_dur_clean, rating37_dur_clean).
EXECUTE.

IF  (rating33_dur_clean = MAX(rating19_dur_clean, rating28_dur_clean, rating33_dur_clean,
    rating37_dur_clean) AND rating37_dur_clean = MIN(rating19_dur_clean, rating28_dur_clean, rating33_dur_clean,
    rating37_dur_clean)) VT_abs_mid2_FT5=MEAN(rating19_dur_clean, rating28_dur_clean).
EXECUTE.

IF  (rating37_dur_clean = MAX(rating19_dur_clean, rating28_dur_clean, rating33_dur_clean,
    rating37_dur_clean) AND rating19_dur_clean = MIN(rating19_dur_clean, rating28_dur_clean, rating33_dur_clean,
    rating37_dur_clean)) VT_abs_mid2_FT5=MEAN(rating28_dur_clean, rating33_dur_clean).
EXECUTE.

IF  (rating37_dur_clean = MAX(rating19_dur_clean, rating28_dur_clean, rating33_dur_clean,
    rating37_dur_clean) AND rating28_dur_clean = MIN(rating19_dur_clean, rating28_dur_clean, rating33_dur_clean,
    rating37_dur_clean)) VT_abs_mid2_FT5=MEAN(rating19_dur_clean, rating33_dur_clean).
EXECUTE.

IF  (rating37_dur_clean = MAX(rating19_dur_clean, rating28_dur_clean, rating33_dur_clean,
    rating37_dur_clean) AND rating33_dur_clean = MIN(rating19_dur_clean, rating28_dur_clean, rating33_dur_clean,
    rating37_dur_clean)) VT_abs_mid2_FT5=MEAN(rating19_dur_clean, rating28_dur_clean).
EXECUTE.


*****relative scoring*****

COMPUTE VT_rel_D_mid2=(MEAN(VT_abs_mid2_MT1, VT_abs_mid2_MT2, VT_abs_mid2_MT3, VT_abs_mid2_FT1, VT_abs_mid2_FT2,
    VT_abs_mid2_FT3)) - (MEAN(VT_abs_mid2_MT4, VT_abs_mid2_MT5, VT_abs_mid2_FT4, VT_abs_mid2_FT5)).
EXECUTE.


*****peak detection*****
***conservative (1 SD)***
*by categories*

IF  ((VT_abs_mid2_MT1 > VT_abs_mid2_MT2) AND (VT_abs_mid2_MT1 > (MEAN(VT_abs_mid2_MT2, VT_abs_mid2_MT3, VT_abs_mid2_MT4,
    VT_abs_mid2_MT5, VT_abs_mid2_FT1, VT_abs_mid2_FT2, VT_abs_mid2_FT3, VT_abs_mid2_FT4, VT_abs_mid2_FT5) + SD(VT_abs_mid2_MT2,
    VT_abs_mid2_MT3, VT_abs_mid2_MT4, VT_abs_mid2_MT5, VT_abs_mid2_FT1, VT_abs_mid2_FT2, VT_abs_mid2_FT3, VT_abs_mid2_FT4, VT_abs_mid2_FT5))))
    VT_peak_mid2_kons_MT1=1.
EXECUTE.

IF  ((VT_abs_mid2_MT2 > VT_abs_mid2_MT1) AND (VT_abs_mid2_MT2 > VT_abs_mid2_MT3) AND (VT_abs_mid2_MT2 > (MEAN(VT_abs_mid2_MT1, VT_abs_mid2_MT3, VT_abs_mid2_MT4,
    VT_abs_mid2_MT5, VT_abs_mid2_FT1, VT_abs_mid2_FT2, VT_abs_mid2_FT3, VT_abs_mid2_FT4, VT_abs_mid2_FT5) + SD(VT_abs_mid2_MT1,
    VT_abs_mid2_MT3, VT_abs_mid2_MT4, VT_abs_mid2_MT5, VT_abs_mid2_FT1, VT_abs_mid2_FT2, VT_abs_mid2_FT3, VT_abs_mid2_FT4, VT_abs_mid2_FT5))))
    VT_peak_mid2_kons_MT2=1.
EXECUTE.

IF  ((VT_abs_mid2_MT3 > VT_abs_mid2_MT2) AND (VT_abs_mid2_MT3 > VT_abs_mid2_MT4) AND (VT_abs_mid2_MT3 > (MEAN(VT_abs_mid2_MT1, VT_abs_mid2_MT2, VT_abs_mid2_MT4,
    VT_abs_mid2_MT5, VT_abs_mid2_FT1, VT_abs_mid2_FT2, VT_abs_mid2_FT3, VT_abs_mid2_FT4, VT_abs_mid2_FT5) + SD(VT_abs_mid2_MT1,
    VT_abs_mid2_MT2, VT_abs_mid2_MT4, VT_abs_mid2_MT5, VT_abs_mid2_FT1, VT_abs_mid2_FT2, VT_abs_mid2_FT3, VT_abs_mid2_FT4, VT_abs_mid2_FT5))))
    VT_peak_mid2_kons_MT3=1.
EXECUTE.

IF  ((VT_abs_mid2_MT4 > VT_abs_mid2_MT3) AND (VT_abs_mid2_MT4 > VT_abs_mid2_MT5) AND (VT_abs_mid2_MT4 > (MEAN(VT_abs_mid2_MT1, VT_abs_mid2_MT2, VT_abs_mid2_MT3,
    VT_abs_mid2_MT5, VT_abs_mid2_FT1, VT_abs_mid2_FT2, VT_abs_mid2_FT3, VT_abs_mid2_FT4, VT_abs_mid2_FT5) + SD(VT_abs_mid2_MT1,
    VT_abs_mid2_MT2, VT_abs_mid2_MT3, VT_abs_mid2_MT5, VT_abs_mid2_FT1, VT_abs_mid2_FT2, VT_abs_mid2_FT3, VT_abs_mid2_FT4, VT_abs_mid2_FT5))))
    VT_peak_mid2_kons_MT4=1.
EXECUTE.

IF  ((VT_abs_mid2_MT5 > VT_abs_mid2_MT4) AND (VT_abs_mid2_MT5 > (MEAN(VT_abs_mid2_MT1, VT_abs_mid2_MT2, VT_abs_mid2_MT3,
    VT_abs_mid2_MT4, VT_abs_mid2_FT1, VT_abs_mid2_FT2, VT_abs_mid2_FT3, VT_abs_mid2_FT4, VT_abs_mid2_FT5) + SD(VT_abs_mid2_MT1,
    VT_abs_mid2_MT2, VT_abs_mid2_MT3, VT_abs_mid2_MT4, VT_abs_mid2_FT1, VT_abs_mid2_FT2, VT_abs_mid2_FT3, VT_abs_mid2_FT4, VT_abs_mid2_FT5))))
    VT_peak_mid2_kons_MT5=1.
EXECUTE.

IF  ((VT_abs_mid2_FT1 > VT_abs_mid2_FT2) AND (VT_abs_mid2_FT1 > (MEAN(VT_abs_mid2_MT1, VT_abs_mid2_MT2, VT_abs_mid2_MT3,
    VT_abs_mid2_MT4, VT_abs_mid2_MT5, VT_abs_mid2_FT2, VT_abs_mid2_FT3, VT_abs_mid2_FT4, VT_abs_mid2_FT5) + SD(VT_abs_mid2_MT1,
    VT_abs_mid2_MT2, VT_abs_mid2_MT3, VT_abs_mid2_MT4, VT_abs_mid2_MT5, VT_abs_mid2_FT2, VT_abs_mid2_FT3, VT_abs_mid2_FT4, VT_abs_mid2_FT5))))
    VT_peak_mid2_kons_FT1=1.
EXECUTE.

IF  ((VT_abs_mid2_FT2 > VT_abs_mid2_FT1) AND (VT_abs_mid2_FT2 > VT_abs_mid2_FT3) AND (VT_abs_mid2_FT2 > (MEAN(VT_abs_mid2_MT1, VT_abs_mid2_MT2, VT_abs_mid2_MT3,
    VT_abs_mid2_MT4, VT_abs_mid2_MT5, VT_abs_mid2_FT1, VT_abs_mid2_FT3, VT_abs_mid2_FT4, VT_abs_mid2_FT5) + SD(VT_abs_mid2_MT1,
    VT_abs_mid2_MT2, VT_abs_mid2_MT3, VT_abs_mid2_MT4, VT_abs_mid2_MT5, VT_abs_mid2_FT1, VT_abs_mid2_FT3, VT_abs_mid2_FT4, VT_abs_mid2_FT5))))
    VT_peak_mid2_kons_FT2=1.
EXECUTE.

IF  ((VT_abs_mid2_FT3 > VT_abs_mid2_FT2) AND (VT_abs_mid2_FT3 > VT_abs_mid2_FT4) AND (VT_abs_mid2_FT3 > (MEAN(VT_abs_mid2_MT1, VT_abs_mid2_MT2, VT_abs_mid2_MT3,
    VT_abs_mid2_MT4, VT_abs_mid2_MT5, VT_abs_mid2_FT1, VT_abs_mid2_FT2, VT_abs_mid2_FT4, VT_abs_mid2_FT5) + SD(VT_abs_mid2_MT1,
    VT_abs_mid2_MT2, VT_abs_mid2_MT3, VT_abs_mid2_MT4, VT_abs_mid2_MT5, VT_abs_mid2_FT1, VT_abs_mid2_FT2, VT_abs_mid2_FT4, VT_abs_mid2_FT5))))
    VT_peak_mid2_kons_FT3=1.
EXECUTE.

IF  ((VT_abs_mid2_FT4 > VT_abs_mid2_FT3) AND (VT_abs_mid2_FT4 > VT_abs_mid2_FT5) AND (VT_abs_mid2_FT4 > (MEAN(VT_abs_mid2_MT1, VT_abs_mid2_MT2, VT_abs_mid2_MT3,
    VT_abs_mid2_MT4, VT_abs_mid2_MT5, VT_abs_mid2_FT1, VT_abs_mid2_FT2, VT_abs_mid2_FT3, VT_abs_mid2_FT5) + SD(VT_abs_mid2_MT1,
    VT_abs_mid2_MT2, VT_abs_mid2_MT3, VT_abs_mid2_MT4, VT_abs_mid2_MT5, VT_abs_mid2_FT1, VT_abs_mid2_FT2, VT_abs_mid2_FT3, VT_abs_mid2_FT5))))
    VT_peak_mid2_kons_FT4=1.
EXECUTE.

IF  ((VT_abs_mid2_FT5 > VT_abs_mid2_FT4) AND (VT_abs_mid2_FT5 > (MEAN(VT_abs_mid2_MT1, VT_abs_mid2_MT2, VT_abs_mid2_MT3,
    VT_abs_mid2_MT4, VT_abs_mid2_MT5, VT_abs_mid2_FT1, VT_abs_mid2_FT2, VT_abs_mid2_FT3, VT_abs_mid2_FT4) + SD(VT_abs_mid2_MT1,
    VT_abs_mid2_MT2, VT_abs_mid2_MT3, VT_abs_mid2_MT4, VT_abs_mid2_MT5, VT_abs_mid2_FT1, VT_abs_mid2_FT2, VT_abs_mid2_FT3, VT_abs_mid2_FT4))))
    VT_peak_mid2_kons_FT5=1.
EXECUTE.

RECODE VT_peak_mid2_kons_MT1 VT_peak_mid2_kons_MT2 VT_peak_mid2_kons_MT3 VT_peak_mid2_kons_MT4
    VT_peak_mid2_kons_MT5 VT_peak_mid2_kons_FT1 VT_peak_mid2_kons_FT2 VT_peak_mid2_kons_FT3
    VT_peak_mid2_kons_FT4 VT_peak_mid2_kons_FT5(SYSMIS=0).
EXECUTE.
COUNT VT_peak_mid2_kons_child=VT_peak_mid2_kons_MT1 VT_peak_mid2_kons_MT2 VT_peak_mid2_kons_MT3
    VT_peak_mid2_kons_FT1 VT_peak_mid2_kons_FT2 VT_peak_mid2_kons_FT3(1).
VARIABLE LABELS  VT_peak_mid2_kons_child 'children-peaks mid2 kons'.
EXECUTE.

COUNT VT_peak_mid2_kons_girl=VT_peak_mid2_kons_FT1 VT_peak_mid2_kons_FT2 VT_peak_mid2_kons_FT3(1).
VARIABLE LABELS  VT_peak_mid2_kons_girl 'Mädchen-peaks mid2 kons'.
EXECUTE.

COUNT VT_peak_mid2_kons_boy=VT_peak_mid2_kons_MT1 VT_peak_mid2_kons_MT2 VT_peak_mid2_kons_MT3(1).
VARIABLE LABELS  VT_peak_mid2_kons_boy 'Jungen-peaks mid2 kons'.
EXECUTE.

COUNT VT_peak_mid2_kons_adult=VT_peak_mid2_kons_MT4 VT_peak_mid2_kons_MT5 VT_peak_mid2_kons_FT4
    VT_peak_mid2_kons_FT5(1).
VARIABLE LABELS  VT_peak_mid2_kons_adult 'adult-peaks mid2 kons'.
EXECUTE.

RECODE VT_peak_mid2_kons_child VT_peak_mid2_kons_adult VT_peak_mid2_kons_girl VT_peak_mid2_kons_boy(SYSMIS=0).
EXECUTE.


***progressive (1/2 SD)***
*by categories*

IF  ((VT_abs_mid2_MT1 > VT_abs_mid2_MT2) AND (VT_abs_mid2_MT1 > (MEAN(VT_abs_mid2_MT2, VT_abs_mid2_MT3, VT_abs_mid2_MT4,
    VT_abs_mid2_MT5, VT_abs_mid2_FT1, VT_abs_mid2_FT2, VT_abs_mid2_FT3, VT_abs_mid2_FT4, VT_abs_mid2_FT5) + (0.5 * SD(VT_abs_mid2_MT2,
    VT_abs_mid2_MT3, VT_abs_mid2_MT4, VT_abs_mid2_MT5, VT_abs_mid2_FT1, VT_abs_mid2_FT2, VT_abs_mid2_FT3, VT_abs_mid2_FT4, VT_abs_mid2_FT5)))))
    VT_peak_mid2_prog_MT1=1.
EXECUTE.

IF  ((VT_abs_mid2_MT2 > VT_abs_mid2_MT1) AND (VT_abs_mid2_MT2 > VT_abs_mid2_MT3) AND (VT_abs_mid2_MT2 > (MEAN(VT_abs_mid2_MT1, VT_abs_mid2_MT3, VT_abs_mid2_MT4,
    VT_abs_mid2_MT5, VT_abs_mid2_FT1, VT_abs_mid2_FT2, VT_abs_mid2_FT3, VT_abs_mid2_FT4, VT_abs_mid2_FT5) + (0.5 * SD(VT_abs_mid2_MT1,
    VT_abs_mid2_MT3, VT_abs_mid2_MT4, VT_abs_mid2_MT5, VT_abs_mid2_FT1, VT_abs_mid2_FT2, VT_abs_mid2_FT3, VT_abs_mid2_FT4, VT_abs_mid2_FT5)))))
    VT_peak_mid2_prog_MT2=1.
EXECUTE.

IF  ((VT_abs_mid2_MT3 > VT_abs_mid2_MT2) AND (VT_abs_mid2_MT3 > VT_abs_mid2_MT4) AND (VT_abs_mid2_MT3 > (MEAN(VT_abs_mid2_MT1, VT_abs_mid2_MT2, VT_abs_mid2_MT4,
    VT_abs_mid2_MT5, VT_abs_mid2_FT1, VT_abs_mid2_FT2, VT_abs_mid2_FT3, VT_abs_mid2_FT4, VT_abs_mid2_FT5) + (0.5 * SD(VT_abs_mid2_MT1,
    VT_abs_mid2_MT2, VT_abs_mid2_MT4, VT_abs_mid2_MT5, VT_abs_mid2_FT1, VT_abs_mid2_FT2, VT_abs_mid2_FT3, VT_abs_mid2_FT4, VT_abs_mid2_FT5)))))
    VT_peak_mid2_prog_MT3=1.
EXECUTE.

IF  ((VT_abs_mid2_MT4 > VT_abs_mid2_MT3) AND (VT_abs_mid2_MT4 > VT_abs_mid2_MT5) AND (VT_abs_mid2_MT4 > (MEAN(VT_abs_mid2_MT1, VT_abs_mid2_MT2, VT_abs_mid2_MT3,
    VT_abs_mid2_MT5, VT_abs_mid2_FT1, VT_abs_mid2_FT2, VT_abs_mid2_FT3, VT_abs_mid2_FT4, VT_abs_mid2_FT5) + (0.5 * SD(VT_abs_mid2_MT1,
    VT_abs_mid2_MT2, VT_abs_mid2_MT3, VT_abs_mid2_MT5, VT_abs_mid2_FT1, VT_abs_mid2_FT2, VT_abs_mid2_FT3, VT_abs_mid2_FT4, VT_abs_mid2_FT5)))))
    VT_peak_mid2_prog_MT4=1.
EXECUTE.

IF  ((VT_abs_mid2_MT5 > VT_abs_mid2_MT4) AND (VT_abs_mid2_MT5 > (MEAN(VT_abs_mid2_MT1, VT_abs_mid2_MT2, VT_abs_mid2_MT3,
    VT_abs_mid2_MT4, VT_abs_mid2_FT1, VT_abs_mid2_FT2, VT_abs_mid2_FT3, VT_abs_mid2_FT4, VT_abs_mid2_FT5) + (0.5 * SD(VT_abs_mid2_MT1,
    VT_abs_mid2_MT2, VT_abs_mid2_MT3, VT_abs_mid2_MT4, VT_abs_mid2_FT1, VT_abs_mid2_FT2, VT_abs_mid2_FT3, VT_abs_mid2_FT4, VT_abs_mid2_FT5)))))
    VT_peak_mid2_prog_MT5=1.
EXECUTE.

IF  ((VT_abs_mid2_FT1 > VT_abs_mid2_FT2) AND (VT_abs_mid2_FT1 > (MEAN(VT_abs_mid2_MT1, VT_abs_mid2_MT2, VT_abs_mid2_MT3,
    VT_abs_mid2_MT4, VT_abs_mid2_MT5, VT_abs_mid2_FT2, VT_abs_mid2_FT3, VT_abs_mid2_FT4, VT_abs_mid2_FT5) + (0.5 * SD(VT_abs_mid2_MT1,
    VT_abs_mid2_MT2, VT_abs_mid2_MT3, VT_abs_mid2_MT4, VT_abs_mid2_MT5, VT_abs_mid2_FT2, VT_abs_mid2_FT3, VT_abs_mid2_FT4, VT_abs_mid2_FT5)))))
    VT_peak_mid2_prog_FT1=1.
EXECUTE.

IF  ((VT_abs_mid2_FT2 > VT_abs_mid2_FT1) AND (VT_abs_mid2_FT2 > VT_abs_mid2_FT3) AND (VT_abs_mid2_FT2 > (MEAN(VT_abs_mid2_MT1, VT_abs_mid2_MT2, VT_abs_mid2_MT3,
    VT_abs_mid2_MT4, VT_abs_mid2_MT5, VT_abs_mid2_FT1, VT_abs_mid2_FT3, VT_abs_mid2_FT4, VT_abs_mid2_FT5) + (0.5 * SD(VT_abs_mid2_MT1,
    VT_abs_mid2_MT2, VT_abs_mid2_MT3, VT_abs_mid2_MT4, VT_abs_mid2_MT5, VT_abs_mid2_FT1, VT_abs_mid2_FT3, VT_abs_mid2_FT4, VT_abs_mid2_FT5)))))
    VT_peak_mid2_prog_FT2=1.
EXECUTE.

IF  ((VT_abs_mid2_FT3 > VT_abs_mid2_FT2) AND (VT_abs_mid2_FT3 > VT_abs_mid2_FT4) AND (VT_abs_mid2_FT3 > (MEAN(VT_abs_mid2_MT1, VT_abs_mid2_MT2, VT_abs_mid2_MT3,
    VT_abs_mid2_MT4, VT_abs_mid2_MT5, VT_abs_mid2_FT1, VT_abs_mid2_FT2, VT_abs_mid2_FT4, VT_abs_mid2_FT5) + (0.5 * SD(VT_abs_mid2_MT1,
    VT_abs_mid2_MT2, VT_abs_mid2_MT3, VT_abs_mid2_MT4, VT_abs_mid2_MT5, VT_abs_mid2_FT1, VT_abs_mid2_FT2, VT_abs_mid2_FT4, VT_abs_mid2_FT5)))))
    VT_peak_mid2_prog_FT3=1.
EXECUTE.

IF  ((VT_abs_mid2_FT4 > VT_abs_mid2_FT3) AND (VT_abs_mid2_FT4 > VT_abs_mid2_FT5) AND (VT_abs_mid2_FT4 > (MEAN(VT_abs_mid2_MT1, VT_abs_mid2_MT2, VT_abs_mid2_MT3,
    VT_abs_mid2_MT4, VT_abs_mid2_MT5, VT_abs_mid2_FT1, VT_abs_mid2_FT2, VT_abs_mid2_FT3, VT_abs_mid2_FT5) + (0.5 * SD(VT_abs_mid2_MT1,
    VT_abs_mid2_MT2, VT_abs_mid2_MT3, VT_abs_mid2_MT4, VT_abs_mid2_MT5, VT_abs_mid2_FT1, VT_abs_mid2_FT2, VT_abs_mid2_FT3, VT_abs_mid2_FT5)))))
    VT_peak_mid2_prog_FT4=1.
EXECUTE.

IF  ((VT_abs_mid2_FT5 > VT_abs_mid2_FT4) AND (VT_abs_mid2_FT5 > (MEAN(VT_abs_mid2_MT1, VT_abs_mid2_MT2, VT_abs_mid2_MT3,
    VT_abs_mid2_MT4, VT_abs_mid2_MT5, VT_abs_mid2_FT1, VT_abs_mid2_FT2, VT_abs_mid2_FT3, VT_abs_mid2_FT4) + (0.5 * SD(VT_abs_mid2_MT1,
    VT_abs_mid2_MT2, VT_abs_mid2_MT3, VT_abs_mid2_MT4, VT_abs_mid2_MT5, VT_abs_mid2_FT1, VT_abs_mid2_FT2, VT_abs_mid2_FT3, VT_abs_mid2_FT4)))))
    VT_peak_mid2_prog_FT5=1.
EXECUTE.

RECODE VT_peak_mid2_prog_MT1 VT_peak_mid2_prog_MT2 VT_peak_mid2_prog_MT3 VT_peak_mid2_prog_MT4
    VT_peak_mid2_prog_MT5 VT_peak_mid2_prog_FT1 VT_peak_mid2_prog_FT2 VT_peak_mid2_prog_FT3
    VT_peak_mid2_prog_FT4 VT_peak_mid2_prog_FT5(SYSMIS=0).
EXECUTE.

COUNT VT_peak_mid2_prog_child=VT_peak_mid2_prog_MT1 VT_peak_mid2_prog_MT2 VT_peak_mid2_prog_MT3
    VT_peak_mid2_prog_FT1 VT_peak_mid2_prog_FT2 VT_peak_mid2_prog_FT3(1).
VARIABLE LABELS  VT_peak_mid2_prog_child 'children-peaks mid2 prog'.
EXECUTE.

COUNT VT_peak_mid2_prog_girl=VT_peak_mid2_prog_FT1 VT_peak_mid2_prog_FT2 VT_peak_mid2_prog_FT3(1).
VARIABLE LABELS  VT_peak_mid2_prog_girl 'Mädchen-peaks mid2 prog'.
EXECUTE.
COUNT VT_peak_mid2_prog_boy=VT_peak_mid2_prog_MT1 VT_peak_mid2_prog_MT2 VT_peak_mid2_prog_MT3(1).
VARIABLE LABELS  VT_peak_mid2_prog_boy 'Jungen-peaks mid2 prog'.
EXECUTE.

COUNT VT_peak_mid2_prog_adult=VT_peak_mid2_prog_MT4 VT_peak_mid2_prog_MT5 VT_peak_mid2_prog_FT4
    VT_peak_mid2_prog_FT5(1).
VARIABLE LABELS  VT_peak_mid2_prog_adult 'adult-peaks mid2 prog'.
EXECUTE.

RECODE VT_peak_mid2_prog_child VT_peak_mid2_prog_adult VT_peak_mid2_prog_girl VT_peak_mid2_prog_boy(SYSMIS=0).
EXECUTE.


***************peak-scoring with conservative values***************
*****peak score 1*****

COMPUTE VT_peaksc1_kons = 0.
EXECUTE.

IF (VT_peak_kons_girl > 0) VT_peaksc1_kons=VT_peaksc1_kons + 2.
EXECUTE.

IF (VT_peak_kons_boy > 0) VT_peaksc1_kons=VT_peaksc1_kons + 2.
EXECUTE.

IF (VT_peak_kons_adult > 0) VT_peaksc1_kons=VT_peaksc1_kons -1.
EXECUTE.

IF (VT_peak_low2_kons_girl > 0) VT_peaksc1_kons=VT_peaksc1_kons + 1.
EXECUTE.

IF (VT_peak_low2_kons_boy > 0) VT_peaksc1_kons=VT_peaksc1_kons + 1.
EXECUTE.

IF (VT_peak_low2_kons_adult > 0) VT_peaksc1_kons=VT_peaksc1_kons - 0.5.
EXECUTE.


*****peak score 2*****

COMPUTE VT_peaksc2_kons = 0.
EXECUTE.

IF (VT_peak_kons_girl > 0) VT_peaksc2_kons=VT_peaksc2_kons + 2.
EXECUTE.

IF (VT_peak_kons_boy > 0) VT_peaksc2_kons=VT_peaksc2_kons+ 3.
EXECUTE.

IF (VT_peak_kons_adult > 0) VT_peaksc2_kons=VT_peaksc2_kons -1.
EXECUTE.

IF (VT_peak_low2_kons_girl > 0) VT_peaksc2_kons=VT_peaksc2_kons + 1.
EXECUTE.

IF (VT_peak_low2_kons_boy > 0) VT_peaksc2_kons=VT_peaksc2_kons + 1.5.
EXECUTE.

IF (VT_peak_low2_kons_adult > 0) VT_peaksc2_kons=VT_peaksc2_kons - 0.5.
EXECUTE.


*****peak score 3*****

COMPUTE VT_peaksc3_kons = 0.
EXECUTE.

IF (VT_peak_kons_girl > 0) VT_peaksc3_kons=VT_peaksc3_kons + 1.
EXECUTE.

IF (VT_peak_kons_boy > 0) VT_peaksc3_kons=VT_peaksc3_kons + 1.
EXECUTE.

IF (VT_peak_kons_adult > 0) VT_peaksc3_kons=VT_peaksc3_kons -0.5.
EXECUTE.

IF (VT_peak_low3_kons_girl > 0) VT_peaksc3_kons=VT_peaksc3_kons + 1.
EXECUTE.

IF (VT_peak_low3_kons_boy > 0) VT_peaksc3_kons=VT_peaksc3_kons + 1.
EXECUTE.

IF (VT_peak_low3_kons_adult > 0) VT_peaksc3_kons=VT_peaksc3_kons - 0.5.
EXECUTE.

IF (VT_peak_low2_kons_girl > 0) VT_peaksc3_kons=VT_peaksc3_kons + 1.
EXECUTE.

IF (VT_peak_low2_kons_boy > 0) VT_peaksc3_kons=VT_peaksc3_kons + 1.
EXECUTE.

IF (VT_peak_low2_kons_adult > 0) VT_peaksc3_kons=VT_peaksc3_kons - 0.5.
EXECUTE.


*****peak score 4*****

COMPUTE VT_peaksc4_kons = 0.
EXECUTE.

IF (VT_peak_kons_girl > 0) VT_peaksc4_kons=VT_peaksc4_kons + 1.
EXECUTE.

IF (VT_peak_kons_boy > 0) VT_peaksc4_kons=VT_peaksc4_kons + 1.
EXECUTE.

IF (VT_peak_kons_adult > 0) VT_peaksc4_kons=VT_peaksc4_kons -0.5.
EXECUTE.

IF (VT_peak_low3_kons_girl > 0) VT_peaksc4_kons=VT_peaksc4_kons + 1.
EXECUTE.

IF (VT_peak_low3_kons_boy > 0) VT_peaksc4_kons=VT_peaksc4_kons + 1.
EXECUTE.

IF (VT_peak_low3_kons_adult > 0) VT_peaksc4_kons=VT_peaksc4_kons - 0.5.
EXECUTE.

IF (VT_peak_low2_kons_girl > 0) VT_peaksc4_kons=VT_peaksc4_kons + 1.
EXECUTE.

IF (VT_peak_low2_kons_boy > 0) VT_peaksc4_kons=VT_peaksc4_kons + 1.
EXECUTE.

IF (VT_peak_low2_kons_adult > 0) VT_peaksc4_kons=VT_peaksc4_kons - 0.5.
EXECUTE.

IF (VT_peak_mid2_kons_girl > 0) VT_peaksc4_kons=VT_peaksc4_kons + 1.
EXECUTE.

IF (VT_peak_mid2_kons_boy > 0) VT_peaksc4_kons=VT_peaksc4_kons + 1.
EXECUTE.

IF (VT_peak_mid2_kons_adult > 0) VT_peaksc4_kons=VT_peaksc4_kons - 0.5.
EXECUTE.


*****peak score 5*****

COMPUTE VT_peaksc5_kons = 0.
EXECUTE.

IF (VT_peak_kons_girl > 0) VT_peaksc5_kons=VT_peaksc5_kons + 1.
EXECUTE.

IF (VT_peak_kons_boy > 0) VT_peaksc5_kons=VT_peaksc5_kons + 1.
EXECUTE.

IF (VT_peak_kons_adult > 0) VT_peaksc5_kons=VT_peaksc5_kons -0.5.
EXECUTE.

IF (VT_peak_low3_kons_girl > 0) VT_peaksc5_kons=VT_peaksc5_kons + 2.
EXECUTE.

IF (VT_peak_low3_kons_boy > 0) VT_peaksc5_kons=VT_peaksc5_kons + 2.
EXECUTE.

IF (VT_peak_low3_kons_adult > 0) VT_peaksc5_kons=VT_peaksc5_kons - 1.
EXECUTE.

IF (VT_peak_low2_kons_girl > 0) VT_peaksc5_kons=VT_peaksc5_kons + 2.
EXECUTE.

IF (VT_peak_low2_kons_boy > 0) VT_peaksc5_kons=VT_peaksc5_kons + 2.
EXECUTE.

IF (VT_peak_low2_kons_adult > 0) VT_peaksc5_kons=VT_peaksc5_kons - 1.
EXECUTE.

IF (VT_peak_mid2_kons_girl > 0) VT_peaksc5_kons=VT_peaksc5_kons + 1.
EXECUTE.

IF (VT_peak_mid2_kons_boy > 0) VT_peaksc5_kons=VT_peaksc5_kons + 1.
EXECUTE.

IF (VT_peak_mid2_kons_adult > 0) VT_peaksc5_kons=VT_peaksc5_kons - 0.5.
EXECUTE.


*****peak score RCP*****

COMPUTE VT_RCP_kons = 0.
EXECUTE.

IF (VT_peak_low3_kons_child > 0) VT_RCP_kons=VT_RCP_kons + 1.
EXECUTE.

IF (VT_peak_low2_kons_child > 0) VT_RCP_kons=VT_RCP_kons + 1.
EXECUTE.


****************peak-scoring with progressive values****************

*****peak score 1*****

COMPUTE VT_peaksc1_prog = 0.
EXECUTE.

IF (VT_peak_prog_girl > 0) VT_peaksc1_prog=VT_peaksc1_prog + 2.
EXECUTE.

IF (VT_peak_prog_boy > 0) VT_peaksc1_prog=VT_peaksc1_prog + 2.
EXECUTE.

IF (VT_peak_prog_adult > 0) VT_peaksc1_prog=VT_peaksc1_prog -1.
EXECUTE.

IF (VT_peak_low2_prog_girl > 0) VT_peaksc1_prog=VT_peaksc1_prog + 1.
EXECUTE.

IF (VT_peak_low2_prog_boy > 0) VT_peaksc1_prog=VT_peaksc1_prog + 1.
EXECUTE.

IF (VT_peak_low2_prog_adult > 0) VT_peaksc1_prog=VT_peaksc1_prog - 0.5.
EXECUTE.


*****peak score 2****

COMPUTE VT_peaksc2_prog = 0.
EXECUTE.

IF (VT_peak_prog_girl > 0) VT_peaksc2_prog=VT_peaksc2_prog + 2.
EXECUTE.

IF (VT_peak_prog_boy > 0) VT_peaksc2_prog=VT_peaksc2_prog + 3.
EXECUTE.

IF (VT_peak_prog_adult > 0) VT_peaksc2_prog=VT_peaksc2_prog -1.
EXECUTE.

IF (VT_peak_low2_prog_girl > 0) VT_peaksc2_prog=VT_peaksc2_prog + 1.
EXECUTE.

IF (VT_peak_low2_prog_boy > 0) VT_peaksc2_prog=VT_peaksc2_prog + 1.5.
EXECUTE.

IF (VT_peak_low2_prog_adult > 0) VT_peaksc2_prog=VT_peaksc2_prog - 0.5.
EXECUTE.


*****peak score 3*****

COMPUTE VT_peaksc3_prog = 0.
EXECUTE.

IF (VT_peak_prog_girl > 0) VT_peaksc3_prog=VT_peaksc3_prog + 1.
EXECUTE.

IF (VT_peak_prog_boy > 0) VT_peaksc3_prog=VT_peaksc3_prog + 1.
EXECUTE.

IF (VT_peak_prog_adult > 0) VT_peaksc3_prog=VT_peaksc3_prog -0.5.
EXECUTE.

IF (VT_peak_low3_prog_girl > 0) VT_peaksc3_prog=VT_peaksc3_prog + 1.
EXECUTE.

IF (VT_peak_low3_prog_boy > 0) VT_peaksc3_prog=VT_peaksc3_prog + 1.
EXECUTE.

IF (VT_peak_low3_prog_adult > 0) VT_peaksc3_prog=VT_peaksc3_prog - 0.5.
EXECUTE.

IF (VT_peak_low2_prog_girl > 0) VT_peaksc3_prog=VT_peaksc3_prog + 1.
EXECUTE.

IF (VT_peak_low2_prog_boy > 0) VT_peaksc3_prog=VT_peaksc3_prog + 1.
EXECUTE.

IF (VT_peak_low2_prog_adult > 0) VT_peaksc3_prog=VT_peaksc3_prog - 0.5.
EXECUTE.


*****peak score 4*****

COMPUTE VT_peaksc4_prog = 0.
EXECUTE.

IF (VT_peak_prog_girl > 0) VT_peaksc4_prog=VT_peaksc4_prog + 1.
EXECUTE.

IF (VT_peak_prog_boy > 0) VT_peaksc4_prog=VT_peaksc4_prog + 1.
EXECUTE.

IF (VT_peak_prog_adult > 0) VT_peaksc4_prog=VT_peaksc4_prog -0.5.
EXECUTE.

IF (VT_peak_low3_prog_girl > 0) VT_peaksc4_prog=VT_peaksc4_prog + 1.
EXECUTE.

IF (VT_peak_low3_prog_boy > 0) VT_peaksc4_prog=VT_peaksc4_prog + 1.
EXECUTE.

IF (VT_peak_low3_prog_adult > 0) VT_peaksc4_prog=VT_peaksc4_prog - 0.5.
EXECUTE.

IF (VT_peak_low2_prog_girl > 0) VT_peaksc4_prog=VT_peaksc4_prog + 1.
EXECUTE.

IF (VT_peak_low2_prog_boy > 0) VT_peaksc4_prog=VT_peaksc4_prog + 1.
EXECUTE.

IF (VT_peak_low2_prog_adult > 0) VT_peaksc4_prog=VT_peaksc4_prog - 0.5.
EXECUTE.

IF (VT_peak_mid2_prog_girl > 0) VT_peaksc4_prog=VT_peaksc4_prog + 1.
EXECUTE.

IF (VT_peak_mid2_prog_boy > 0) VT_peaksc4_prog=VT_peaksc4_prog + 1.
EXECUTE.

IF (VT_peak_mid2_prog_adult > 0) VT_peaksc4_prog=VT_peaksc4_prog - 0.5.
EXECUTE.


*****peak score 5*****

COMPUTE VT_peaksc5_prog = 0.
EXECUTE.


IF (VT_peak_prog_girl > 0) VT_peaksc5_prog=VT_peaksc5_prog + 1.
EXECUTE.

IF (VT_peak_prog_boy > 0) VT_peaksc5_prog=VT_peaksc5_prog + 1.
EXECUTE.

IF (VT_peak_prog_adult > 0) VT_peaksc5_prog=VT_peaksc5_prog -0.5.
EXECUTE.

IF (VT_peak_low3_prog_girl > 0) VT_peaksc5_prog=VT_peaksc5_prog + 2.
EXECUTE.

IF (VT_peak_low3_prog_boy > 0) VT_peaksc5_prog=VT_peaksc5_prog + 2.
EXECUTE.

IF (VT_peak_low3_prog_adult > 0) VT_peaksc5_prog=VT_peaksc5_prog - 1.
EXECUTE.

IF (VT_peak_low2_prog_girl > 0) VT_peaksc5_prog=VT_peaksc5_prog + 2.
EXECUTE.

IF (VT_peak_low2_prog_boy > 0) VT_peaksc5_prog=VT_peaksc5_prog + 2.
EXECUTE.

IF (VT_peak_low2_prog_adult > 0) VT_peaksc5_prog=VT_peaksc5_prog - 1.
EXECUTE.

IF (VT_peak_mid2_prog_girl > 0) VT_peaksc5_prog=VT_peaksc5_prog + 1.
EXECUTE.

IF (VT_peak_mid2_prog_boy > 0) VT_peaksc5_prog=VT_peaksc5_prog + 1.
EXECUTE.

IF (VT_peak_mid2_prog_adult > 0) VT_peaksc5_prog=VT_peaksc5_prog - 0.5.
EXECUTE.


*****peak score RCP*****

COMPUTE VT_RCP_prog = 0.
EXECUTE.

IF (VT_peak_low3_prog_child > 0) VT_RCP_prog=VT_RCP_prog + 1.
EXECUTE.

IF (VT_peak_low2_prog_child > 0) VT_RCP_prog=VT_RCP_prog + 1.
EXECUTE.
```
